# Pyridyl‐Thiazole‐Thiosemicarbazones as Antitrypanosomatidae Agents: Design, Synthesis, and In Silico Profiling

**DOI:** 10.1002/cmdc.70443

**Published:** 2026-08-21

**Authors:** Anderson José Firmino Santos da Silva, Daiane Santiago da Cruz Olimpio, Lucas Manoel da Silva Sousa, Graziella Leite Brondani, Tacylla Lima Silva, Wéllyda Belmira de Lira Silva, Natáli Tereza Capistrano Costa, Marcelo Zaldini Hernandes, Policarpo Ademar Sales Junior, Valéria Rêgo Alves Pereira, Ana Cristina Lima Leite

**Affiliations:** ^1^ Department of Pharmaceutical Sciences Health Sciences Centre Federal University of Pernambuco Recife Pernambuco Brazil; ^2^ Aggeu Magalhães Institute Fundação Oswaldo Cruz (FIOCRUZ) Recife Pernambuco Brazil

**Keywords:** *Leishmania* spp, pyridine, thiazole, thiosemicarbazone, *Trypanosoma cruzi*

## Abstract

Chagas disease and leishmaniasis are neglected tropical diseases caused by the trypanosomatid protozoa *Trypanosoma cruzi* and *Leishmania* spp., respectively. Currently available treatments are often insufficiently effective and lack the characteristics of ideal therapeutics. Here we report the design, synthesis, and antitrypanosomatid evaluation of 20 novel hybrid compounds incorporating pyridine, thiazole, and thiosemicarbazone scaffolds. All compounds were tested against trypomastigote and amastigote forms of *T. cruzi*, as well as promastigote and amastigote forms of *Leishmania infantum* and *L. amazonensis*. Cytotoxicity was assessed in L929 fibroblasts and RAW 264.7 macrophages. Compound 8 emerged as the lead, displaying an IC_50_ of 1.0 µM against *T. cruzi* and a selectivity index of 52.8. Despite its potent in vitro activity, Compound 8 exhibited poor in vivo efficacy when administered orally at 150 mg/kg/day. In silico analyses predicted strong binding to cruzain, alongside favorable bioavailability, drug‐likeness, and chemical stability. Against *Leishmania* species, Compound 8 demonstrated selectivity comparable to miltefosine against *L. amazonensis* amastigotes. Collectively, these findings identify a novel chemical scaffold with promising antitrypanosomatid potential that warrants further optimization to improve in vivo performance.

## Graphical abstract

A novel pyridine–thiazole derivative, Compound 8, exhibited potent dual antitrypanosomatid activity. It demonstrated remarkable in vitro efficacy against *Trypanosoma cruzi* (IC_50_ = 1.0 µM) and *L. amazonensis* (IC_50_ = 3.9 µM). Molecular docking studies suggested interactions with heme and multiple molecular targets. Compound 8 was selected for evaluation in in vivo infection models against *T. cruzi*.

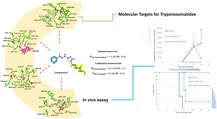

## 1 Introduction

Chagas disease and leishmaniasis are neglected tropical diseases (NTDs) [1] that pose significant public health challenges. Chagas disease, or American trypanosomiasis, is caused by *Trypanosoma cruzi* [2] and affects approximately 6–7 million people worldwide [3]. The disease progresses through acute and chronic phases, with infection control dependent on both innate and adaptive immune responses [4]. Leishmaniasis, caused by *Leishmania* parasites transmitted by female sandflies [5], is endemic in 98 countries across Europe, Africa, Asia, and South America, with more than 90% of new cases occurring in just 13 countries [6]. Currently available drugs for the prophylaxis and treatment of these neglected diseases are frequently associated with high toxicity and numerous adverse side effects [7].

Moreover, drug discovery for these kinetoplastid parasites is hindered by significant translational challenges, including the frequent disconnect between in vitro potency and in vivo efficacy, which often arises from poor pharmacokinetic (PK) properties, rapid metabolism, or inadequate tissue distribution [8, 9]. Contemporary hit‐to‐lead strategies now emphasize early integration of ADME (absorption, distribution, metabolism, excretion) profiling and in vivo PK studies to mitigate risk during candidate progression [10, 11].

Given the significant limitations of existing therapies, our group, the Medicinal Chemistry Research Laboratory (LPQM), has been dedicated to the design and synthesis of new chemical entities, employing privileged scaffolds as a strategic foundation for molecular design. Among the heterocyclic cores most frequently utilized in our design of novel drug prototypes are pyridines and thiazoles. We previously identified 2‐pyridyl thiazoles as potential *T. cruzi* inhibitors [12, 13], and more recently, Silva et al. (2025) demonstrated that 3‐pyridyl‐1,3‐thiazoles exhibit both anti‐*T. cruzi* and leishmanicidal activities [14]. In parallel, thiosemicarbazones represent a tractable scaffold with demonstrated in vitro activity against *Leishmania* spp. and emerging reports of activity against *T. cruzi* [15, 16]. However, their redox/metal‐binding mechanisms can introduce metabolic and safety liabilities, including rapid clearance, off‐target toxicity, and potential for oxidative stress in host cells that necessitate focused structure–activity relationship (SAR) studies and rigorous pharmacokinetic characterization during hit‐to‐lead optimization [17, 18].

Building on our earlier findings that 2‐ and 3‐pyridyl‐1,3‐thiazoles display promising anti‐*T. cruzi* and leishmanicidal properties, we designed a new class of 3‐ and 4‐pyridyl‐1,3‐thiazoles incorporating a thiosemicarbazone moiety, thereby combining two complementary privileged structural motifs into a single scaffold. The pyridyl–thiazole core provides a compact, drug‐like framework with established antiparasitic potential, while the thiosemicarbazone fragment is expected to enhance anti‐*T. cruzi* activity through metal chelation and redox cycling processes that disrupt iron‐dependent metabolic pathways and increase oxidative stress within the parasite. This structural modification is hypothesized to support multitarget activity, potentially reducing the risk of resistance emergence and maintaining efficacy across multiple parasitic life stages [19, 20, 21, 22, 23]. This hybrid approach aligns with modern medicinal chemistry strategies aimed at developing multitarget agents, which may help mitigate resistance and sustain efficacy across diverse parasitic life stages [24, 25].

**FIGURE 1:**
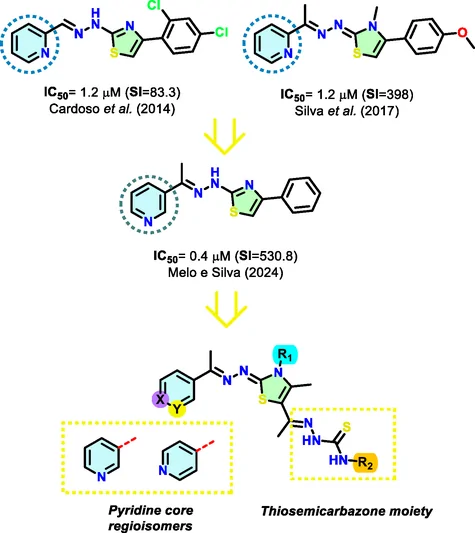
Generic representation of new synthesized compounds.

With the aim of identifying compounds with antitrypanosomatid activity, this work describes the structural design, synthesis, and in vitro, in vivo, and in silico evaluation of novel 3‐ and 4‐pyridyl‐1,3‐thiazoles bearing a thiosemicarbazone moiety (6–20). In the structural design, we explored CH/N bioisosteric replacement within the pyridyl ring, as well as the introduction of hydrogen (H), phenyl (Ph), methyl (Me), and ethyl (Et) substituents at the N3 position of the thiazole ring and at the N3 position of the thiosemicarbazone fragment (Figure 1).

The antitrypanosomatid activity of these novel compounds was evaluated in vitro against *T. cruzi*, *L. amazonensis*, and *L. infantum*. Cytotoxicity was also assessed using L929 fibroblasts and RAW 264.7 macrophages. The most promising compound of the series was further examined for its ability to reduce parasitemia and mortality in male Swiss mice infected with trypomastigotes of the partially drug‐resistant Y strain. In silico studies were performed to elucidate the interactions of the compounds with key molecular targets relevant to both *T. cruzi* and *Leishmania*. Additionally, the newly synthesized 3‐ and 4‐pyridyl‐thiazole–thiosemicarbazones were characterized with respect to their physicochemical properties and ADME profiles. Compound 8 combined high in vitro potency with favorable in silico properties and therefore met our prespecified criteria for early in vivo proof‐of‐concept evaluation. We selected Compound 8 following the minimum criteria and decision gates recommended for Chagas drug discovery [26, 27].

## 2 Results and Discussion

Our approach encompasses the synthesis and structural characterization of 20 new compounds, followed by a stepwise biological evaluation. Cytotoxicity was first assessed in L929 fibroblasts and RAW 264.7 macrophages to establish selectivity and identify suitable candidates for further investigation. In vitro antitrypanosomatid activity was then evaluated by comparing efficacy against different life stages of *T. cruzi*, *L. amazonensis*, and *L. infantum*, with particular emphasis on emerging structure–activity relationships. Based on the in vitro results, the most promising compound was advanced to in vivo evaluation in a murine model of Chagas disease using the partially drug‐resistant Y strain of *T. cruzi*. To support these findings, in silico studies were performed to investigate interactions with key molecular targets relevant to both parasites. Finally, physicochemical and ADME properties were analyzed to assess drug‐likeness and to identify potential liabilities affecting oral exposure and translational potential.

### 2.1 Chemistry

**SCHEME 1:**
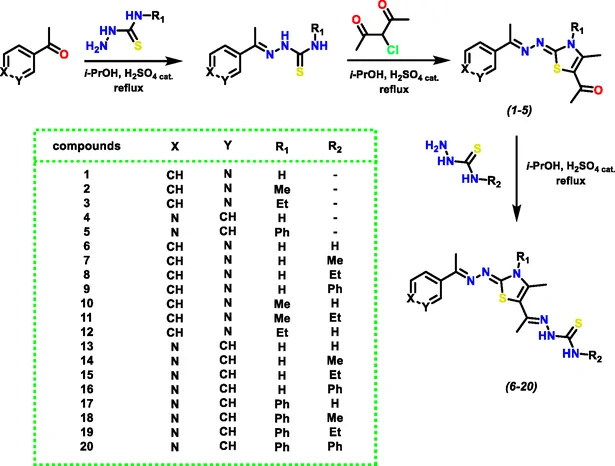
Synthesis of compounds 1–20.

To synthesize our set of 20 molecules, we used 3‐acetylpyridine or 4‐acetylpyridine as starting materials. All synthetic procedures described in this study are summarized in Scheme 1. Briefly, intermediate compounds 1–5 were obtained through a two‐step process, whereas compounds 6–20 required three steps. In the first step, the pyridines were dissolved in isopropyl alcohol and reacted with various commercially available thiosemicarbazides (1:1 molar ratio) via Schiff base condensation under reflux in the presence of a catalytic amount of sulfuric acid. This reaction afforded pure intermediates in excellent yields (>96%). In the second step, these intermediates were reacted with a slight excess of 3‐chloro‐2,4‐pentanedione (1:1.2 molar ratio) in isopropyl alcohol under reflux at 75 °C for 3 h, again using a catalytic amount of sulfuric acid. This procedure yielded five novel thiazole derivatives (compounds 1–5) with high purity and good yields. In the final step, the five thiazoles obtained were further reacted with different thiosemicarbazides under the same conditions used in the first step, producing compounds 6–20 with yields ranging from 80% to 95%. The chemical structures of all synthesized compounds were confirmed by nuclear magnetic resonance spectroscopy (^1^H and ^13^C NMR).

### 2.2 Cytotoxicity Activity Against L929 Cells

Following structural characterization of the intermediate compounds (1–5) and the pyridyl–thiazole derivatives (6–20), their cytotoxic profiles were evaluated in L929 cells. Cytotoxicity was assessed by determining the concentration required to reduce cell viability by 50% (CC_50_), calculated as the difference in viability between treated and untreated cells. As shown in Table 1, all tested compounds exhibited greater cytotoxicity than benznidazole (CC_50_ = 1105.1 µM).). Among them, intermediate compounds 3–5 and the newly synthesized 3‐ and 4‐pyridyl–thiazole–thiosemicarbazones 7, 8, 12, 14, and 20 displayed the highest cytotoxicity, with CC_50_ values below 100 µM. Although compounds 13, 15, 16, 17, and 19 showed CC_50_ values above 200 µM, benznidazole remained the least cytotoxic compound among those tested.

**TABLE 1:** Cytotoxicity and antiparasitic activity against trypomastigote and amastigote forms of *T. cruzi.*

Compound	**CC**_**50**_ **,** **µM** **L929** **[95% CI]**	**IC_50_, µM** **amastigotes/** **trypomastigotes** **(Tulahuen strain)** **[95% CI]**	**LC**_**50**_ **,** **µM** **trypomastigotes** **(Y strain)** **[95% CI]**	**Selectivity** **index** **(CC**_**50**_ **/IC**_**50**_ **)**
**1**	121.6 ± 2.8** [93.0–168.1]	60.0 ± 32.0** [56.0–64.2]	94.5 ± 4.6** [85.3–106.1]	2.0
**2**	108.2 ± 9.3** [81.5–142.5]	12.2 ± 3.5 [10.8–13.6]	103.4 ± 3.5** [78.0–137.3]	8.9
**3**	47.9 ± 16.4** [38.4–59.1]	13.1 ± 7.5 [11.9–14.4]	64.3 ± 2.1** [43.3–90.9]	3.7
**4**	45.1 ± 4.9** [39.7–50.7]	45.6 ± 2.6** [42.3–48.5]	Inactive	1.0
**5**	33.4 ± 3.3** [25.1–43.1]	11.1 ± 0 [10.3–11.7]	37.6 ± 2.2 [30.8–44.8]	3.0
**6**	92.6 ± 34.4** [44.6–250.7]	110.3 ± 1.2** [86.9–168.7]	94.1 ± 14.3** [53.5–159.7]	0.8
**7**	51.9 ± 0.2** [37.9–71.1]	10.9 ± 4.8 [9.6–12.5]	28.8 ± 9.8 [23.2–33.2]	4.8
**8**	52.8 ± 6.0** [34.6–81.2]	1.0 ± 0.1 [0.8–1.2]	0.3 ± 0 [0.3–0.4]	52.8
**9**	69.0 ± 9.4** [51.9–90.0]	8.6 ± 4.0 [6.9–10.2]	35.6 ± 2.2 [22.2–56.4]	8.0
**10**	69.5 ± 5.9** [40.4–127.8]	13.1 ± 6.0 [11.4–14.8]	62.7 ± 49.9** [37.1–93.2]	5.3
**11**	95.1 ± 5.2** [72.9–123.0]	10.9 ± 1.6 [8.7–13.1]	28.3 ± 0.4 [20.5–38.5]	8.7
**12**	39.7 ± 0.7** [35.7–43.9]	7.6 ± 0.1 [6.9–8.0]	51.2 ± 0.8* [45.3–58.1]	5.2
**13**	>286.1	162.5 ± 5.7** [NE]	Inactive	>1.8
**14**	25.6 ± 10.3** [5.5–25.7]	10.0 ± 1.6 [8.3–11.6]	Inactive	2.6
**15**	212.8 ± 12.8** [NE]	9.5 ± 2.1 [7.2–12.8]	24.8 ± 15.4 [9.6–47.1]	22.4
**16**	>236.1	25.2 ± 1.2** [23.4–27.2]	Inactive	>9.4
**17**	>236.1	6.1 ± 3.7 [3.5–8.0]	10.4 ± 1.7 [8.0–13.2]	>38.7
**18**	178.2 ± 2.1** [NE]	10.0 ± 1.5 [7.1–13.9]	12.4 ± 8.1 [5.9–23.1]	17.8
**19**	207.6 ± 19.5** [NE]	4.2 ± 0.3 [4.0–4.4]	8.2 ± 1.6 [6.0‐NE]	49.4
**20**	54.6 ± 7.4** [35.2–86.5]	9.3 ± 1.6 [7.2–12.0]	36.5 ± 3.3 [31.0–39.6]	5.9
**BZ**	1105.1 ± 79.6 [967.6–1257.1]	2.0 ± 0.4 [2.1–2.5]	7.9 ± 1.8 [6.0–9.4]	552.5

*Note:* CC_50_: concentration of the compound that inhibits the viability of L929 cells by 50%. IC_50_: concentration of the compound that reduces parasitic growth of the Tulahuen strain by 50%. LC_50_: concentration of the lethal compound for 50% of the trypomastigotes of the Y strain of *T. cruzi*. CC_50_/IC_50_/LC_50_ values are expressed as mean ± standard deviation of two technical replicates and were calculated by nonlinear regression analysis using GraphPad Prism 8.0.1 software. NE (not estimable): 95% CI could not be estimated for some values due to lack of convergence of nonlinear regression fitting. CC_50_/IC_50_/LC_50_ values for the test compounds were compared with those of benznidazole (control) using one‐way ANOVA followed by Dunnett's multiple comparisons test. *p < 0.05. **p < 0.01. BZ, benznidazole*.*

### 2.3 Antiparasitic Activity Against Trypomastigote and Amastigote Forms of *T.* *cruzi*

The IC_50_ values and selectivity indices (SI) were determined against *T. cruzi* using the Tulahuen strain (amastigote and trypomastigote forms) and the Y strain (trypomastigote form), with benznidazole used as the reference drug. As shown in Table 1, Compound 8 exhibited the most promising trypanocidal profile, with an IC_50_ of 1.0 µM and a selectivity index of 52.8 against the Tulahuen strain, and an LC_50_ of 0.3 µM against the Y strain. By comparison, benznidazole displayed IC_50_ values of 2.0 µM against the Tulahuen strain and a LC_50_ of 7.9 µM against the Y strain.

**FIGURE 2:**
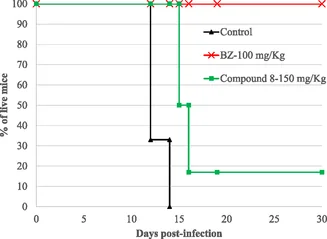
Survival assessment of male Swiss mice infected with 10,000 trypomastigotes of the Y strain of *Trypanosoma cruzi* and treated with Benznidazole (BZ) or molecule 8, as well as Controls treated only with the vehicle (10% DMSO, 20% PEG 400, and 0.05% Tween 80 in PBS), orally.

In general, the 3‐ and 4‐pyridyl‐1,3‐thiazoles bearing a thiosemicarbazone moiety (compounds 6–20) exhibited superior trypanocidal activity compared to the intermediate 3‐pyridyl‐thiazole methyl ketones (compounds 1–5) (Figure 2). Among compounds 6–20, seven derivatives showed IC_50_ values below 10 µM (ranging from 9.5 to 1.0 µM) against the Tulahuen strain (amastigote and trypomastigote forms). In addition, only two compounds displayed LC_50_ values below 10 µM (ranging from 8.2 to 0.3 µM) when evaluated against the Y strain trypomastigote form.

During the structural design of compounds 6–20, we investigated CH/N bioisosteric replacement within the pyridyl ring, as well as the introduction of hydrogen (H), phenyl (Ph), methyl (Me), and ethyl (Et) substituents at the N3 position of the thiazole ring and at the N3 position of the thiosemicarbazone moiety. Compound 8, a 3‐pyridyl‐1,3‐thiazole bearing a thiosemicarbazone moiety with no substituent at the N3 position of the thiazole ring and an ethyl group at the N3 position of the thiosemicarbazone, demonstrated the most favorable trypanocidal profile against both the Tulahuen and Y strains.

The incorporation of a thiosemicarbazone moiety into the pyridyl‐thiazole scaffold may favorably influence physicochemical and biological properties that enhance trypanocidal activity. Thiosemicarbazones contain multiple heteroatoms and a conjugated framework that can modulate lipophilicity and polarity, improve membrane permeability, and facilitate parasite cell penetration and intracellular compound accumulation. Moreover, the extended conjugation and potential for multiple tautomeric forms can stabilize bioactive conformations and promote favorable interactions with biological targets, contributing to enhanced efficacy compared with simple ketone intermediates [28, 29].

A clearer trend emerges when considering pyridyl regioisomerism, N‐substitution, lipophilicity, sterics, and H‐bonding together. Both 3‐ and 4‐pyridyl isomers can be active, but the best performer (Compound 8) is a 3‐pyridyl derivative. We propose that the 3‐pyridyl orientation favorably positions the ring nitrogen for dipolar/H‐bond interactions or for directing the electronic density of the scaffold in a manner that enhances target engagement, whereas the 4‐pyridyl vector alters interaction geometry and can reduce potency [30].

N‐substitution and steric effects show complementary influences [31]. Across the thiazole series, minimal substitution at the thiazole N3 (as in 8) correlates with higher activity, suggesting a sterically sensitive binding pocket was preserving planarity and avoiding local steric clash is beneficial. By contrast, small alkylation at the thiosemicarbazone N3 (Et in 8) generally improves potency relative to H or very bulky groups, indicating that modest increase in lipophilicity at this position enhances membrane permeability and/or favorable hydrophobic contacts without causing steric interference [32].

The thiosemicarbazone fragment appears to be a key driver of activity relative to the ketone intermediates. Its multiple heteroatoms, potential for tautomerism, and extended conjugation increase H‐bonding capacity and conformational flexibility, which can stabilize productive binding poses or permit metal‐binding interactions if relevant [33]. Compound 8 likely achieves a favorable polarity/lipophilicity balance (adequate membrane permeability and intracellular accumulation with maintained solubility), explaining its improved potency and acceptable cytotoxicity compared with more hydrophobic or bulkier analogs that trend toward increased host toxicity.

### 2.4 Predicted DMPK Profiling of Compound 8 and In Vivo Activity

#### 2.4.1 Predicted DMPK Analysis of Compound 8

Predictive deep learning–based approaches were employed to estimate the pharmacokinetic profile of Compound 8 using Deep‐PK [34]. This computational strategy enables the identification of relevant pharmacokinetic parameters while reducing experimental costs and supporting the principles of the 3Rs (Replacement, Reduction, and Refinement) in drug discovery [35, 36, 37]. Pharmacokinetic predictions were generated using graph neural network architectures and graph‐based molecular representations and were subsequently compared with those obtained for benznidazole. As shown in Table 2, Compound 8 exhibited an overall pharmacokinetic profile comparable to that predicted for benznidazole, suggesting that the structural modifications introduced in this series did not substantially compromise its drug‐like properties. Both compounds were predicted to possess favorable oral bioavailability and low susceptibility to P‐glycoprotein**–**mediated efflux, supporting their potential for oral administration.

**TABLE 2:** Predicted DMPK properties of Compound 8 and benznidazole.

	Parameter	Compound 8	Benznidazole
Absorption	Caco‐2 (logPapp)	−4.96	−4.47
	Oral bioavailability ≥50%	Yes	Yes
	P‐gp inhibitor	Yes	No
	P‐gp substrate	No	No
Distribution	Fraction unbound (Fu)	0.66	0.83
	Plasma protein binding (%)	76.17	51.31
	VDss (log L/kg)	0.43	0.67
Metabolism	CYP1A2 inhibitor	Yes	No
	CYP2C19 inhibitor	Yes	No
	CYP2C9 inhibitor	Yes	No
	CYP3A4 inhibitor	Yes	No
	CYP3A4 substrate	No	Yes
Excretion	CL_T_ (log mL/min/kg)	5.17	2.28
	Half‐life	<3 h	<3 h

Despite these similarities, several differences were observed. Compound 8 exhibited a higher predicted plasma protein binding (76.17% vs. 51.31%) and a lower unbound fraction in plasma (Fu = 0.66 vs. 0.83), indicating a greater propensity to remain associated with circulating plasma proteins. Consistent with this finding, Compound 8 displayed a lower predicted steady‐state volume of distribution (VDss) than benznidazole, suggesting more limited tissue distribution. Nevertheless, both compounds were predicted to have short elimination half‐lives (<3 h).

A notable distinction between the two molecules emerged in their predicted metabolic profiles. Compound 8 was classified as an inhibitor of CYP1A2, CYP2C19, CYP2C9, and CYP3A4, whereas benznidazole was predicted to act as a CYP3A4 substrate without significant inhibitory activity toward the major CYP isoforms. Interestingly, despite its lower predicted tissue distribution, Compound 8 exhibited a higher predicted total clearance than benznidazole. This finding may reflect enhanced metabolic and/or excretory elimination and could partially offset the effects of its greater plasma protein binding. Overall, the predicted DMPK parameters indicate that Compound 8 retains pharmacokinetic characteristics broadly comparable to those of benznidazole.

### 2.5 Acute Toxicity in Mice

After the minimum criteria and decision gates for Chagas disease hit prioritization [26, 27], Compound 8 was selected for an early in vivo proof‐of‐concept study based on its robust in vitro efficacy and supportive in silico predictions. The initial in vivo studies focused on assessing acute oral toxicity in Swiss mice of both sexes. To determine the maximum tolerated dose (MTD), two uninfected Swiss Webster mice (one male and one female), 8 weeks old, were used. On day 1, an initial oral dose of 5 mg/kg was administered. After 2 h, an additional dose of 15 mg/kg was given (cumulative dose [CD] = 20 mg/kg). Two h later, a further dose of 30 mg/kg was administered (CD = 50 mg/kg), followed by 50 mg/kg (CD = 100 mg/kg), and finally 100 mg/kg (CD = 200 mg/kg) after another 2‐h interval. On day 2, the animals were monitored for signs of toxic or subtoxic effects in accordance with the guidelines of the Organization for Economic Co‐operation and Development (OECD) [38]. No clinical signs of toxicity or subtoxicity were observed during dosing or within 2 days after treatment. Consequently, the MTD of Compound 8 following oral administration was established at 200 mg/kg.

### 2.6 Determination of Parasitemia and Survival

**FIGURE 3:**
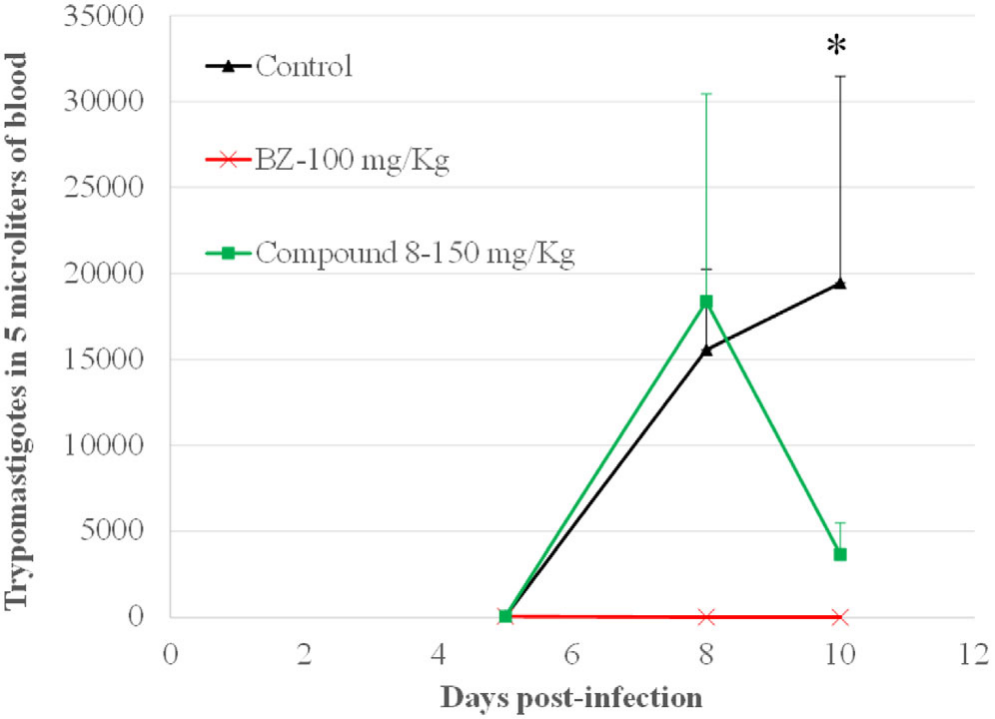
Evaluation of parasitemia in male Swiss mice infected with 10,000 trypomastigotes of the Y strain of *Trypanosoma cruzi* and treated with Benznidazole (BZ) or Compound 8, as well as Controls treated only with the vehicle (10% DMSO, 20% PEG 400, and 0.05% Tween 80 in PBS), orally. Treatment, performed every 24 h, extended from the fifth to the ninth day postinfection. Each point on the graph represents the mean plus standard deviation of 6 animals per group. The asterisk indicates a statistically significant difference (*p* < 0.05), calculated by Student's t‐test, between the Control group and the group treated with the Compound 8.

Treatment was administered once every 24 h from day 5 to day 9 post‐infection, with six animals included in each group. Survival curves were analyzed using the log‐rank test, revealing that both the benznidazole‐ and Compound 8‐treated groups exhibited significantly higher survival rates than the control group (*p* < 0.05). However, the benznidazole‐treated group showed significantly greater survival than the group treated with Compound 8 (*p* < 0.05). Parasitemia levels in the Compound 8‐treated group were lower than those in the control group on day 10 postinfection, corresponding to an 81.3% reduction, and treated animals demonstrated increased survival. Nevertheless, Compound 8 was less effective than the reference drug benznidazole: 83% of animals in the Compound 8 group died, whereas no mortality was observed in the benznidazole‐treated group, and parasitemia levels were higher in the Compound 8 group on days 8 and 10 postinfection. Overall, these results indicate that compound 8 exhibited limited in vivo efficacy in mice when administered orally at a dose of 150 mg/kg for five consecutive days (Figures 2 and 3).

The results presented above indicate that pyridyl‐1,3‐thiazole Compound 8 exhibited limited in vivo efficacy in mice when administered orally at 150 mg/kg for five consecutive days, despite demonstrating potent trypanocidal activity in vitro. This loss of antiparasitic capability upon oral administration highlights a common translational challenge in drug discovery. While in vitro assays are essential for preliminary activity screening and mechanistic exploration, they inevitably oversimplify biological complexity [39] by failing to reproduce the interplay of diverse cellular processes, tissue architecture, dynamic immune responses, and host ADME barriers [40]. Consequently, predictions of in vivo performance based solely on in vitro data are inherently limited [41], underscoring the critical importance of oral stability—ensuring that a drug reaches its target site at effective concentrations without degradation in the gastrointestinal tract or rapid first‐pass hepatic metabolism [42].

The clear disconnect between the potent in vitro activity of Compound 8 and its lack of oral in vivo efficacy most likely reflects underlying exposure, ADME, or physicochemical liabilities such as poor oral bioavailability, low aqueous solubility, rapid metabolic clearance, high plasma protein binding, limited tissue penetration (notably to cardiac reservoirs in *T. cruzi* infection), or efflux‐mediated loss of intracellular accumulation rather than definitive failure of the chemotype [43, 44, 45]. In this study, comprehensive DMPK/PK profiling was not conducted prior to the efficacy experiment due to practical constraints that would have prevented generation of robust, interpretable data. To avoid producing low‐confidence PK results that could mislead subsequent optimization efforts, we instead performed a focused, small‐scale in vivo efficacy study using established local facilities. While this approach allowed timely evaluation of antiparasitic activity, the absence of exposure data limits our ability to determine whether the negative outcome reflects intrinsic lack of in vivo potency or simply insufficient drug exposure at the site of infection.

Overcoming such translational gaps requires the adoption of standardized, exposure‐aware screening cascades that integrate early PK/PD profiling, tissue distribution studies (including bioluminescence imaging where available for Chagas models), clear progression criteria, and transparent reporting of both positive and negative in vivo results [9, 46, 47]. Guided by these principles, medicinal chemistry optimization can be pursued rationally through advanced pharmaceutical formulations (e.g., nanoparticle encapsulation, controlled‐release systems, or gastro‐resistant coatings that protect the drug from harsh gastrointestinal environments while enhancing absorption and bioavailability), prodrug strategies, dosing regimen adjustments, or scaffold redesign before committing to larger‐scale animal or clinical studies. This integrated, data‐driven approach will maximize the probability of advancing promising antiparasitic candidates while minimizing unnecessary animal use and resource expenditure [48].

### 2.7 Cytotoxicity Activity Against RAW Cells

As shown in Table 3, compounds 6 (146.3 µM), 8 (124.7 µM), 11 (105.3 µM), 13 (>286.1 µM), 17 (>236.1 µM), and 20 (103.3 µM) exhibited the highest CC_50_ values. Notably, eighteen compounds displayed higher CC_50_ values than the reference drug miltefosine (CC_50_ = 30.4 µM).

**TABLE 3:** Biological activity of pyridine‐thiazole‐thiosemicarbazone against *L. amazonensis*.

Compound	**CC**_**50**_ **RAW,** **µM** **[95% CI]**	*Leishmania amazonensis*			
		**IC**_**50**_ **promastigotes,** **µM** **[95% CI]**	**SI** **promastigotes** **(CC**_**50**_ **/IC**_**50**_ **)**	**IC**_**50**_ **amastigotes,** **µM** **[95% CI]**	**SI** **amastigotes** **(CC**_**50**_ **/IC**_**50**_ **)**
**1**	76.8 ± 1.3** [73.3–87.9]	>364.5	ND	211.3 ± 7.8** [NE]	0.4
**2**	81.9 ± 2.5** [73.5–94.3]	245.4 ± 0.2** [NE]	0.3	31.2 ± 15.4 [15.1–52.5]	2.6
**3**	52.1 ± 5.4 [46.0–58.5]	Inactive	ND	51.4 ± 11.8* [29.7–83.4]	1.0
**4**	73.5 ± 5.5** [69.7–77.0]	Inactive	ND	Inactive	ND
**5**	81.2 ± 1.6** [NE‐87.1]	69.0 ± 2.6** [55.4–84.2]	1.2	60.2 ± 3.3** [54.5–65.8]	1.3
**6**	146.3 ± 35.0** [109.7‐NE]	Inactive	ND	97.0 [14.6‐NE]	1.5
**7**	49.0 ± 16.8 [30.7–70.6]	>276.6	ND	Inactive	ND
**8**	124.7 ± 1.5** [96.4–153.9]	53.3 ± 2.6** [42.3–66.8]	2.3	3.9 ± 0.3 [3.0–5.4]	32.0
**9**	19.0 ± 2.8 [15.4–25.5	Inactive	ND	127.3 ± 6.2** [Very wide]	0.2
**10**	49.0 ± 2.8 [39.6–60.9]	Inactive	ND	42.0 ± 30.5 [13.3–120.7]	1.2
**11**	105.3 ± 14.9** [89.6–122.5]	118.8 ± 23.4** [107.3–132.7]	0.9	62.5 ± 33.2** [40.9–107.8]	1.7
**12**	73.9 ± 1.7** [62.3–86.8]	54.6 ± 4.5** [NE]	1.4	14.2 ± 3.5 [9.5–18.8]	5.2
**13**	>286.1	Inactive	ND	122.5 ± 5.0** [88.6–163.2]	>2.3
**14**	45.8 ± 5.6 [15.1–316.2]	Inactive	ND	>276.6	<0.2
**15**	79.5 ± 2.8** [53.8–115.1]	Inactive	ND	71.4 ± 20.0** [57.6–99.3]	1.1
**16**	57.0 ± 20.1 [31.7–105.8]	Inactive	ND	34.9 ± 1.6 [22.5–55.2]	1.6
**17**	>236.1	Inactive	ND	21.4 ± 3.2 [16.9–26.8]	>11.0
**18**	77.4 ± 0.8** [72.0–83.5]	26.2 ± 0.1 [22.2‐NE]	3.0	29.3 ± 17.4 [13.4–48.0]	2.6
**19**	44.1 ± 5.4 [40.7–48.5]	Inactive	ND	18.8 ± 4.2 [12.7–26.9]	2.3
**20**	103.3 ± 0.4** [Very wide]	Inactive	ND	40.0 ± 1.9 [30.7–51.6]	2.6
**Miltefosine**	30.4 ± 1.9 [26.5–35.8]	10.8 ± 4.7 [NE]	2.8	0.9 ± 0.2 [0.7‐NE]	33.8

*Note:* CC_50_: concentration of the compound that inhibits the viability of RAW 264.7 cells by 50%. IC_50_: concentration of the compound that reduces parasitic growth of the *Leishmania* spp. by 50%. SI: Selectivity Index. NE (Not Estimable): 95% CI could not be estimated for some values due to lack of convergence of nonlinear regression fitting. IC_50_ and CC_50_ values are expressed as mean ± standard deviation of two technical replicates and were calculated by nonlinear regression analysis using GraphPad Prism 8.0.1 software. CC_50_/IC_50_ values for the test compounds were compared with those of miltefosine (control) using one‐way ANOVA followed by Dunnett's multiple comparisons test. *p < 0.05. **p < 0.01. ND, not determined.

### 2.8 Antiparasitic Activity Against *L. amazonensis* and L. infantum

The 3‐ and 4‐pyridyl–thiazole–thiosemicarbazone derivatives were evaluated in vitro against *L* *. infantum* and *L. amazonensis* in both promastigote and amastigote forms. Although the promastigote assay provides useful preliminary information for comparative purposes, the intracellular amastigote is the clinically relevant stage in the mammalian host and therefore represents the most appropriate model for assessing antileishmanial potential. As shown in Table 3, the compounds were inactive or only weakly active against the promastigote form of *L. amazonensis*. In contrast, activity against the amastigote form was more pronounced, with Compound 8 showing the most promising profile (IC_50_ = 3.9 µM, SI = 32.0). Miltefosine exhibited the highest potency against this form (IC_50_ = 0.9 µM, SI = 33.8).

In assays against the promastigote form of *L.* *infantum*, Compound 8 displayed an IC_50_ value of 8.8 µM (SI = 14.2), outperforming miltefosine, which showed an IC_50_ of 16.6 µM and a selectivity index of 1.8. However, miltefosine was more active against the amastigote form of *L. infantum* (IC_50_ = 2.0 µM, SI = 15.2). In addition, compounds 17, 19, and 20 also demonstrated selectivity against the amastigote form of *L. infantum*, highlighting the greater relevance of this stage for evaluating therapeutic potential (Table 4).

**TABLE 4:** Biological activity of pyridine‐thiazole‐thiosemicarbazone against *Leishmania infantum.*

Compound	**CC**_**50**_ **RAW,** **µM** **[95% CI]**	*Leishmania infantum*			
		**IC**_**50**_ **promastigotes,** **µM** **[95% CI]**	**SI** **promastigotes** **(CC**_**50**_ **/IC**_**50**_ **)**	**IC**_**50**_ **amastigotes,** **µM** **[95% CI]**	**SI** **amastigotes** **(CC**_**50**_ **/IC**_**50**_ **)**
**1**	76.8 ± 1.3** [73.3–87.9]	>364.5	ND	Inactive	ND
**2**	81.9 ± 2.5** [73.5–94.3]	245.2 ± 0** [NE]	0.3	>346.8	ND
**3**	52.1 ± 5.4 [46.0–58.5]	113.6 ± 19.4** [97.9–134.6]	0.5	233.7 ± 0.2** [NE]	0.2
**4**	73.5 ± 5.5** [69.7–77.0]	Inactive	ND	Inactive	ND
**5**	81.2 ± 1.6** [NE‐87.1]	63.9 ± 16.5** [54.8–69.9]	1.3	28.5 ± 0 [21.2–37.6]	2.8
**6**	146.3 ± 35.0** [109.7‐NE]	>287.8	ND	Inactive	ND
**7**	49.0 ± 16.8 [30.7–70.6]	126.5 ± 12.9** [116.5–134.4]	0.4	Inactive	ND
**8**	124.7 ± 1.5** [96.4–153.9]	8.8 ± 0.4 [8.0–9.6]	14.2	37.7 ± 1.0 [32.0–45.5]	3.3
**9**	19.0 ± 2.8 [15.4–25.5	Inactive	ND	Inactive	ND
**10**	49.0 ± 2.8 [39.6–60.9]	>276.6	ND	Inactive	ND
**11**	105.3 ± 14.9** [89.6–122.5]	72.7 ± 1.1** [69.6–77.0]	1.4	23.9 ± 11.2 [11.0–52.6]	4.4
**12**	73.9 ± 1.7** [62.3–86.8]	59.3 ± 0.9** [53.1‐NE]	1.2	81.2 ± 46.6** [67.4–122]	0.9
**13**	>286.1	Inactive	ND	102.5 ± 18.6** [38.8–630.4]	> 2.8
**14**	45.8 ± 5.6 [15.1–316.2]	Inactive	ND	195.6 ± 0** [NE]	0.2
**15**	79.5 ± 2.8** [53.8–115.1]	Inactive	ND	>266.3	<0.3
**16**	57.0 ± 20.1 [31.7–105.8]	Inactive	ND	>236.1	<0.2
**17**	>236.1	181.3 ± 0** [NE]	>1.3	18.6 ± 1.2 [14.9–22.8]	>12.7
**18**	77.4 ± 0.8** [72.0–83.5]	24.7 ± 0 [Very wide]	3.1	11.8 ± 1.1 [9.9–14.1]	6.6
**19**	44.1 ± 5.4 [40.7–48.5]	57.7 ± 4.0** [55.4–60.5]	0.8	4.3 ± 0.6 [3.5–4.9]	10.3
**20**	103.3 ± 0.4** [Very wide]	101.7 ± 0** [Very wide]	1.0	5.6 ± 2.5 [2.8–9.4]	18.4
**Miltefosine**	30.4 ± 1.9 [26.5–35.8]	16.6 ± 17.3 [Very wide]	1.8	2.0 ± 0.6 [1.7–2.5]	15.2

*Note:* CC_50_: concentration of the compound that inhibits the viability of RAW 264.7 cells by 50%. IC_50_: concentration of the compound that reduces parasitic growth of the *Leishmania* spp. by 50%. SI: Selectivity Index. NE (Not Estimable): 95% CI could not be estimated for some values due to lack of convergence of nonlinear regression fitting. IC_50_ and CC_50_ values are expressed as mean ± standard deviation of two technical replicates and were calculated by nonlinear regression analysis using GraphPad Prism 8.0.1 software. CC_50_/IC_50_ values for the test compounds were compared with those of miltefosine (control) using one‐way ANOVA followed by Dunnett's multiple comparisons test. *p < 0.05. **p < 0.01. ND, not determined.

Our current intracellular assay cannot fully exclude effects on parasite differentiation or on extracellular forms, rather than direct activity against fully differentiated amastigotes. Tegazzini et al. (2016) developed a robust, high‐content in vitro *L. donovani* assay in which extracellular promastigotes are removed before compounds are added, and intramacrophage replication is then quantified, thereby minimizing signals from extracellular parasites or reinfection [49]. This approach highlights the safeguards, for example, infecting macrophages in bulk followed by careful wash steps or selective removal/killing of extracellular parasites, delaying treatment until amastigote differentiation is confirmed, and using replication markers (e.g., EdU) or washout controls to ensure that measured activity reflects effects on established intracellular amastigotes. This discussion emphasizes the strengths and limitations of different intracellular screening models and outlines how follow‐up studies will more rigorously evaluate antileishmanial activity.

### 2.9 Molecular Docking

**FIGURE 4:**
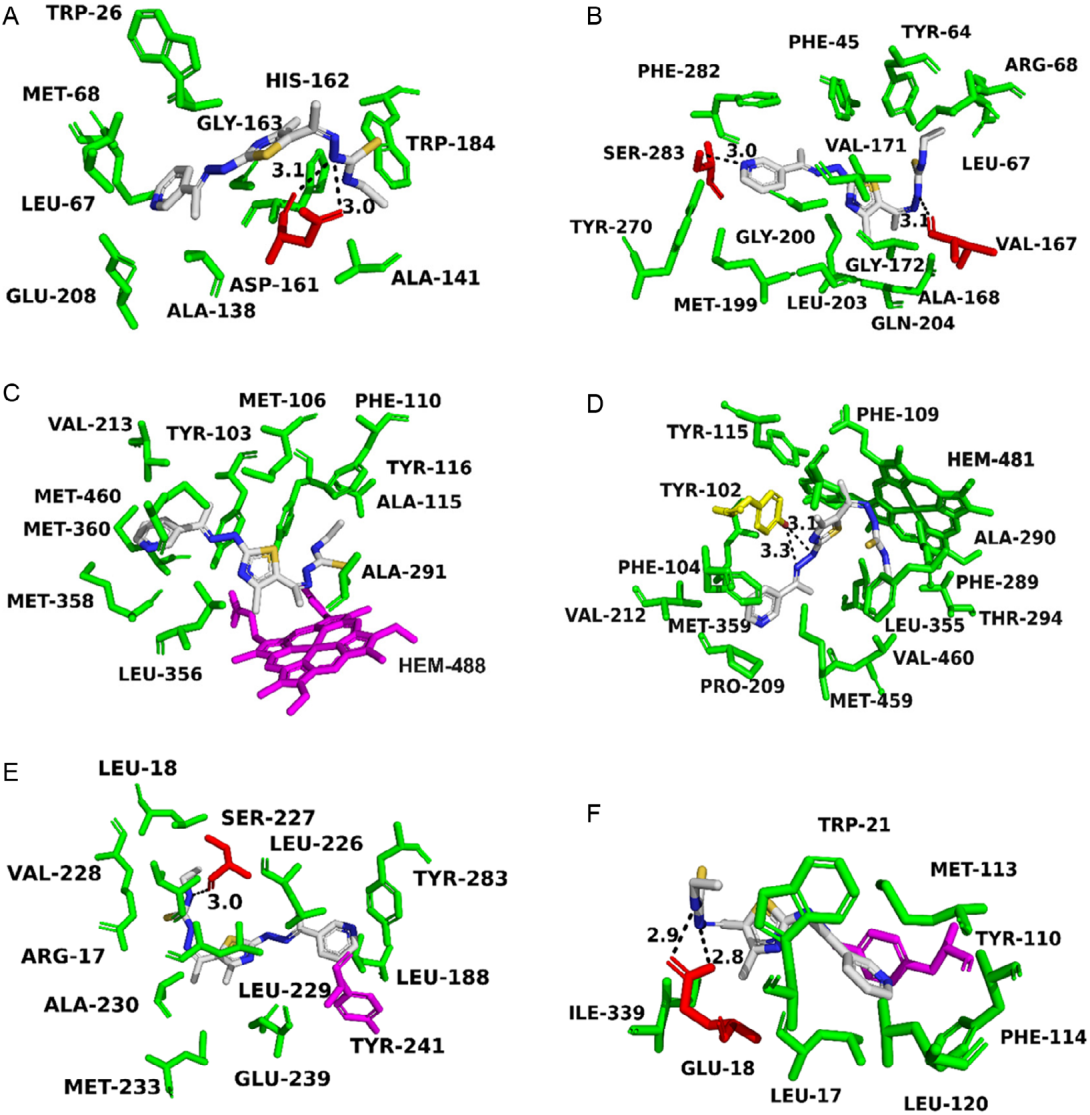
Molecular docking of Compound 8 and targets for Leishmaniasis and chagas disease. (A) *T. cruzi* cruzain residues that make hydrophobic contacts (green) and hydrogen bonds (red) with Compound 8. (B) *T. cruzi* squalene synthase residues that make hydrophobic contacts (green) and hydrogen bonds (red) with 8. (C) *T. cruzi* 14‐alpha‐demethylase residues that make hydrophobic contacts (green) and T‐Stacking (magenta) with Compound 8. (D) *L. infantum* Sterol 14‐demethylase residues that make hydrophobic contacts (green), hydrogen bonds (red), and Pi‐stacking (yellow) with Compound 8. (E) *L. infantum* pteridine reductase residues that make hydrophobic contacts (green), hydrogen bonds (red), and T‐stacking (magenta) with Compound 8. (F) *L. infantum* trypanothione reductase residues that make hydrophobic contacts (green), hydrogen bonds (red), and T‐stacking (magenta) with Compound 8.

A molecular docking study was conducted to elucidate the potential interactions of Compound 8 with squalene synthase, sterol 14α‐demethylase, pteridine reductase, and trypanothione reductase, which are key molecular targets in *L.* *spp*. and *T. cruzi*. Figure 4 presents a detailed analysis of the predicted binding modes of Compound 8 with each target.

Compound 8 was predicted to bind to cruzain (Figure 4A) through hydrophobic interactions with residues ALA138, ALA141, GLU208, GLY163, HIS162, LEU67, MET68, TRP184, and TRP26, as well as two hydrogen bonds (3.0 and 3.1 Å) with ASP161, resulting in a docking score of 51.00. The active site of cruzain contains structural elements that are highly conserved among cysteine proteases, including the catalytic triad composed of CYS25, HIS162, and ASP161 [50].

For squalene synthase (Figure 4B), Compound 8 established hydrophobic contacts with residues GLY172, MET199, GLY200, VAL171, ALA168, LEU203, PHE45, TYR64, ARG68, LEU67, PHE282, TYR270, and GLN204, together with two hydrogen bonds involving SER283 (3.0 Å) and VAL167 (3.1 Å), yielding a docking score of 69.16. In contrast, docking against sterol 14α‐demethylase (Figure 4C) produced a higher docking score of 75.81. In this complex, Compound 8 exhibited a T‐shaped π‐stacking interaction with the heme cofactor (HEM488) and hydrophobic contacts with residues TYR103, VAL213, MET106, MET460, LEU356, ALA291, MET358, PHE110, ALA115, TYR116, and MET360. Among the T. *cruzi* targets evaluated, sterol 14α‐demethylase (CYP51) displayed the highest predicted binding affinity for Compound 8. The T‐shaped π‐stacking interaction with the heme cofactor, HEM488, appears to contribute to the energetic stabilization of the protein–ligand complex [51].

Regarding the *Leishmania* targets, Figure 4D illustrates the interactions between Compound 8 and *L. infantum* sterol 14α‐demethylase (CYP51). The ligand established hydrophobic contacts with residues ALA290, LEU355, MET359, MET459, PHE104, PHE109, PHE289, PRO209, THR294, TYR115, VAL212, VAL460, and the heme group (HEM481), in addition to two hydrogen bonds (3.1 and 3.3 Å) and one π‐stacking interaction with TYR102, resulting in a docking score of 65.34. Among the *Leishmania* targets investigated, CYP51 exhibited the highest predicted affinity for Compound 8. This observation is likely attributable to the large number of hydrophobic contacts and the π‐stacking interaction with TYR102, a residue recognized as critical for ligand binding in previous studies [52].

The interaction between Compound 8 and pteridine reductase (Figure 4E) yielded a docking score of 64.55. The predicted binding mode involved hydrophobic contacts with residues ALA230, ARG17, GLU239, LEU18, LEU188, LEU226, LEU229, MET233, TYR283, and VAL228, along with a hydrogen bond with SER227 (3.0 Å) and a T‐shaped π‐stacking interaction with TYR241. Notably, Compound 8 interacted with residues that form the enzyme's hydrophobic pocket (LEU188, LEU226, and LEU229), which likely contributes to the stabilization of the predicted complex [53].

Finally, Figure 4F depicts the interactions between Compound 8 and trypanothione reductase, which resulted in a docking score of 58.00. The predicted binding mode was characterized by hydrophobic contacts with residues ILE339, LEU120, LEU17, MET113, PHE114, and TRP21, hydrogen bonds with GLU18 (2.8 and 2.9 Å), and a T‐shaped π‐stacking interaction with TYR110. Although the docking protocol was validated by redocking the cocrystallized ligands, molecular docking provides only an estimate of binding affinity. Therefore, the proposed binding modes should be regarded as predictive, and experimental enzyme inhibition assays are required to confirm the inhibitory activity of Compound 8.

### 2.10 Physicochemical Property Profile and ADME Parameters New 3 and 4 Pyridyl‐Thiazole‐Thiosemicarbazones Derivatives

SwissADME [54] analysis of the synthesized thiazoles indicated that these molecules comply with both Lipinski's rule of five [43] and Veber's criteria [55], as summarized in Table 5. The compounds are small in size, possess a limited number of hydrogen bond donors and acceptors, display low polar surface area, and exhibit favorable partition coefficients. Collectively, these properties suggest that the compounds possess suitable characteristics for the development of orally administered drugs.

**TABLE 5:** Analysis of the Lipinsk and Veber rule criteria of synthesized thiazoles, data obtained from SwissADME (http://swissadme.ch/).

Compound	H_A_ a ≤ 10	H_D_ b ≤ 5	M log*P* c ≤ 4.15	Molecular mass ≤ 500	Rotatable links ≤ 10	PSA^d^ < 140 Å^2^	GI absorption	BBB^e^	Bio
**1**	4	1	2.52	274.34	3	98.71	High	No	0.55
**2**	4	0	2.49	288.37	3	87.85	High	No	0.55
**3**	4	0	3.02	302.39	4	87.85	High	No	0.55
**4**	4	1	3.13	274.34	3	98.71	High	No	0.85
**5**	4	0	3.13	350.44	4	87.85	High	Yes	0.85
**6**	4	3	2.56	347.46	5	164.14	High	No	0.55
**7**	4	3	2.92	361.47	6	150.15	High	No	0.55
**8**	4	3	3.42	375.51	7	150.15	High	No	0.55
**9**	4	3	3.70	423.56	7	150.15	Low	No	0.55
**10**	4	2	3.05	361.49	5	153.28	Low	No	0.55
**11**	4	2	3.73	389.54	7	139.29	High	No	0.55
**12**	4	2	3.53	375.51	6	153.28	Low	No	0.55
**13**	4	3	3.13	349.48	5	157.44	High	Yes	0.85
**14**	4	3	3.13	361.49	6	150.15	High	Yes	0.85
**15**	4	3	3.13	375.51	7	150.15	High	Yes	0.85
**16**	4	3	3.13	423.56	7	150.15	High	Yes	0.85
**17**	4	2	3.3	423.56	6	153.28	High	Yes	0.85
**18** **19**	4 4	2 2	3.13 2.36	437.58 451.61	7 8	139.39 139.29	High Low	Yes No	0.85 0.55
**20**	4	2	3.13	499.65	8	139.29	High	Yes	0.85

a Number of hydrogen bond acceptors.

b Number of hydrogen bond donors.

c Parttition coefficient.

d Polar surface area.

e Blood brain barrier.

Additionally, SwissADME provides predictions of gastrointestinal absorption and blood–brain barrier (BBB) permeation by defining favorable and unfavorable zones for passive diffusion across these physiological barriers. According to the BOILED‐Egg (Brain or Intestinal Estimated Diffusion) model, the synthesized thiazoles are predicted to exhibit high gastrointestinal absorption while lacking BBB permeability, as shown in Table 5.

For the 3‐ and 4‐pyridyl–thiazole–thiosemicarbazone derivatives, it can be observed that compounds 6–10 and 13–17 exhibit polar surface area (PSA) values above the recommended threshold.

## 3 Conclusion

In this study, a series of 3‐ and 4‐pyridyl‐1,3‐thiazoles bearing a thiosemicarbazone moiety was rationally designed, synthesized, and evaluated for activity against Trypanosomatidae parasites. Intermediates **1–5** and pyridyl‐thiazole‐thiosemicarbazone derivatives **6–20** were assessed for cytotoxicity as well as anti‐*T. cruzi* and leishmanicidal activities. Among the compounds tested against *T. cruzi*, Compound 8 exhibited the most favorable in vitro profile, with an IC_50_ of 1.0 µM and a selectivity index (SI) of 52.8 against the amastigote and trypomastigote forms of the Tulahuen strain, along with an LC_50_ of 0.3 µM against the trypomastigote form of the Y strain. However, despite this potent in vitro activity, Compound **8** displayed no measurable in vivo efficacy when administered orally to mice at 150 mg/kg for five consecutive days, whereas benznidazole treatment resulted in 100% survival and undetectable parasitemia. This in vitro/in vivo disconnect likely reflects underlying pharmacokinetic or ADME liabilities such as poor oral bioavailability, rapid metabolic clearance, or inadequate tissue penetration that were not assessed prior to the efficacy study. Compound **8** also demonstrated notable in vitro activity against *L. amazonensis* amastigotes (IC_50_ = 3.9 µM, SI = 32.0) and showed the best performance among the tested compounds against *L. infantum* promastigotes (IC_50_ = 8.8 µM, SI = 14.2). In silico predictions of physicochemical and ADMET properties indicated that all synthesized compounds exhibit favorable bioavailability scores, drug‐likeness, and chemical stability based on computational models; however, these predictions were not validated experimentally. Overall, these preliminary findings demonstrate that the pyridyl‐thiazole‐thiosemicarbazone scaffold can generate compounds with selective in vitro antiparasitic activity. Nevertheless, the lack of in vivo efficacy for Compound **8** underscores critical translational challenges that must be addressed before this chemotype can be considered therapeutically viable. Future work will prioritize comprehensive DMPK/PK profiling, including oral bioavailability, tissue distribution, metabolic stability, and plasma protein binding, to identify and overcome exposure‐related barriers. Depending on these results, studies to improve metabolic stability and solubility, advanced formulation strategies (e.g., nanoparticle encapsulation or prodrug design), or alternative dosing regimens may be pursued.

## 4 Materials and Methods

### 4.1 Chemistry

All reagents and solvents were of analytical grade (Sigma‐Aldrich). Melting points were determined uncorrected on a Fisaton 430D apparatus. Reaction progress was monitored by TLC on silica gel 60 F_254_ precoated aluminum sheets (Merck). ^1^H‐ and ^13^C‐NMR spectra were recorded on Varian Unity Plus (400 MHz) or Bruker AMX (300 MHz) spectrometers.

### 4.2 General Procedures for the Synthesis of Thiosemicarbazones

In the first step, thiosemicarbazide (8 mmol) was dissolved in 30 mL of isopropyl alcohol in a round‐bottom flask, followed by the addition of 3‐acetylpyridine (8 mmol). Three drops of sulfuric acid were then added, and the reaction mixture was refluxed at 83 °C for 3 h. The progress of the reaction was monitored by TLC using a hexane/ethyl acetate (7:3) solvent system. Upon completion, the resulting precipitate was collected by vacuum filtration, washed with cold isopropyl alcohol, and purified by recrystallization from a toluene/ethyl acetate mixture (70:30). The purified solid was dried in a desiccator and stored for subsequent use.

### 4.3 Synthesis of Intermediate Compounds 1–5

In a 125 mL round‐bottom flask, thiosemicarbazone (8 mmol) was dissolved in 20 mL of isopropyl alcohol, followed by the addition of 3‐chloro‐2,4‐pentanedione (8 mmol). Five drops of sulfuric acid were then added, and the reaction mixture was refluxed at 83 °C for 3 h. Reaction progress was monitored by TLC using a hexane/ethyl acetate (7:3) solvent system. After completion of the reaction, the resulting precipitate was collected by vacuum filtration, washed with cold isopropyl alcohol, and purified by recrystallization from a toluene/ethyl acetate mixture (70:30). The purified solid was dried in a desiccator and stored for further use.

#### 4.3.1 ‐(4‐Methyl‐2‐(2‐(1‐(pyridin‐3‐yl)ethylidene)hydrazineylidene)‐2,3‐dihydrothiazol‐5‐yl)ethan‐1‐one (1)

Yellow crystals, 93% yield, melting point 218 °C. 1H NMR (DMSO‐400 MHz‐d6) *δ* 2.35 (s, 3H, CH3), 2.39 (s, 3H, CH3), 2.48 (s, 3H, CH3), 7.93 (t,1H, pyridine), 8.35 (d, 1H, pyridine), 9.08 (d, 1H, pyridine), 9.42 (s, 1H, pyridine), 10.56 (s, 1H, N—H, thiazole). 13C NMR (400 MHz, DMSO‐d6) *δ* 13.625 (CH3), 16.630 (CH3), 29.27 (CH3), 120.23 (C5, thiazole), 126.28–141.49 (pyridine), 142.82 (S—C=N), 147.61 (C4‐thiazole), 169.51 (CH3—C=N), 188.95 (CH3—C=O).

#### 4.3.2 1‐(3,4‐Dimethyl‐2‐(2‐(1‐(pyridin‐3‐yl)ethylidene)hydrazineylidene)‐2,3‐dihydrothiazol‐5‐yl)ethan‐1‐one (2)

Yellow crystals, yield 89%, melting point 240 °C. 1H NMR (DMSO‐400 MHz‐d6) *δ* 2.36 (s, 3H, CH3), 2.40 (s, 3H, CH3), 3.0 (s, 3H, CH3), 3.53 (s, 3H, CH3), 8.1 (t, 1H, pyridine), 8.85 (d, 1H, pyridine), 9.1 (d, 1H, pyridine), 9.42 (s, 1H, pyridine). 13C NMR (400 MHz, DMSO‐d6) *δ* 13.51 (s, 3H, CH3), 13.87 (s, 3H, CH3), 29.66 (s, 3H, CH3), 31.91 (s, 3H, CH3), 126.34 (C—S, thiazole), 136.47–141.93 (C—H, pyridine), 161.97 (C—CH3, thiazole), 168.31 (C–N, thiazole), 176 (C=N), 186.72 (C=O).

#### 4.3.3 1‐(3‐Ethyl‐4‐methyl‐2‐(2‐(1‐(pyridin‐3‐yl)ethylidene)hydrazineylidene)‐2,3‐dihydrothiazol‐5‐yl)ethan‐1‐one (3)

Yellow crystals, yield 87%, melting point 251 °C. 1H NMR (DMSO‐400 MHz‐d6) *δ* ppm 1.28 (t, 3H, CH2‐CH3), 2.41(s, 3H, CH3), 2.46 (s, 3H, CH3), 2.60 (s, 3H, CH3), 4.09 (q, 2H, CH2‐ N, thiazole), 8.03–9.1 (4H, pyridine). 13C NMR (400 MHz, DMSO‐d6) *δ* 12.89 (CH3—C=C, thiazole), 13.91 (CH3‐CH2, thiazole), (CH3—C=N) 29.7 (CH3—C=O), 39.09 (CH2‐CH3, thiazole), 115.26 (C=C‐S, thiazole), 126.53−142.45 (H, pyridine), 152.15 (C—N, thiazole), 167.32 (C=N), 188.80 (C=O).

#### 4.3.4 1‐(2‐(2‐(1‐(Pyridin‐4‐yl)ethylidene)hydrazineyl)thiazol‐5‐yl)ethan‐1‐one (4)

Yellow crystals, yield 83%, melting point 285 °C. 1H NMR (DMSO‐400 MHz‐d6) *δ* ppm 8.84 (d, 2H, heterocyclic), 8.21 (d, 2H, heterocyclic), 2.52 (s, 2H, CH2), 2.49 (s, 3H, CH3), 2.39 (s, H, CH3), 1.22 (s, H, CH3). 13C NMR (400 MHz, DMSO‐d6) *δ* 199.11 (C=O), 170.44 (N‐C‐S), 151.97 (C,heterocyclic), 142.44 (C=N), 122.21 (C=S), 38.87 (C=N, heterocyclic), 29.26 (CH,heterocyclic), 16.40 (CH3), 13.40 (CH3).

#### 4.3.5 1‐(3‐Phenyl‐2‐(2‐(1‐(pyridin‐4‐yl)ethylidene)hydrazineylidene)‐2,3‐dihydrothiazol‐5‐yl)ethan‐1‐one (5)

Yellow crystals, yield 83%, melting point 287 °C. 1H NMR (DMSO‐400 MHz‐d6) *δ* 8.85 (d, 2H, heterocyclic), 8.26(d, 2H, heterocyclic), 7.64 (m, 5H, Ar), 4.58 (s, H, thiazole), 2.26 (s, CH3), 2.13 (s, CH3). 13C NMR (400 MHz, DMSO‐d6) *δ* 177.37 (C=O), 170.97 (N=C—S), 152.81 (CH, pyridine), 150.90 (C‐Ar), 143.70 (C=N, thiazole), 141.92 (C, heterocyclic), 136.87 (CH‐Ar), 129.75 (CH‐Ar), 128.49 (CH, heterocyclic), 122.17 (CH‐Ar), 115.57 (C‐S, thiazole), 14.20 (CH3), 13.26 (CH3).

### 4.4 Synthesis of Pyridyl‐Thiazole‐*Thiosemicarbazones (6–20)

In a 125 mL round‐bottom flask, intermediate 1–5 (8 mmol) was dissolved in 15 mL of isopropyl alcohol, followed by the addition of thiosemicarbazide (8 mmol) and three drops of sulfuric acid. The reaction mixture was refluxed at 83 °C for 3 h. Reaction progress was monitored by TLC using a hexane/ethyl acetate (7:3) solvent system. Upon completion, the resulting precipitate was collected by vacuum filtration, washed with cold isopropyl alcohol, and purified by recrystallization from a toluene/ethyl acetate mixture (70:30). The purified solid was dried in a desiccator.

#### 4.4.1 2‐(1‐(4‐Methyl‐2‐(2‐(1‐(pyridin‐3‐yl)ethylidene)hydrazineylidene)‐2,3‐dihydrothiazol‐5‐yl)ethylidene)hydrazine‐1‐carbothioamide (6)

Orange‐yellow crystals, 93% yield, melting point 225 °C. 1H NMR (DMSO‐400 MHz‐d6) *δ* 2.28 (s, 3H, CH3), 2.38 (s, 3H, CH3), 2.39 (s, 3H, CH3), 7.31 (s, 1H, NH‐C‐S), 7.58 (m, 2H, pyridine), 8.74 (m, 1H, pyridine), 9.1 (s,1H, N‐H, thiazole), 10.36 (s,1H, NH‐N). 13C NMR (400 MHz, DMSO‐d6) *δ* 13.78 (CH3), 15.95 (CH3) e 16.19 ppm (CH3), 136.81–164.06 (C, pyridine), 168.11 (C=N), 178,26 (C=S).

#### 4.4.2 N‐Methyl‐2‐(1‐(4‐methyl‐2‐(2‐(1‐(pyridin‐3‐yl)ethylidene)hydrazineylidene)‐2,3‐dihydrothiazol‐5‐yl)ethylidene)hydrazine‐1‐carbothioamide (7)

Crystals Dark yellow, yield 83%, melting point 229.5 °C. 1H NMR (DMSO‐400 MHz‐d6) *δ* 2.28 (s, 3H, CH3), 2.39 (s, 3H, CH3), 2.50 (s, CH3), 3.03 (s, 3H, CH3), 7.86 (d, 1H, pyridine), 8.1 (q, 1H, NH‐CH3), 8.87 (t, 1H, pyridine), 9.14 (s,1H, pyridine), 9.46 (d,1H, pyridine), 10.31 (s, 1H, N‐H, thiazole), 10.37 (s, 1H, NH). 13C NMR (400 MHz, DMSO‐d6) *δ* 13.76 (CH3), 15.94 (CH3), 16.31(CH3), 31.14 (CH3), 117.43 (C‐S, thiazole), 126.74−141.48 (5C, pyridine), 141.97 (C—N, thiazole), 144.93 (C=N), 145.93 (S—C—N, thiazole), 168.01 (CH3‐C=N), 178.23 (C=S).

#### 4.4.3 N‐Ethyl‐2‐(1‐(4‐methyl‐2‐(2‐((1‐(pyridin‐3‐yl)ethylidene)hydrazineylidene)‐2,3‐dihydrothiazol‐5‐yl)ethylidene)hydrazine‐1‐carbothioamide (8)

Orange‐yellow crystals, yield 82%, melting point 231 °C. 1H NMR (DMSO‐400 MHz‐d6) *δ* 1.13 (t, 3H, CH3), 2.28 (s, 3H, CH3), 2.42 (s, 3H, CH3), 2.49 (s, 3H, CH3), 3.75 (q, 2H, CH2), 7.86 (t,1H, NH), 8.8 (t, 1H, pyridine), 9.1 (d, 1H, pyridine), 9.4 (d, 1H, pyridine), 9.6 (s, 1H, pyridine), 10.2 (s, 1H, thiazole), 10.5 (s, 1H, NH). 13C NMR (400 MHz, DMSO‐d6) *δ* 13.72 (CH3), 13.84 (CH3), 14.49 (CH3), 16.5 (CH3), 40.13 (CH2), 98.4 (C—S, thiazole), 136.7 – 141.4 (C—H, pyridine), 147.4 (C=C, thiazole), 167.8 (C=N), 170.2 (C=N, thiazole), 177.29 (C‐CH3), 179.9 (C=S).

#### 4.4.4 2‐(1‐(4‐Methyl‐2‐(2‐(1‐(pyridin‐3‐yl)ethylidene)hydrazineylidene)‐2,3‐dihydrothiazol‐5‐yl)ethylidene)‐N‐phenylhydrazine‐1‐carbothioamide (9)

Dark yellow crystals, yield 79%, melting point 279 °C. 1H NMR (DMSO‐400 MHz‐d6) *δ* 2.38 (s, 3H, CH3), 2.44 (s, 3H, CH3), 2.52 (s, 3H, CH3), 7.14–7.67 (5H, benzene), 8.8–9.4 (4H, pyridine), 10.59 (s, 1H, N‐H, thiazole), 10.8 (s, 2H, N‐H). 13C NMR (400 MHz, DMSO‐d6) *δ* 13.63 (CH3), 16.32 (CH3), 18.46 (CH3), 117.35 (C‐S, thiazole), 124.12−132.29 (6 C, aromatic), 136.2−141.7 (5C, pyridine), 142.5 (C=C, thiazole), 146.23 (C=N), 167.24 (C=N), 176 (C=C), 179.3 (C=S).

#### 4.4.5 2‐(1‐(3,4‐Dimethyl‐2‐(2‐(1‐(pyridin‐3‐yl)ethylidene)hydrazineylidene)‐2,3‐dihydrothiazol‐5‐yl)ethylidene)hydrazine‐1‐carbothioamide (10)

Yellow crystals, 93% yield, melting point 241 °C. 1H NMR (DMSO‐400 MHz‐d6) *δ* 2.28 (s, 3H, CH3), 2.41 (s, 3H, CH3), 2.44 (s, 3H, CH3), 3.50 (s, 3H, CH3, thiazole), 7.58 (t, 1H, pyridine), 7.99 (s, 1H, NH2), 8.27 (d, 1H, pyridine), 8.80 (d, 1H, pyridine), 9.13 (s, 1H, pyridine), 10.13 (s, 1H, NH‐N). 13C NMR (400 MHz, DMSO‐d6) *δ* 13.51 (CH3), 13.68 (CH3), 16.93 (CH3) 31.76 (CH3), 113.49 (C‐S, thiazole), 126.45–140.48 (C, pyridine), 142.0 (C—N, thiazole), 144.4 (C=N), 144.42 (C—S, thiazole), 150.03 (C=N), 168.66 (C=N), 178.36 (C=S).

#### 4.4.6 2‐(1‐(3,4‐Dimethyl‐2‐(2‐(1‐(pyridin‐3‐yl)ethylidene)hydrazineylidene)‐2,3‐dihydrothiazol‐5‐yl)ethylidene)‐N‐ethylhydrazine‐1‐carbothioamide (11)

Dark yellow crystals, yield 73%, melting point 243 °C. 1H NMR (DMSO‐400 MHz‐d6) *δ* 1.45 (t, 3H, CH3), 2.22 (s, 3H, CH3), 2.41 (s, 3H, CH3), 3.10 (s, 3H, CH3), 3.98 (s, 3H, CH3), 4.7 (q, 2H, CH2), 7.67 (s, 1H, N‐H), 7.9 (t, 1H, pyridine), 8.8 (d, 1H, pyridine), 9.0 (d, 1H, pyridine), 9.2 (s, 1H, pyridine), 10.5 (s,1H, N‐H). RMN 13C (DMSO‐d6, 400 MHz) *δ* ppm 11.3 (CH3), 12.4 (CH3), 14.0 (CH3), 15.8 (CH3), 34.0 (CH3), 44.0 (CH2), 101.1(C=C), 124.8−160.3 (C, pyridine), 160.5 (C=C), 163.4 (C=N), 14.6 (C=N), 165.6 (C=N), 180.1 (C=S).

#### 4.4.7 2‐(1‐(3‐Ethyl‐4‐methyl‐2‐(2‐(1‐(pyridin‐3‐yl)ethylidene)hydrazineylidene)‐2,3‐dihydrothiazol‐5‐yl)ethylidene)hydrazine‐1‐carbothioamide (12)

Dark yellow crystals, yield 88%, melting point 276 °C. 1H NMR (DMSO‐400 MHz‐d6) *δ* 1.28 (t, 3H, CH3), 2.28 (s, 3H, CH3), 2.37 (s, 3H, CH3), 2.9 (s, 3H, CH3), 4.06 (q, 2H, CH2), 8.0 (t, 1H, pyridine), 8.8 (d, 1H, pyridine), 8.9 (d, 1H, pyridine), 8.9 (t, 1H, pyridine) 9.1 (s, 1H, pyridine), 10.2 (s,1H, NH), 10.3 (s, 1H, NH). 13C NMR (400 MHz, DMSO‐d6) *δ* 12.89 (CH3), 13.51 (CH3), 13.67 (CH3), 16.97(CH3), 29.72 (CH2), 113.1 (C‐S, thiazole), 126.2 −149.3 (CH, pyridine), 152.2 (C=C, thiazole), 167.3 (C=N), 167.9 (S‐C–N), 178.0 (C=N), 188.9 (C=S).10.2 (s,1H, NH), 10.3 (s, 1H, NH).

#### 4.4.8 2‐(1‐(2‐(2‐(1‐(pyridin‐4‐yl)ethylidene)hydrazineyl)thiazol‐5‐yl)ethylidene)hydrazine‐1‐carbothioamide (13)

Dark yellow crystals, yield 65.54%, melting point 224 °C. 1H NMR (DMSO‐400 MHz‐d6) *δ* 10.37 (s, 2H, NH2), 8.79 (d, 2H, heterocyclic), 8.23, (d, 2H, heterocyclic), 7.26 (s, 1H, NH), 5.29 (s, H, NH), 3.40 (t, 2H, NH2), 2.43 (s, 2H, CH2‐thiazole), 2.39 (s, 3H, CH3), 2.29 (s, 3H, CH3). 13C NMR (400 MHz, DMSO‐d6) *δ* 178.30 (C=S), 169.41 (N=C‐S), 152.07 (C=N), 145.95 (C=N, exocyclic), 122 (C,thiazole), 117.91 (CH, thiazole), 144.88 (C–C=C, heterocyclic), 141.89 (CH, heterocyclic), 141.14 (CH, heterocyclic), 16.25 (CH3), 15.84 (CH3).

#### 4.4.9 N‐Methyl‐2‐(1‐(2‐(2‐(1‐(pyridin‐4‐yl)ethylidene)hydrazineyl)thiazol‐5‐yl)ethylidene)hydrazine‐1‐carbothioamide (14)

Yellow crystals, yield 78%, melting point 214 °C. 1H NMR (DMSO‐400 MHz‐d6) *δ* 10.32 (s, 2H, NH2), 8.80 (d, 2H, heterocyclic), 8.24 (d, 2H, heterocyclic), 7.89 (s, H, thiazole), 5.31 (s, H, NH), 2.39 (d, 3H, CH3), 2.36 (s, 3H, CH3), 2.28 (s, 3H, CH3). 13C NMR (400 MHz, DMSO‐d6) *δ* 178.24 (C=S), 169.49 (N=C‐S),152.93 (C=N), 145.76 (C=N), 144.70 (CH, heterocyclic), 141.741 (CH, heterocyclic), 140.92 (C=C‐S), 122.05 (=C‐thiazole), 117.99 (=C‐thiazole), 31.17 (CH3), 16.34 (CH3), 15.80 (CH3).

#### 4.4.10 N‐Phenyl‐2‐(1‐(2‐(2‐(1‐(pyridin‐4‐yl)ethylidene)hydrazineyl)thiazol‐5‐yl)ethylidene)hydrazine‐1‐carbothioamide (15)

Yellow crystals, yield 67%, melting point 220 °C. 1H NMR (DMSO‐400 MHz‐d6) *δ* 10.30 (s, 1H, NH), 8.80 (d, 2H, heterocyclic), 8.23 (d, 2H, heterocyclic), 7.86 (t, H, Ar), 6.12 (s, 1H, NH), 3.55 (m, 2H, CH2), 2.34 (s, 3H, CH3), 2.28(s, 3H, CH3) 1.14 (t, 3H, CH3). 13C NMR (400 MHz, DMSO‐d6) *δ* 177.22 (C=S), 169.37 (C, thiazole), 152.86 (C=N), 145.73 (C=N), 144.51 (CH, heterocyclic), 142.37 (C, thiazole), 122.31 (CH, heterocyclic), 117.99 (CH, thiazole), 16.41 (CH2), 15.83 (CH3), 14.40 (CH3), 13.18 (CH3).

#### 4.4.11 N‐Phenyl‐2‐(1‐(2‐(2‐(1‐(pyridin‐4‐yl)ethylidene)hydrazineyl)thiazol‐5‐yl)ethylidene)hydrazine‐1‐carbothioamide (16)

Orange crystals, yield 93%, melting point 200 °C, 1H NMR (DMSO‐400 MHz‐d6) *δ* 10.78 (s,1H, NH), 9.60 (s, 1H, NH), 8.83 (s,1H, NH), 8.84 (s, 1H, NH), 8.77 (d, 2H, heterocyclic), 8.20 (d, 2H, heterocyclic), 7.14 (m, 6H, Ar), 5.12 (s, 1H, NH), 1.06 (s, 3H, CH3), 1.03 (s, 3H, CH3). 13C NMR (400 MHz, DMSO‐d6) *δ* 189.15 (C=S), 175.87 (C, thiazole), 170.50 (C=N), 152.71 (C=N), 145.99 (CH, heterocyclic), 142.50 (C, heterocyclic), 138.92 (C, Ar), 128.26 (CH, Ar), 124.19 (CH, Ar), 122.27 (CH, heterocyclic), 117.78 (CH, thiazole), 16,11 (CH3), 13.26 (CH3).

#### 4.4.12 2‐(1‐(3‐Phenyl‐2‐(2‐(1‐(pyridin‐4‐yl)ethylidene)hydrazineylidene)‐2,3‐dihydrothiazol‐5‐yl)ethylidene)hydrazine‐1‐carbothioamide (17)

Orange crystals, yield 83%, melting point 287 °C, 1H NMR (DMSO‐400 MHz‐d6) *δ* 10.484 (s, H, NH), 8.80 (d, 2H, pyridine), 8,33 (s, 2H, NH2), 8.22 (d, 2H, pyridine), 7.62 (m, 5H, Ar), 7.36 (s, H, thiazole), 2.33 (s, CH3), 2.12 (s, CH3). 13C NMR (400 MHz, DMSO‐d6) *δ* 178.51 (C=S), 170.76 (N=C—S, thiazole), 152.62 (C=N), 151.06 (C, pyridine), 144.11 (C, thiazole), 142.12 (=C—N), 137.13 (C, Ar), 136.28 (C, Ar), 129.559 (C, Ar), 129.50 (C, Ar), 128.50 (C, pyridine) 122.12 (C=N), 115.56 (C, thiazole), 16.76 (CH3), 13.26 (CH3).

#### 4.4.13 N‐Methyl‐2‐(1‐(3‐phenyl‐2‐(2‐(1‐(pyridin‐4‐yl)ethylidene)hydrazineylidene)‐2,3‐dihydrothiazol‐5‐yl)ethylidene)hydrazine‐1‐carbothioamide (18)

Orange crystals, yield 20.13%, melting point 198 °C. 1H NMR (DMSO‐400 MHz‐d6) *δ* 10.44 (s, H, NH), 8.81 (d, 2H, heterocyclic), 8.24 (d, 2H, heterocyclic), 8.00 (s, H, thiazole), 7.62 (m, 5H, Ar), 8.21 (s, H, NH), 3.06 (d, 3H, CH3), 2.33 (s, CH3), 2.13 (s, CH3). 13C NMR (400 MHz, DMSO‐d6) *δ* 178.37 (C=S), 170.91 (N=C—S), 152.91 (C=N), 150.91 (C, heterocyclic), 143.86 (C, thiazole), 142.08 (=C—N), 136.85 (C, Ar), 136.29 (C, Ar), 129.56 (C, Ar), 129.27 (C, Ar), 128.48 (C, heterocyclic), 128.48 (C=N), 31.21 (CH3), 14.54 (s, CH3), 13.24 (CH3).

#### 4.4.14 N‐Ethyl‐2‐(1‐(3‐phenyl‐2‐(2‐(1‐(pyridin‐4‐yl)ethylidene)hydrazineylidene)‐2,3‐dihydrothiazol‐5‐yl)ethylidene)hydrazine‐1‐carbothioamide (19)

Orange crystals, yield 32.68%, melting point 191 °C. 1H NMR (DMSO‐400 MHz‐d6) *δ* 10.41 (s, H, NH), 8.79 (d, 2H, heterocyclic), 8.21 (d, 2H heterocyclic), 7.96 (t, 1H, Ar), 7.42 (m, 5H, Ar), 10.43 (s, 1H, NH), 3.71 (m, 2H, CH2), 1.14 (t, 3H, CH3), 1.09 (s, 3H, CH3), 1.00 (s, 3H, CH3). 13C NMR (400 MHz, DMSO‐d6) *δ* 177.37 (C=S), 170.97 (C, thiazole), 152.81 (C=N), 149.73 (C=N), 150.90 (CH, heterocyclic), 143.70 (C, Ar), 141.92 (CH, thiazole), 136.87 (C, heterocyclic), 129.75 (CH, Ar), 129.32 (CH, heterocyclic), 128.49 (CH, Ar), 122.17 (CH, Ar), 115.57 (C, thiazole), 16.86 (CH2), 14.38 (CH3), 14.20 (CH3), 13.26 (CH3).

#### 4.4.15 N‐Phenyl‐2‐(1‐(3‐phenyl‐2‐(2‐(1‐(pyridin‐4‐yl)ethylidene)hydrazineylidene)‐2,3‐dihydrothiazol‐5‐yl)ethylidene)hydrazine‐1‐carbothioamide (20)

Dark yellow crystals, yield 32.63%, melting point 222 °C. 1H NMR (DMSO‐400 MHz‐d6) *δ* 11.29 (s, 1H, NH), 11.32 (s, H, NH), 8.66 (d, 2H, heterocyclic), 7.74 (d, 2H, heterocyclic), 9.35 (s, 1H. thiazole), 7.09 (m, 10H, Ar), 2.95 (s, 3H, CH3), 2.07 (s, 3H, CH3). 13C NMR (400 MHz, DMSO‐d6) *δ* 189.22 (C=S), 170.36 (C, thiazole), 153.07 (C=N), 152.14 (C=N), 145.69 (CH, heterocyclic), 142.59 (C, Ar), 135.71 (CH, thiazole), 129.68 (C, heterocyclic), 129.60 (CH, Ar), 128.87 (C,Ar), 128.50 (C, Ar), 122.37 (C, heterocyclic), 128.37 (C, Ar), 117.00 (C, thiazole), 14.68 (CH3), 13.45 (CH3).

### 4.5 Molecular Docking

In this work, we chose to use three of the same targets employed by our group to study another series of thiazole derivatives [56]. The optimized structure for the ligand was obtained by applying the RM1 semiempirical method at the online platform MolView (molview.org). Protein**–**ligand docking calculations were conducted based on the experimental structures found at the RCSB Protein Data Bank (rcsb.org) for the following *L. infantum* and T. cruzi targets: *L. infantum* Sterol 14‐demethylase (PDB: 3L4D), *L. infantum* Trypanothione Reductase (PDB: 4APN), *L. infantum* Pteridine Reductase (modeled by homology from L. major, PDB: 5L4N), T. cruzi Cruzain (PDB: 3IUT), T. cruzi Squalene Synthase (PDB: 3WCA), and T. cruzi 4‐alpha‐demethylase (PDB: 3K1O). The structure of *L. infantum* Pteridine Reductase (PTR1) was modeled using the SWISS‐MODEL (swissmodel.expasy.org) homology‐modeling tool [57], using a model with 90% of sequence identity. This model was constructed based upon the L. major PTR1 structure as template. GOLD software (Version 5.7.2) [58] was used for docking calculations. The predicted binding mode corresponded to the docking pose with the highest ChemPLP score for each target. The binding site was defined as all atoms within a radius of 7.0 Å around a cocrystallized ligand in each case and redocking validation with RMSD < 2 Å. To better simulate the induced fit phenomena, the following amino acid residues were treated as flexible for each target: (i) Cruzain: CYS25, TRP26, ASP60, SER64, LEU67, MET68, ASN70, MET160, ASP161, and LYS162; (ii) Squalene Synthase: SER44, PHE45, TYR64, VAL167, LEU175, LEU203, GLN204, ASN207, TYR270, and PHE282; (iii) 14‐alpha Demethylase: TYR103, MET106, PHE110, TYR116, PHE290, THR295, LEU356, MET358, MET360, and MET460; (iv) Sterol 14‐demethylase: PHE289, TYR102, VAL460, LEU358, MET105, THR294, CYS422, TYR115, MET459, and PHE104; (v) Pteridine Reductase: ARG17, SER111, PHE113, ASP181, MET183, LEU188, TYR194, LEU226, SER227, and LEU229; (vi) Trypanothione Reductase: CYS52, GLU18, TYR110, MET113, SER109, THR335, ILE339, ILE106, VAL58, and TRP21. The BINANA (durrantlab.pitt.edu/binana) software [59] was used to analyze the intermolecular interactions found in the docking results, using default settings, except for the hydrogen bond distance, set to 3.5 Å. Additionally, PyMOL molecular visualization system was used to generate all the images [60] and to visually inspect the binding pose (only for the best docking score in each target) to confirm interaction distances and orientations for all intermolecular interactions, including hydrogen bonds.

## 5 Biological Assays

### 5.1 Trypanocidal Assay

#### 5.1.1 Test With Tryposmatigotes and Amastigotes Derived from Cell Culture: Tulahuen Strain

The beta‐galactosidase assay developed by Buckner and colleagues (1996) [61] was used, with modifications by Romanha et al. (2010) [26]. This assay uses the Tulahuen strain of *T. cruzi* that expresses the beta‐galactosidase gene from *Escherichia coli*. Briefly, to carry out the tests, mouse fibroblasts from the L929 lineage were seeded in 96‐well plates and incubated for 24 h at 37 °C for adhesion and infection with 10 parasites/cell. After 2 h, the medium containing the extracellular parasites was replaced with new medium and the plate was incubated again at 37 °C for 48 h. After this period, the culture medium was replaced with new medium, in addition to compounds or benznidazole (positive control) at concentrations ranging from 0.78 to 100 µg/mL. After 96 h of incubation, the CPRG (chlorophenol red‐b‐D‐galacto‐pyranoside) substrate was added, the plate was incubated at 37 °C, and the reading was taken after 16–20 h on a spectrophotometer using a 570 nm filter. The results were expressed as the percentage of inhibition of parasite growth. IC_50_ values were calculated by non‐linear regression analysis using GraphPad Prism software. The assays were done in duplicate.

#### 5.1.2 Assay With Trypomastigotes: Y Strain

The trypomastigote forms of Y strain of *T. cruzi* was obtained from the in vitro infection of the L929 cell line, after they reached confluence in cultures maintained in RPMI medium supplemented with 10% FBS and 2 mM Glutamax. To determine the trypanocidal effect, the trypomastigote forms were distributed in 96‐well plates, 100,000 trypomastigotes per well, along with different concentrations of the compounds (1.56 to 200 µg/mL), and incubated for 48 h in an atmosphere of 5% CO_2_, at 37 °C. After this period, the cell viability indicator alamarBlue (Thermo Fisher Scientific) was added at a final concentration of 10%, and the plate was incubated again at 37 °C for 24 h. After this period, the reading was carried out on a spectrophotometer using two wavelengths. As controls, wells with parasites and no treatment, wells with only culture medium and 10% alamarBlue, wells with only culture medium, and wells with parasites treated with the reference drug, benznidazole, were used. Results were expressed as the percentage difference in reduction between untreated and treated parasites using the manufacturer's recommended equation. LC_50_ values were calculated by nonlinear regression analysis using GraphPad Prism software. The assays were done in duplicate.

#### 5.1.3 Cytotoxicity Test on Vertebrate Cells: L929 and RAW 264.7

L929 and RAW 264.7 cells, maintained at 37 °C and 5% CO_2_ in RPMI medium (Invitrogen) supplemented with 10% FBS and 2 mM glutamine, were seeded into 96‐well plates (0.04 × 10^5^ and 0.2 × 10^5^ cells/well, respectively) and incubated for 72 (L929) or 24 h (RAW 264.7) for adhesion and growth. After these periods, the compounds were added at concentrations ranging from 0.78 to 100 µg/mL and the plates were incubated again at 37 °C and 5% CO_2_. Negative controls included untreated cells, and the vehicle toxicity control was 1% DMSO. Following 96 or 48 h of incubation, 20 μL/well of MTT solution (5 mg/mL in PBS) were added, and the microplate was incubed again at 37 ° and 5% CO_2_ for 2 h. Then, cell culture medium was discarded, and 100 µL/well of DMSO was added for formazan crystals solubilization. The absorbance was measured at 570 nm, and the cytotoxicity of the compounds was assessed by comparing treated cultures with the control culture without treatment. The 50% cytotoxic concentration (CC_50_) was calculated by nonlinear regression using the software GraphPad Prism. The assays were done in duplicate.

#### 5.1.4 Tests With *L. amazonensis* and L. Infantum Promastigotes

Promastigote forms of *L. amazonensis* (strain WHOM/00‐LTB0016) and *L. infantum* (strain MHOM/MA/67/ITMAP‐263) that express the beta‐galactosidase gene from *E. coli* [62] were maintained at 26 °C in Schneider's medium (Sigma) supplemented with 10% fetal bovine serum (complete medium) and hemin (2.5 µg/mL). Parasites in the exponential growth phase were used in all experiments. For the leishmanicidal activity assay, the parasites were counted and diluted in complete Schneider's medium (Sigma) to 1 × 10^6^ promastigotes/mL (0.1 × 10^5^ promastigotes per well). The parasites were incubated at 26 °C in the presence of different concentrations of the compounds (0.78 to 100 µg/mL) for 72 h. Parasites incubated with culture medium only and culture medium without parasites containing the compounds were used as controls. After incubation, CPRG solution (500 µM, 0.5% Nonidet P‐40, in PBS) was added, followed by a new incubation for 10 min at 22°C. The absorbance was read at 570 nm on the THERMO SCIENTIFIC Multiskan FC spectrophotometer. The leishmanicidal activity of the compounds was evaluated by the decrease in beta‐galactosidase activity in treated cultures compared to the untreated control culture. Miltefosine was used as a positive control. IC_50_ values were calculated by nonlinear regression analysis using GraphPad Prism software. Each assay was performed in duplicate.

#### 5.1.5 Tests With *L. amazonensis* and L. Infantum Amastigotes

For the amastigote assay, RAW macrophages 264.7 (0.2 × 10^5^ cells/well) were incubated at 37 °C for adhesion at 37 °C with 5% CO_2_. After 1 h, promastigotes forms of *L. amazonensis* or *L. infantum* expressing the *E. coli* beta‐galactosidase gene [62] were added in the proportion of 15 parasites/macrophage for a period of 6h at 37 °C and 5% of CO_2_. The parasites that have not penetrated the cells were discarded by washing, and the culture was incubated again in the presence of different concentrations of the test compounds (0.78 to 100 μg/mL) for 24h, at 37 °C and 5% of CO_2_. Noninfected cells without treatment and infected cells without treatment were used as negative controls and miltefosine was used as the standard. After incubation, the wells were washed, and CPRG solution (500 μM, 0.5% Nonidet P‐40 in PBS) was added, followed by reincubation for 2–6h at 37 °C. The absorbance was measured at 570 nm in the THERMO SCIENTIFIC Multiskan FC spectrophotometer. The leishmanicidal activity of the compounds was assessed by the decrease in beta‐galactosidase activity in the treated cultures in comparison with the control‐infected culture without treatment. Cell growth was assessed and IC_50_ was determined by nonlinear regression analyses using the software GraphPad Prism. Assays were conducted in duplicate.

#### 5.1.6 Assay Validation and Statistical Analysis

Assay validity was assessed using appropriate positive and negative controls. Benznidazole and miltefosine were included as positive controls, while vehicle‐treated cells or parasites (DMSO) served as negative controls. Additional controls included untreated infected cells, untreated noninfected cells, and untreated promastigotes, as appropriate for each assay. Experiments were considered valid when the positive and negative controls produced the expected biological responses, allowing clear discrimination between active and inactive conditions.

IC_50_, CC_50_, and LC_50_ values were determined by nonlinear regression using a four‐parameter logistic (4PL) model implemented in GraphPad Prism version 8.0.1 (GraphPad Software, San Diego, CA, USA). Compound concentrations were log_10_‐transformed prior to curve fitting.

For cytotoxicity assays, cell viability was normalized to untreated cells, which were defined as 100% viability. For antiparasitic assays, untreated infected cells and untreated promastigotes or trypomastigotes (Y strain) were defined as 100% parasite viability. Background signal was determined using untreated noninfected cells and subtracted before data normalization. The resulting normalized values were used for dose–response curve fitting and determination of CC_50_, IC_50_, and LC_50_ values.

Statistical comparisons between each tested compound and the corresponding positive control (miltefosine for *Leishmania* assays and benznidazole for *T. cruzi* assays) were performed using one‐way analysis of variance (one‐way ANOVA) followed by Dunnett's multiple comparisons test.

## Conflicts of Interest

The authors declare no conflicts of interest.

## Supporting information

Supplementary Material

## Data Availability

The data that support the findings of this study are available on request from the corresponding author. The data are not publicly available due to privacy or ethical restrictions.
