# Supplementary material for: Pyridyl‐Thiazole‐Thiosemicarbazones as Antitrypanosomatidae Agents: Design, Synthesis, and In Silico Profiling
Source: ChemMedChem. 2026 Aug 21;21(16):e70443. doi: 10.1002/cmdc.70443 (PMC13494692; doi:10.1002/cmdc.70443)
Supplement: Supplementary file 1 [file CMDC-21-e70443-s001.pdf]

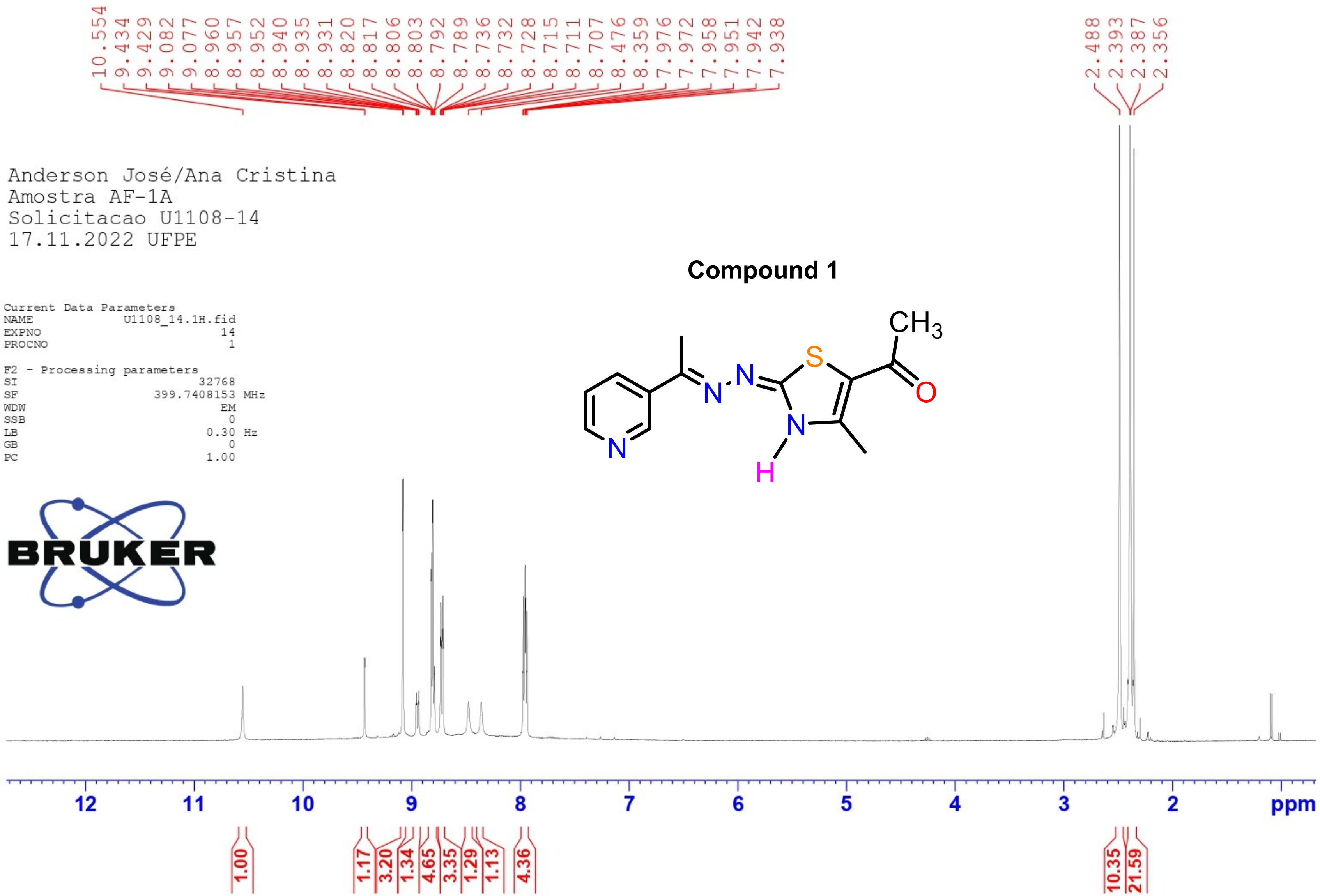

10.554
9.434
9.429
9.082
9.077
8.960
8.957
8.952
8.940
8.935
8.931
8.820
8.817
8.806
8.803
8.792
8.789
8.736
8.732
8.728
8.715
8.711
8.707
8.476
8.359
7.976
7.972
7.958
7.951
7.942
7.938
2.488
2.393
2.387
2.356
Anderson José/Ana Cristina
Amostra AF-1A
Solicitacao U1108-14
17.11.2022 UFPE
Current Data Parameters
NAME U1108_14.1H.fid
EXPNO 14
PROCNO 1
F2 - Processing parameters
SI 32768
SF 399.7408153 MHz
WDW EM
SSB 0
LB 0.30 Hz
GB 0
PC 1.00
Compound 1
CH3
S
N
N
N
O
N
H
12
11
10
9
8
7
6
5
4
3
2
ppm
1.00
1.17
3.20
1.34
4.65
3.35
1.29
1.13
4.36
10.35
21.59

Anderson JosØ/Ana Cristina
Amostra AF-1A
Solicitacao U1108-14
17.11.2022 UFPE

Compound 1

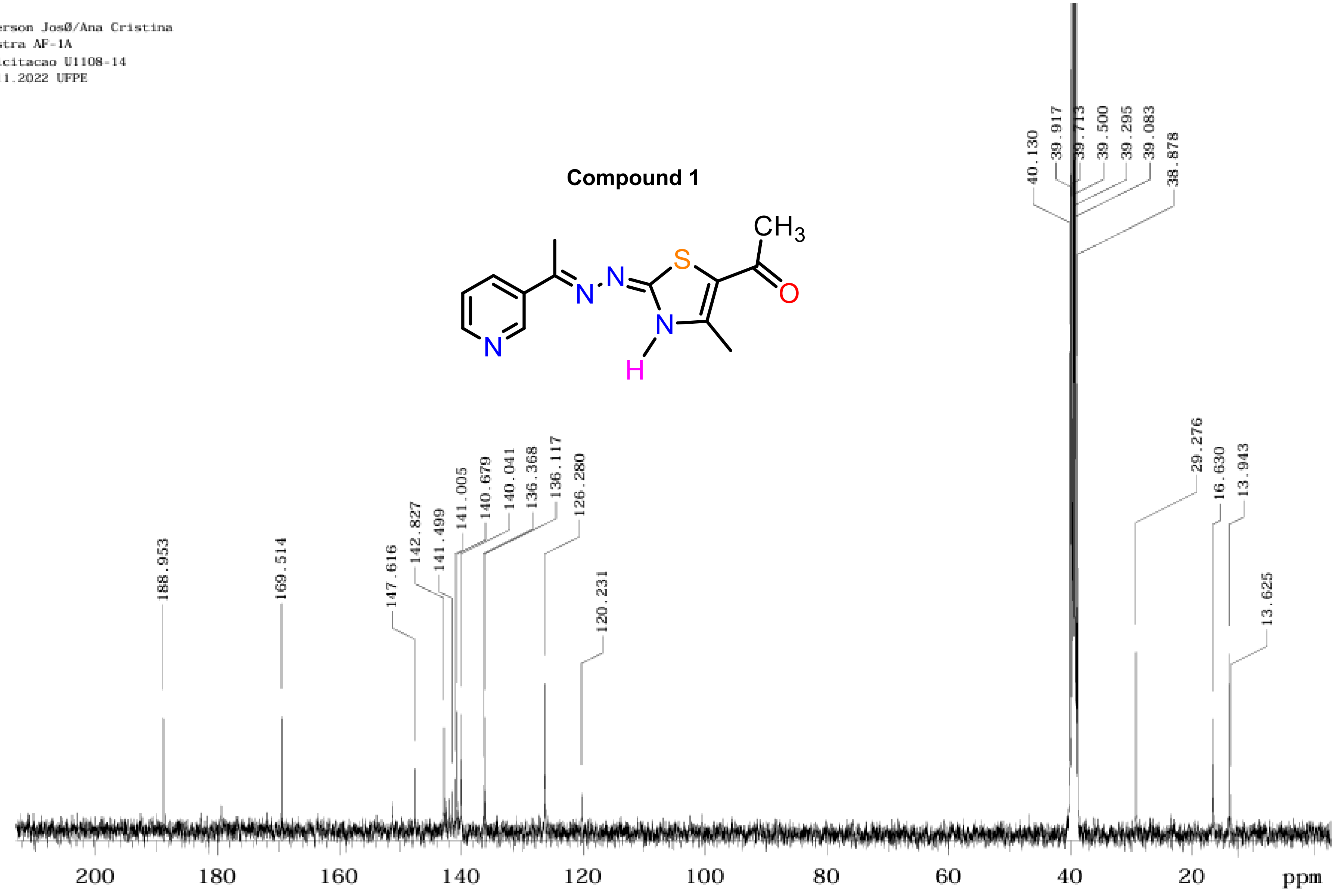

Compound 2

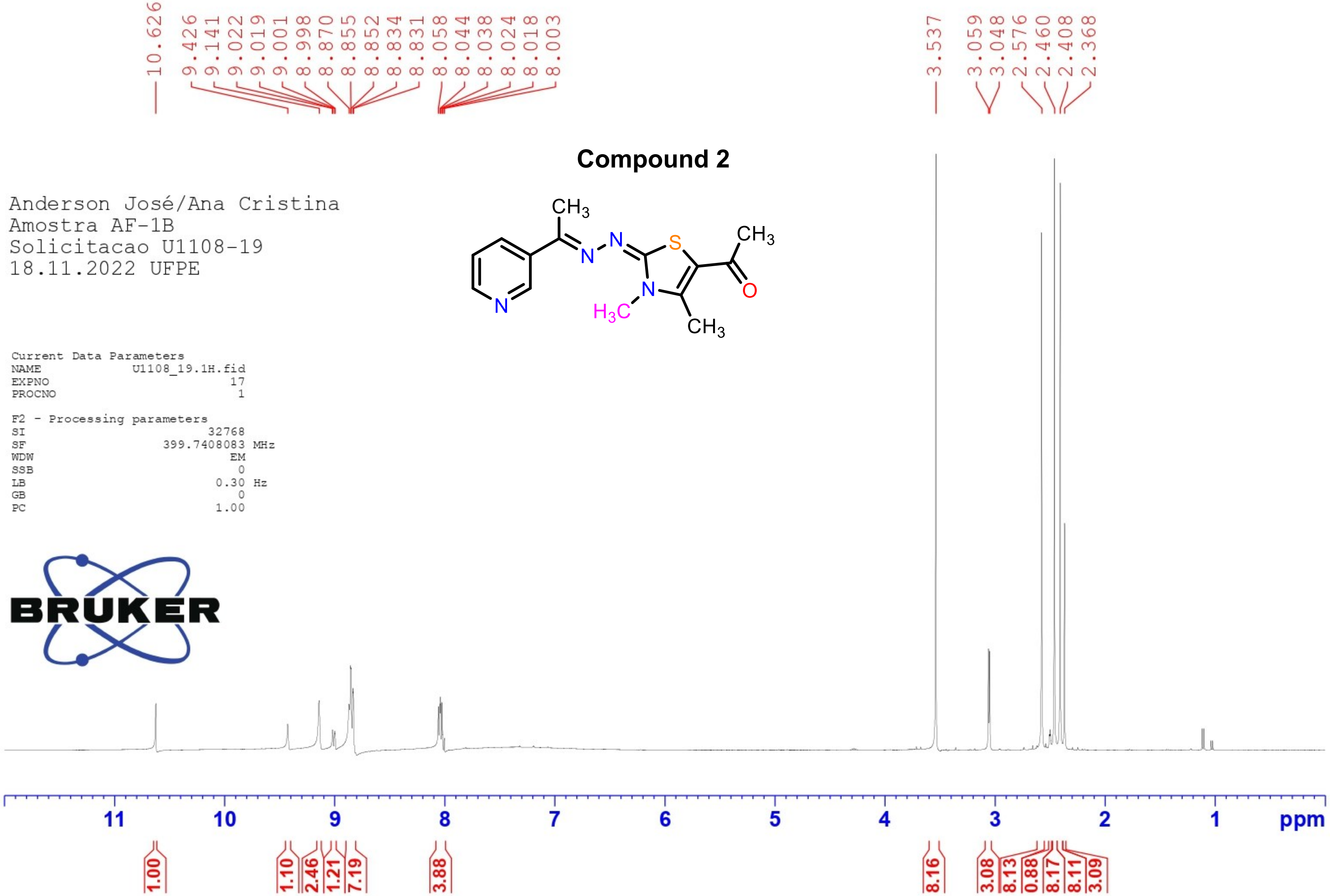

Compound 2
CH3
N
N
S
CH3
O
N
H3C
N
CH3
Anderson José/Ana Cristina
Amostra AF-1B
Solicitacao U1108-19
18.11.2022 UFPE
Current Data Parameters
NAME U1108_19.1H.fid
EXPNO 17
PROCNO 1
F2 - Processing parameters
SI 32768
SF 399.7408083 MHz
WDW EM
SSB 0
LB 0.30 Hz
GB 0
PC 1.00
BRUKER
10.626
9.426
9.141
9.022
9.019
9.001
8.998
8.870
8.855
8.852
8.834
8.831
8.058
8.044
8.038
8.024
8.018
8.003
3.537
3.059
3.048
2.576
2.460
2.408
2.368
11
10
9
8
7
6
5
4
3
2
1
ppm
1.00
1.10
2.46
1.21
7.19
3.88
8.16
3.08
8.13
0.88
8.17
8.11
3.09

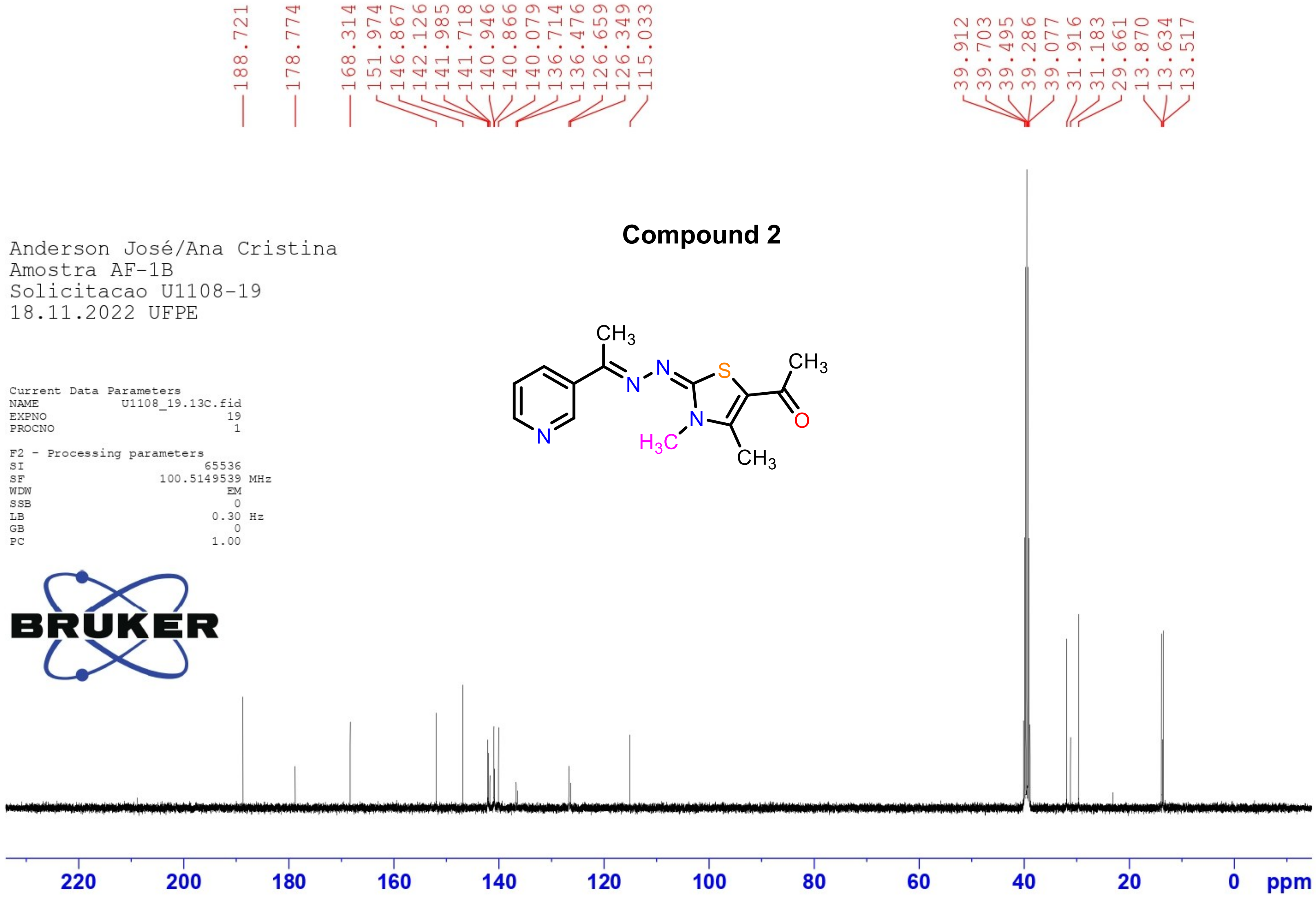
188.721
178.774
168.314
151.974
146.867
142.126
141.985
141.718
140.946
140.866
140.079
136.714
136.476
126.659
126.349
115.033
39.912
39.703
39.495
39.286
39.077
31.916
31.183
29.661
13.870
13.634
13.517
Anderson José/Ana Cristina
Amostra AF-1B
Solicitacao U1108-19
18.11.2022 UFPE
Current Data Parameters
NAME U1108_19.13C.fid
EXPNO 19
PROCNO 1
F2 - Processing parameters
SI 65536
SF 100.5149539 MHz
WDW EM
SSB 0
LB 0.30 Hz
GB 0
PC 1.00
CH3
N
N
S
CH3
O
N
H3C
N
CH3
220
200
180
160
140
120
100
80
60
40
20
0
ppm

Compound 2

Anderson José/Ana Cristina
Amostra AF-1C
Solicitacao U1108-24
23.11.2022 UFPE

Compound 3

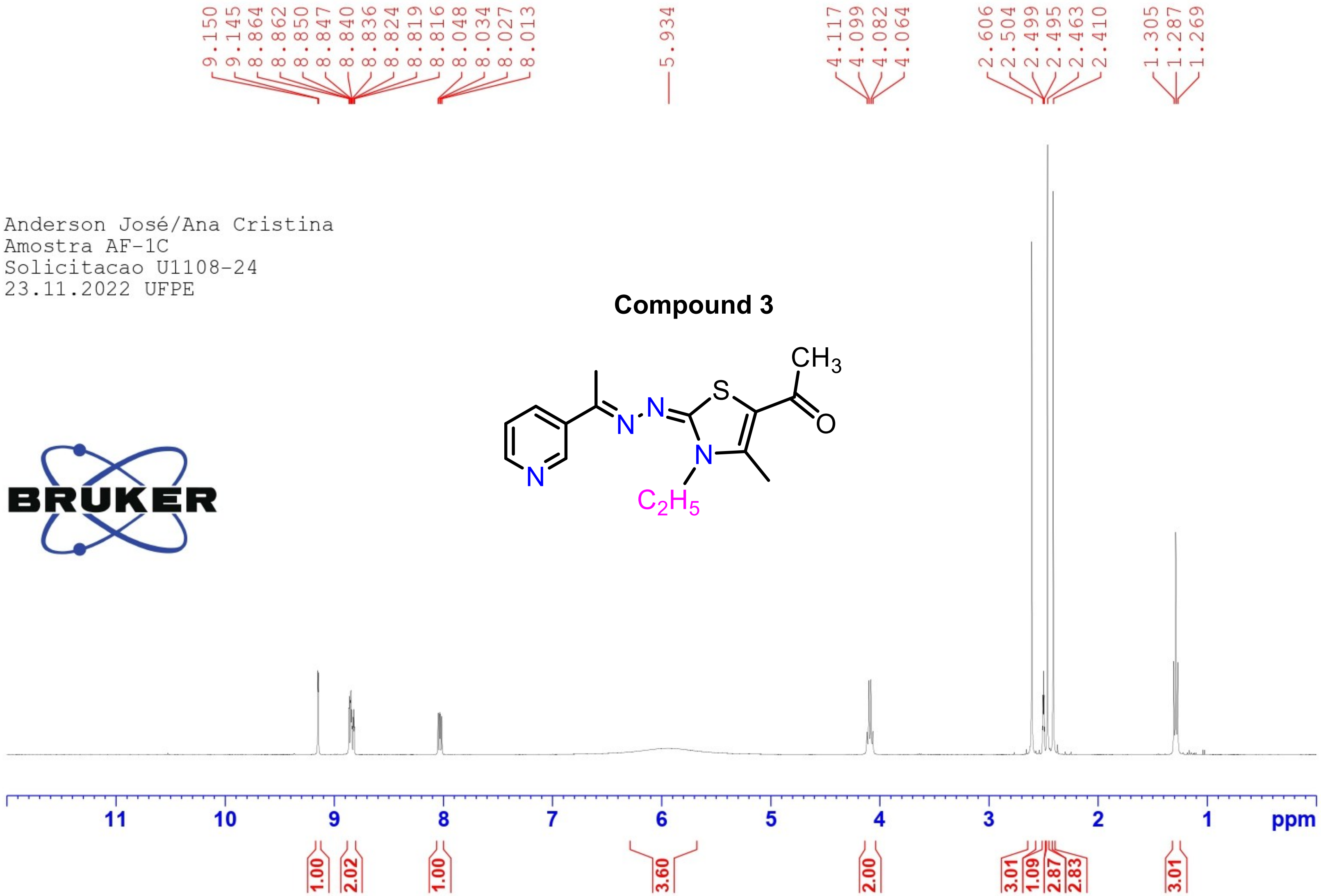

Anderson JosØ/Ana Cristina
Amostra AF-1C
Solicitacao U1108-24
23.11.2022 UFPE

Compound 3

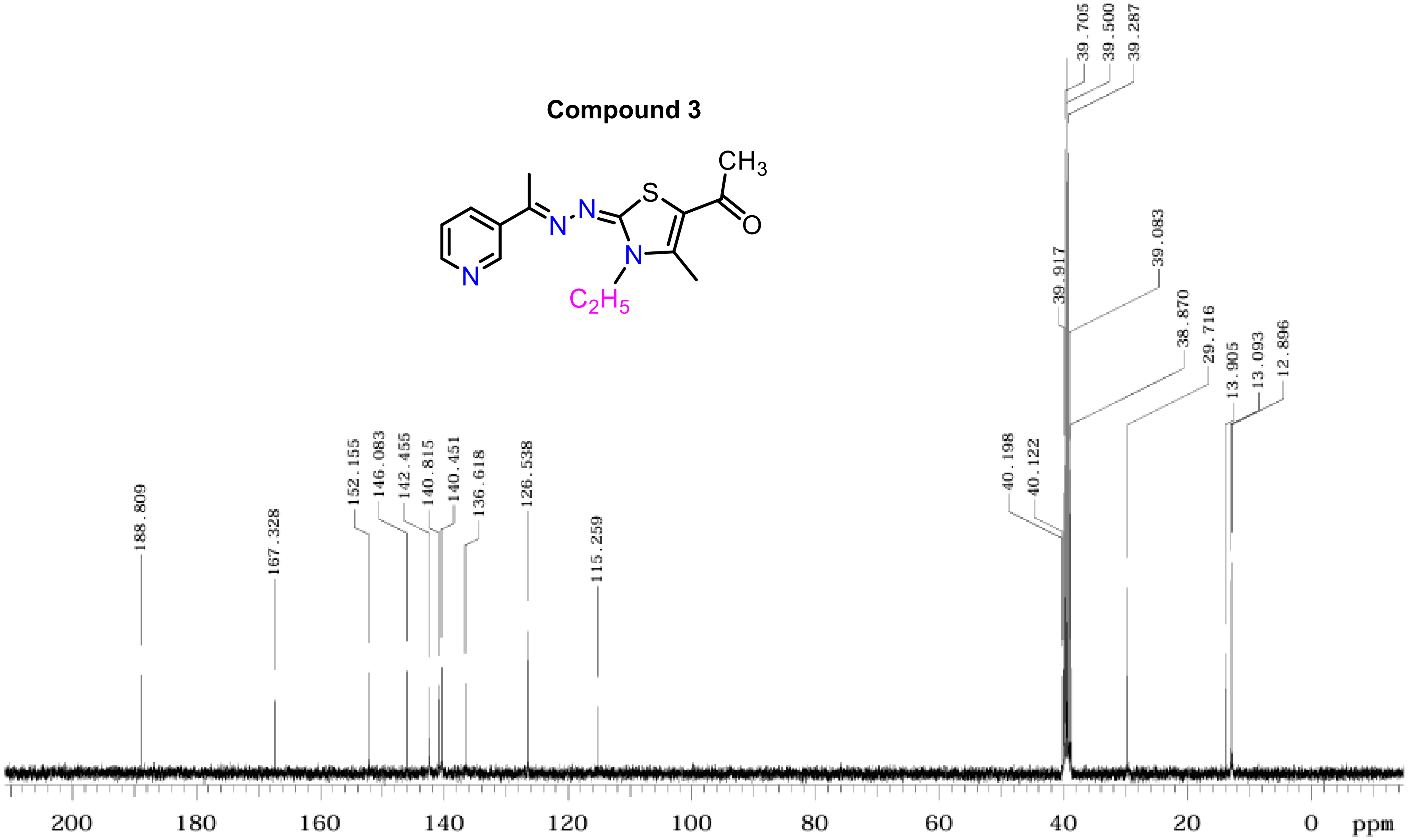

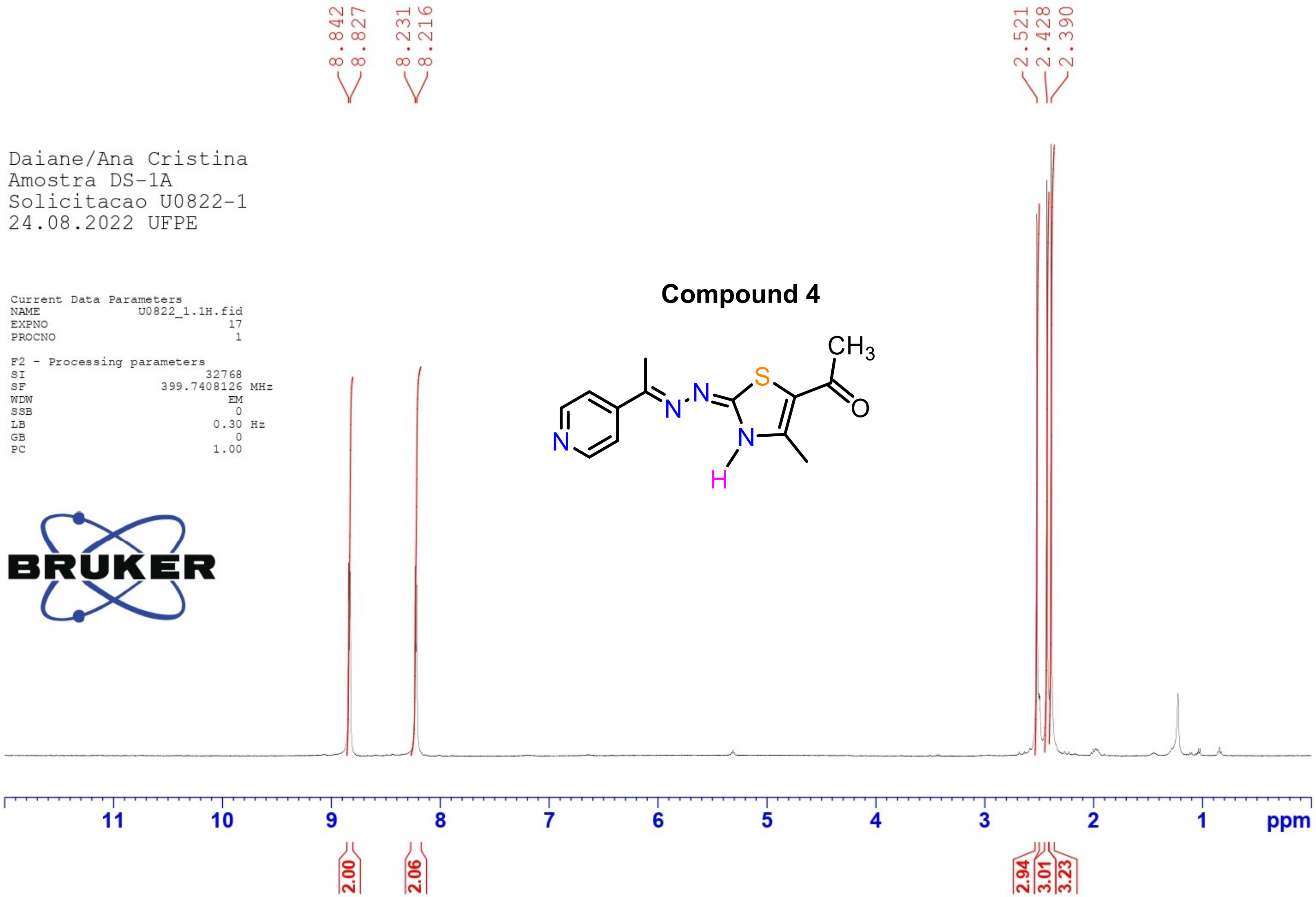
8.842
8.827
8.231
8.216
2.521
2.428
2.390
Daiane/Ana Cristina
Amostra DS-1A
Solicitacao U0822-1
24.08.2022 UFPE
Current Data Parameters
NAME U0822_1.1H.fid
EXPNO 17
PROCNO 1
F2 - Processing parameters
SI 32768
SF 399.7408126 MHz
WDW EM
SSB 0
LB 0.30 Hz
GB 0
PC 1.00
Compound 4
CH3
S
N
O
H
11
10
9
8
7
6
5
4
3
2
1
ppm
2.00
2.06
2.94
3.01
3.23

Daiane/Ana Cristina
Amostra DS-1A
Solicitacao U0822-1
24.08.2022 UFPE

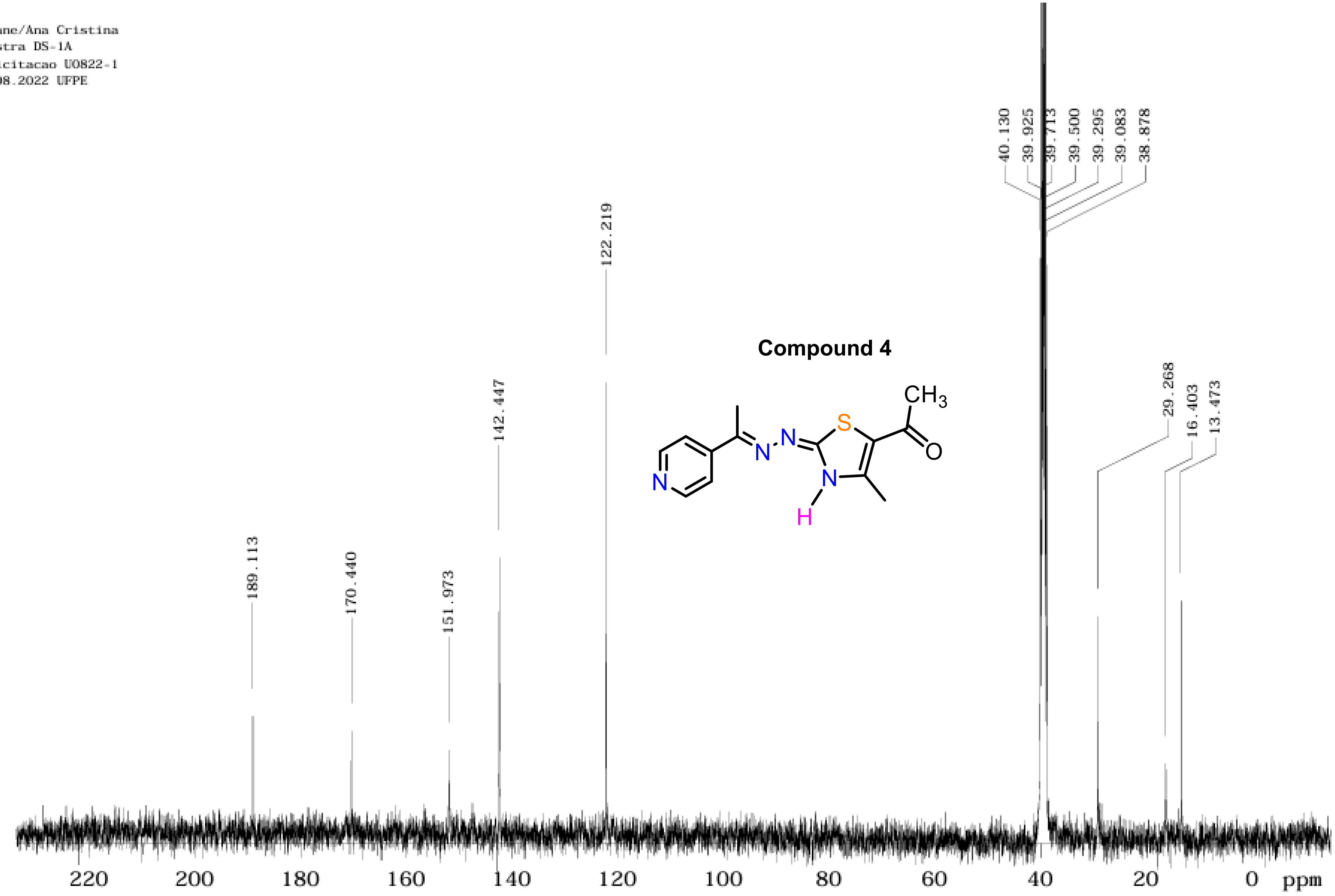

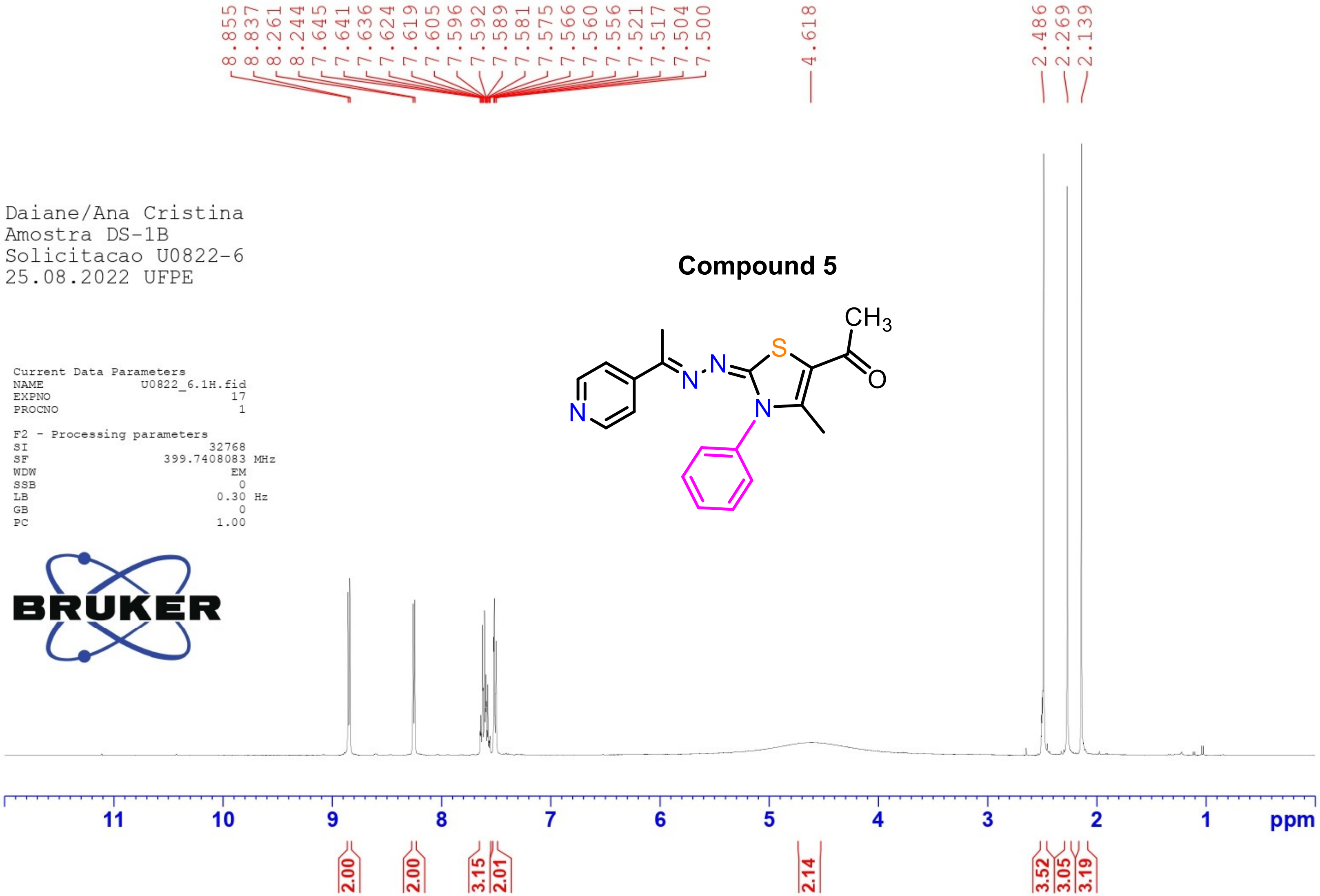
8.855 8.837 8.261 8.244 7.645 7.641 7.636 7.624 7.619 7.605 7.596 7.592 7.589 7.581 7.575 7.566 7.560 7.556 7.521 7.517 7.504 7.500
4.618
2.486 2.269 2.139
Daiane/Ana Cristina
Amostra DS-1B
Solicitacao U0822-6
25.08.2022 UFPE
Compound 5
CH3
S
N
N
N
N
O
Current Data Parameters
NAME U0822_6.1H.fid
EXPNO 17
PROCNO 1
F2 - Processing parameters
SI 32768
SF 399.7408083 MHz
WDW EM
SSB 0
LB 0.30 Hz
GB 0
PC 1.00
BRUKER
11 10 9 8 7 6 5 4 3 2 1 ppm
2.00 2.00 3.15 2.01 2.14 3.52 3.05 3.19

Compound 5

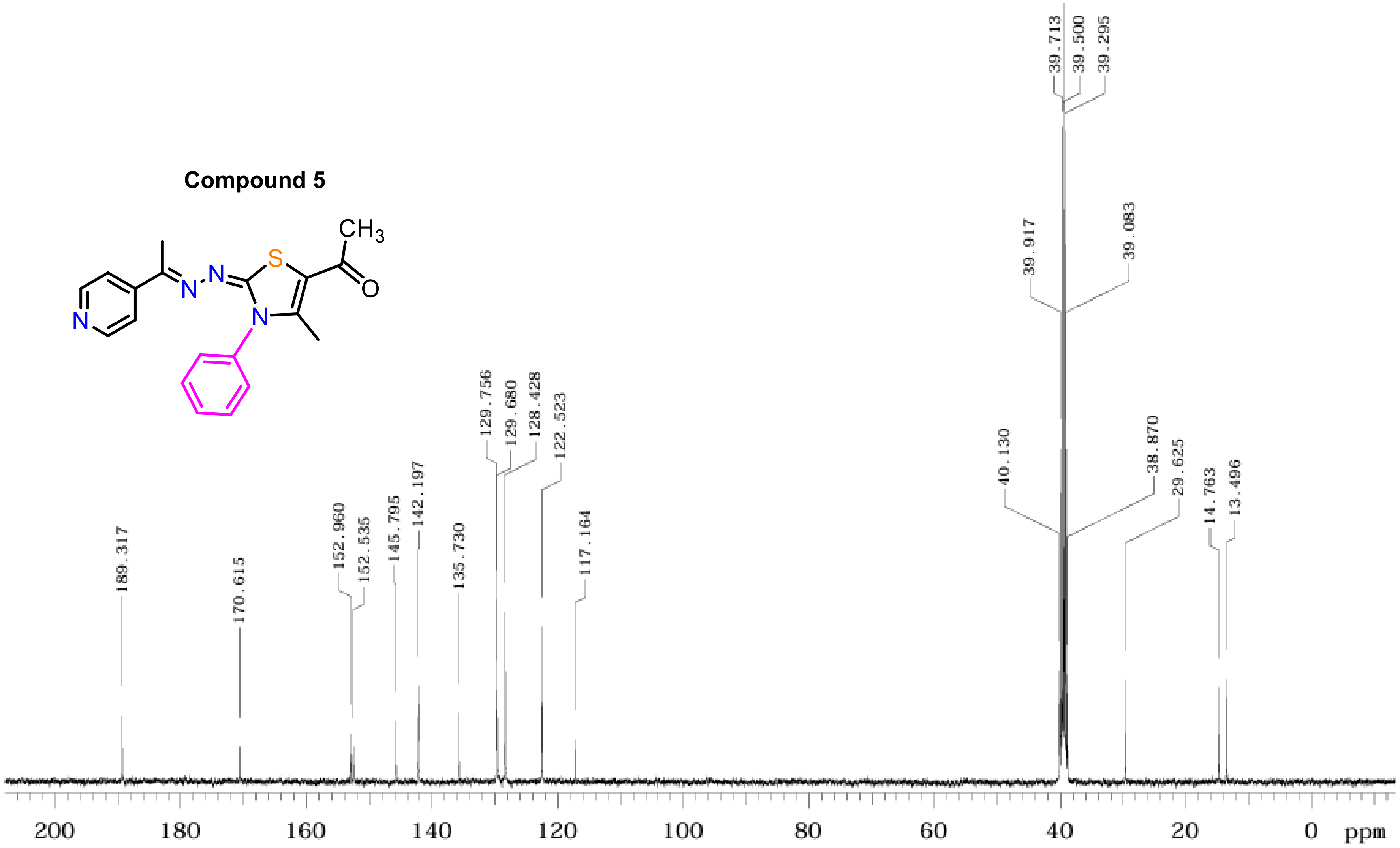
CH3
S
N
N
N
O
N
189.317
170.615
152.960
152.535
145.795
142.197
135.730
129.756
129.680
128.428
122.523
117.164
40.130
39.917
39.713
39.500
39.295
39.083
38.870
29.625
14.763
13.496
200
180
160
140
120
100
80
60
40
20
0
ppm

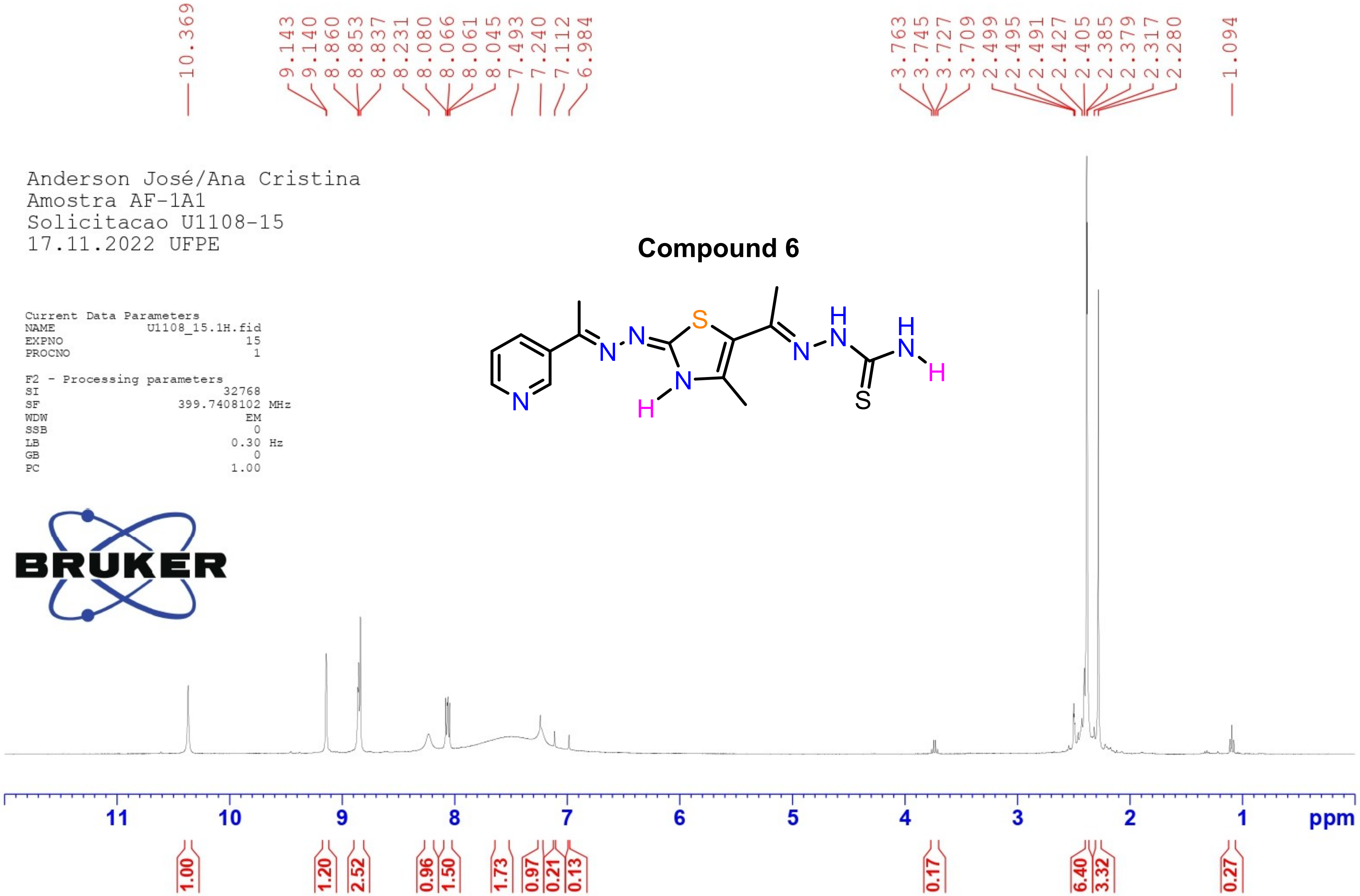

Anderson José/Ana Cristina
Amostra AF-1A1
Solicitacao U1108-15
17.11.2022 UFPE
Current Data Parameters
NAME U1108_15.1H.fid
EXPNO 15
PROCNO 1
F2 - Processing parameters
SI 32768
SF 399.7408102 MHz
WDW EM
SSB 0
LB 0.30 Hz
GB 0
PC 1.00
BRUKER
Compound 6
N
N
N
S
N
H
N
H
N
H
N
H
S
10.369
9.143
9.140
8.860
8.853
8.837
8.231
8.080
8.066
8.061
8.045
7.493
7.240
7.112
6.984
3.763
3.745
3.727
3.709
2.499
2.495
2.491
2.427
2.405
2.385
2.379
2.317
2.280
1.094
11
10
9
8
7
6
5
4
3
2
1
ppm
1.00
1.20
2.52
0.96
1.50
1.73
0.97
0.21
0.13
0.17
6.40
3.32
0.27

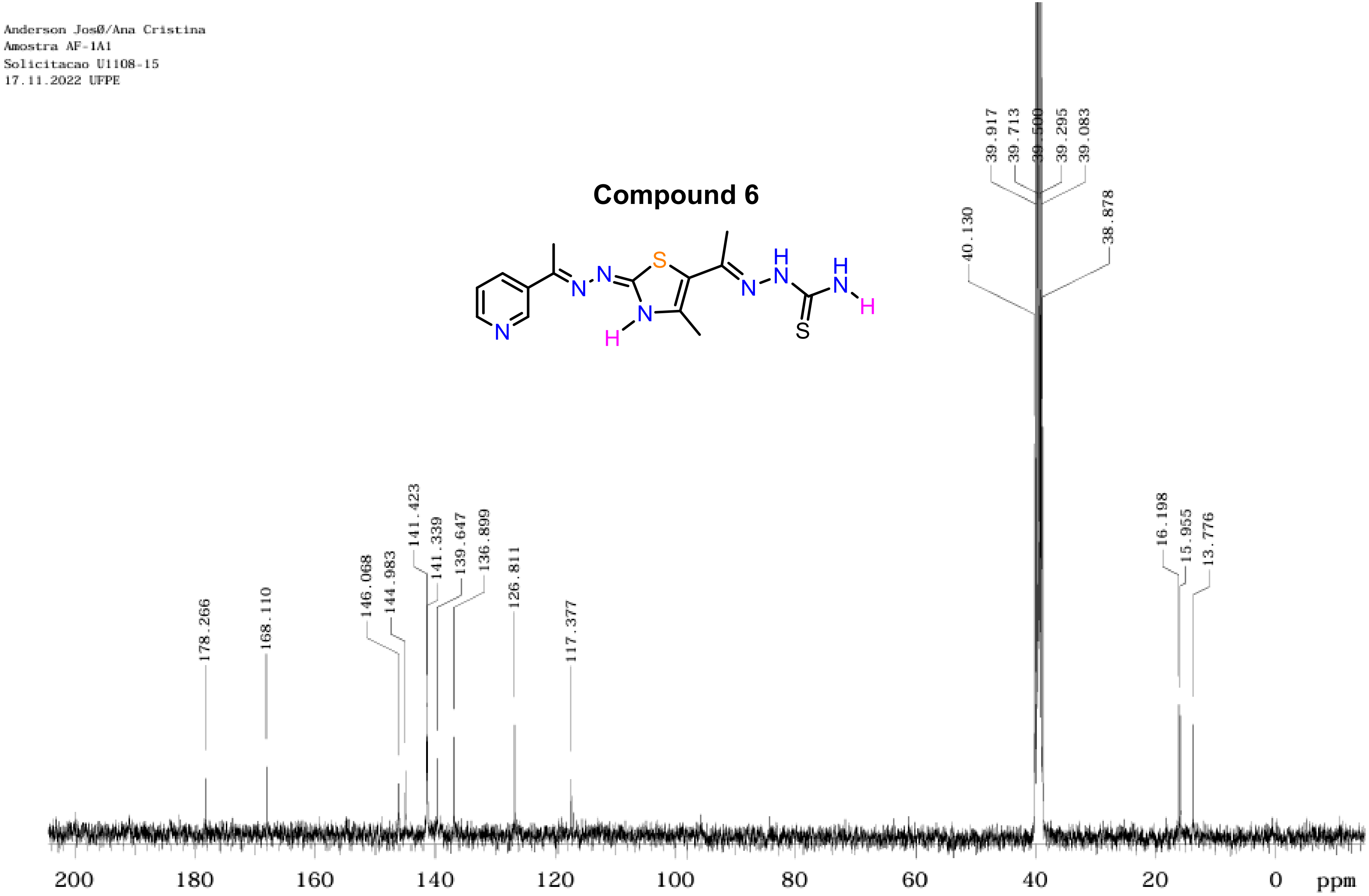
Anderson JosØ/Ana Cristina
Amostra AF-1A1
Solicitacao U1108-15
17.11.2022 UFPE
Compound 6
178.266
168.110
146.068
144.983
141.423
141.339
139.647
136.899
126.811
117.377
40.130
39.917
39.713
39.500
39.295
39.083
38.878
16.198
15.955
13.776
200
180
160
140
120
100
80
60
40
20
0
ppm

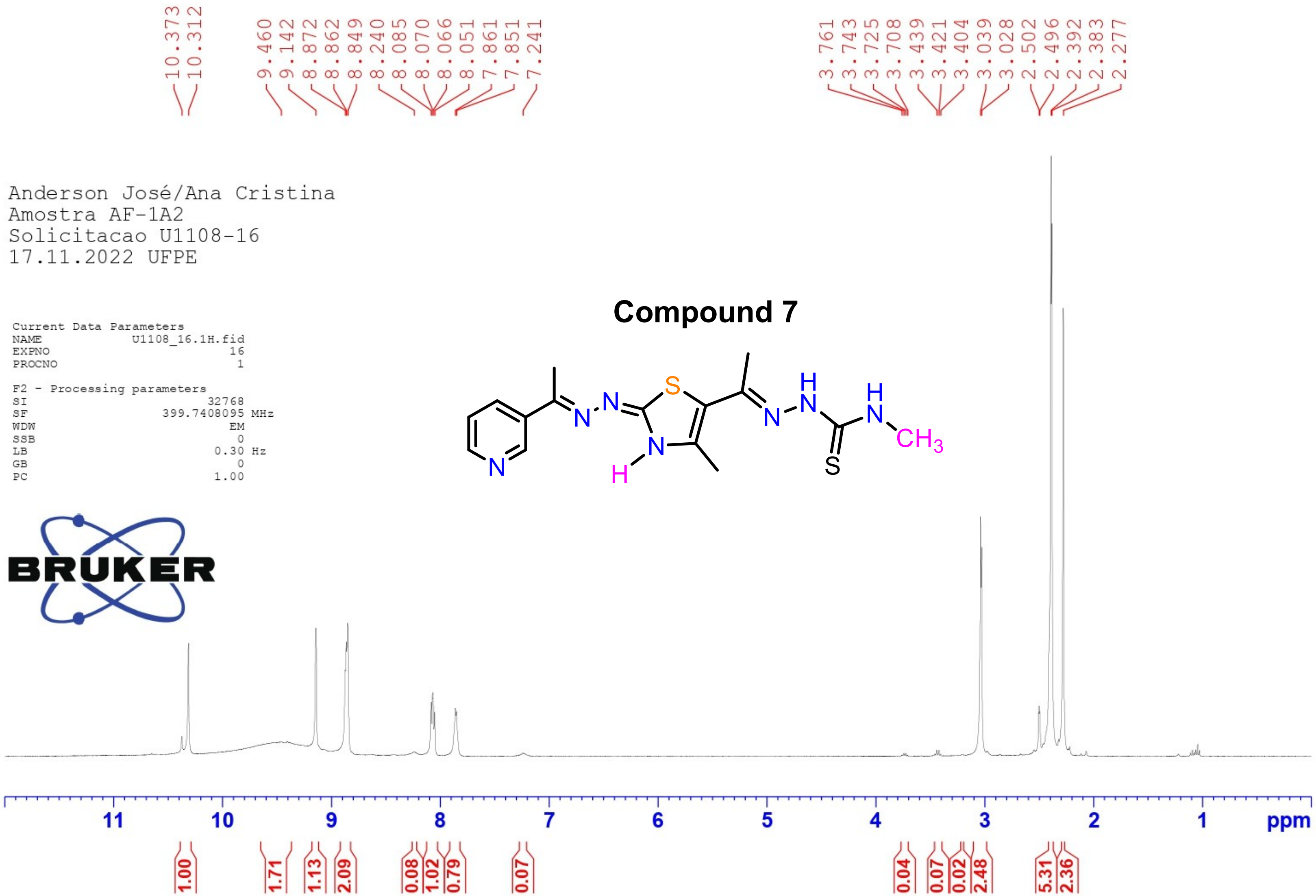
10.373
10.312
9.460
9.142
8.872
8.862
8.849
8.240
8.085
8.070
8.066
8.051
7.861
7.851
7.241
3.761
3.743
3.725
3.708
3.439
3.421
3.404
3.039
3.028
2.502
2.496
2.392
2.383
2.277
Anderson José/Ana Cristina
Amostra AF-1A2
Solicitacao U1108-16
17.11.2022 UFPE
Current Data Parameters
NAME U1108_16.1H.fid
EXPNO 16
PROCNO 1
F2 - Processing parameters
SI 32768
SF 399.7408095 MHz
WDW EM
SSB 0
LB 0.30 Hz
GB 0
PC 1.00
Compound 7
ppm
1.00
1.71
1.13
2.09
0.08
1.02
0.79
0.07
0.04
0.07
0.02
2.48
5.31
2.36

Anderson JosØ/Ana Cristina
Amostra AF-1A2
Solicitacao U1108-16
17.11.2022 UFPE

Compound 7

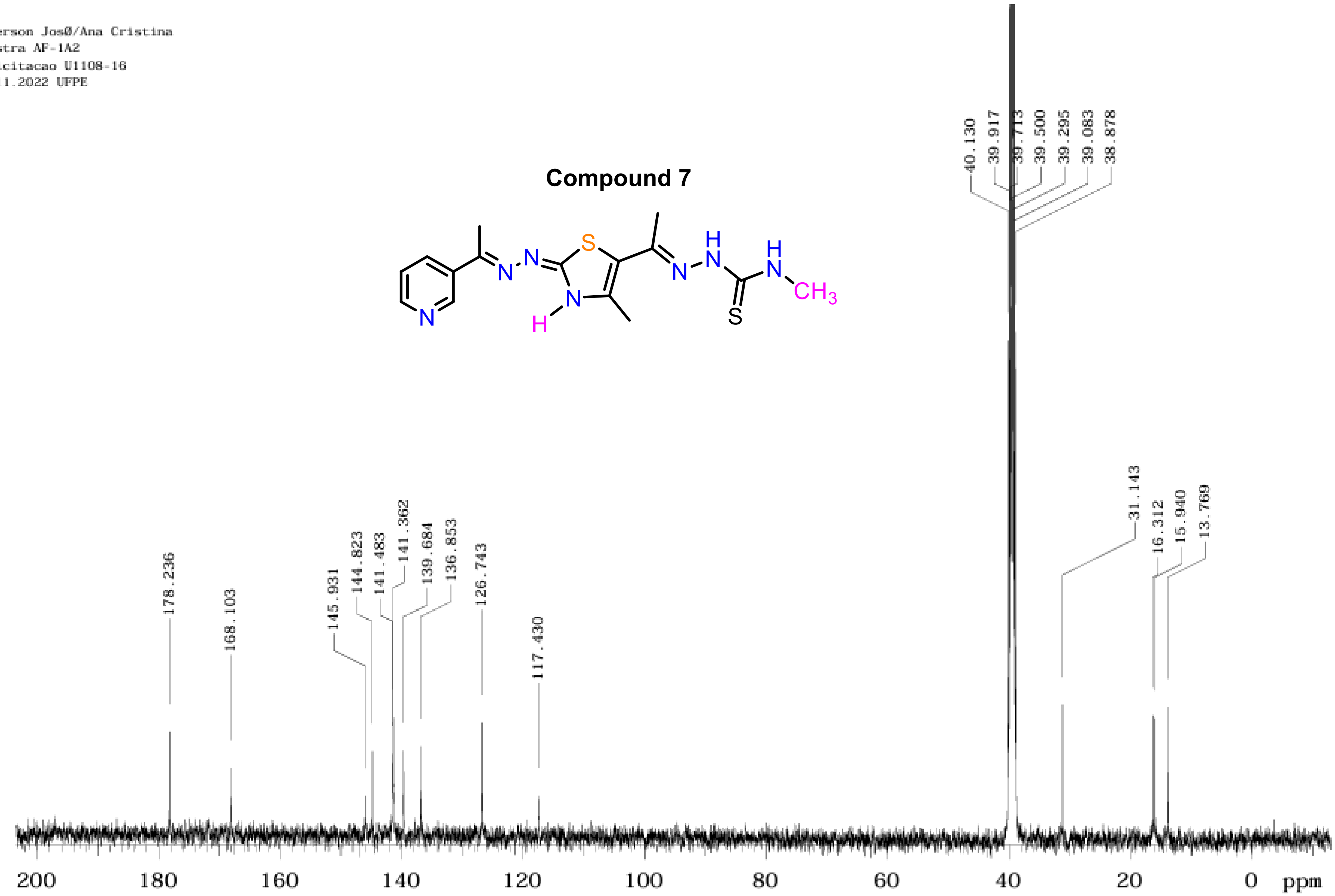

Anderson José/Ana Cristina
Amostra AF-1A3
Solicitacao U1108-17
17.11.2022 UFPE

```
Current Data Parameters
NAME              U1108_17.1H.fid
EXPNO                          17
PROCNO                          1

F2 - Processing parameters
SI                          32768
SF                 399.7408087 MHz
WDW                            EM
SSB                             0
LB                           0.30 Hz
GB                              0
PC                           1.00
```

Compound 8

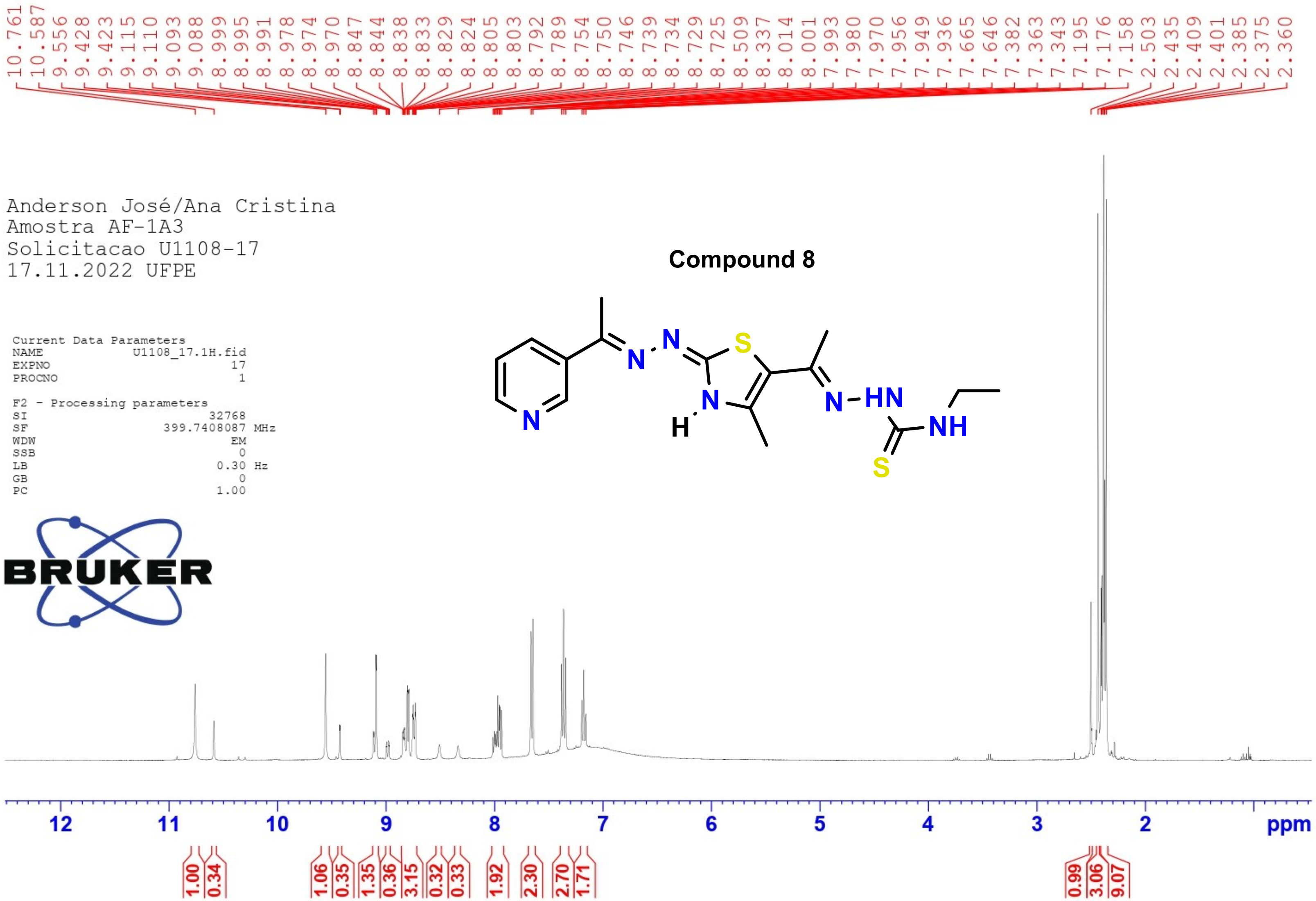

Anderson José/Ana Cristina
Amostra AF-1A3
Solicitacao U1108-17
17.11.2022 UFPE

Compound 8

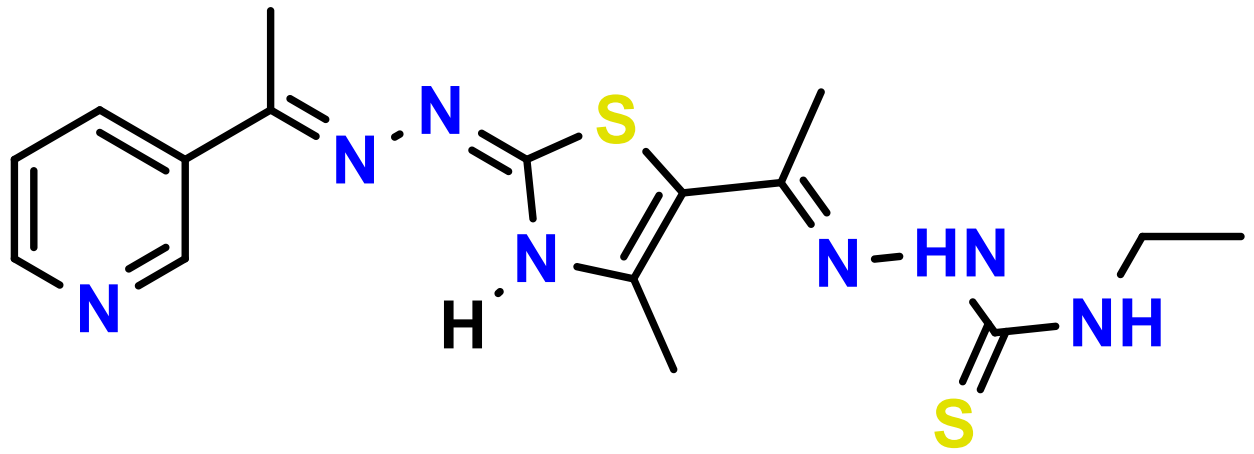

Current Data Parameters
NAME U1108_17.13C.fid
EXPNO 17
PROCNO 1

F2 - Processing parameters
SI 65536
SF 100.5149544 MHz
WDW EM
SSB 0
LB 0.30 Hz
GB 0
PC 1.00

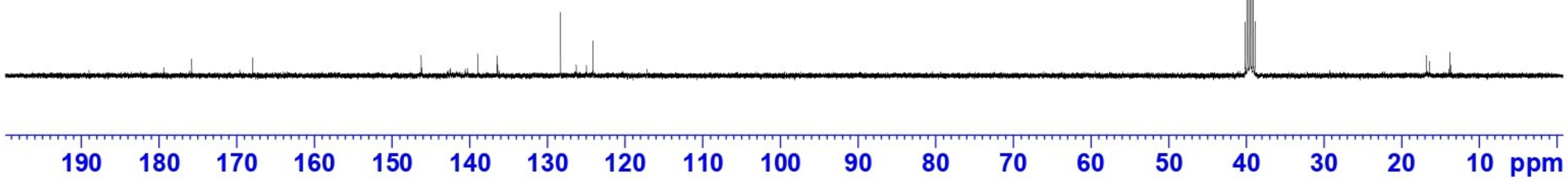

Anderson José/Ana Cristina
Amostra AF-1A4
Solicitacao U1108-18
17.11.2022 UFPE

Compound 9

Current Data Parameters
NAME U1108_18.1H.fid
EXPNO 17
PROCNO 1

F2 - Processing parameters
SI 32768
SF 399.7408083 MHz
WDW EM
SSB 0
LB 0.30 Hz
GB 0
PC 1.00

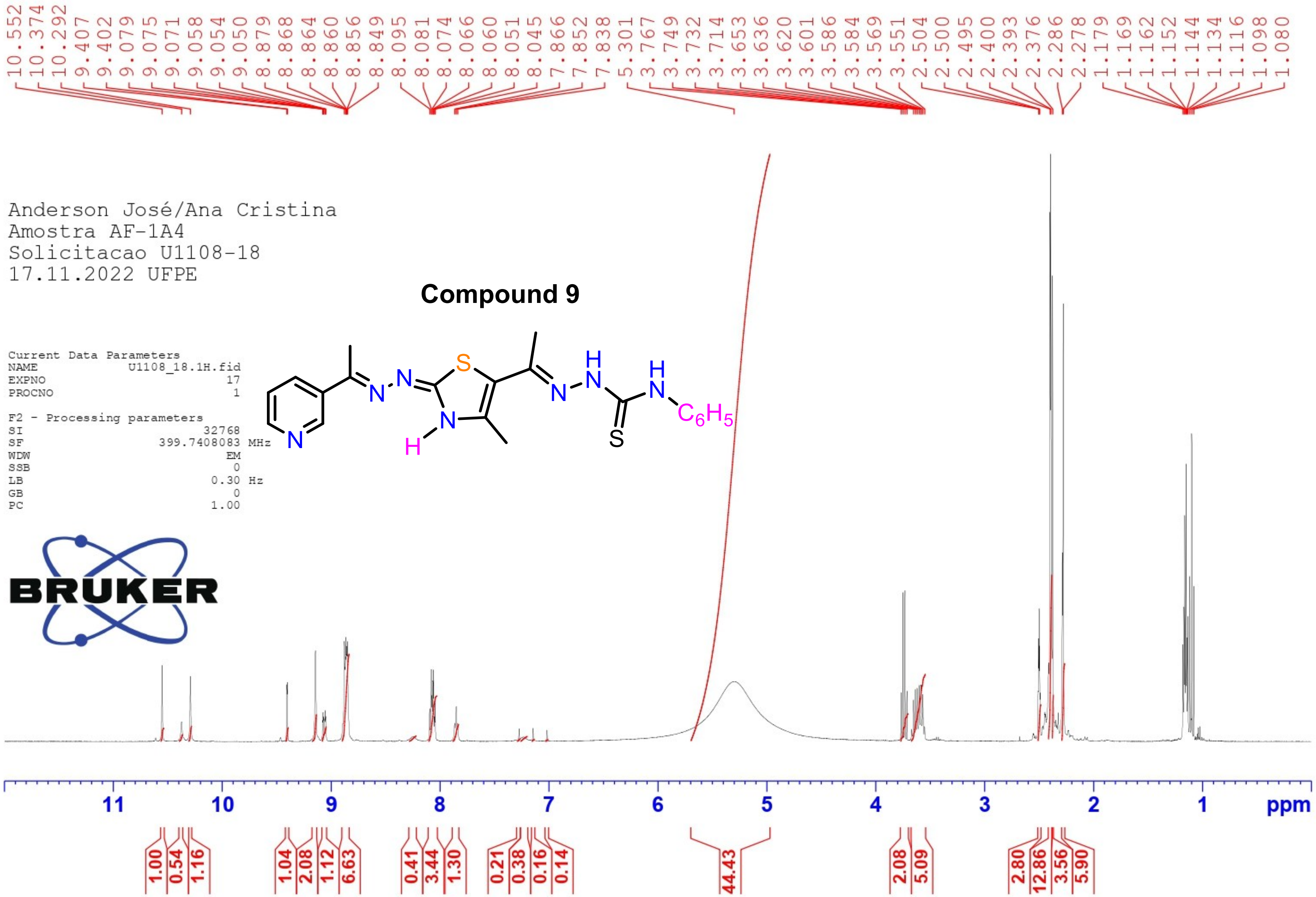

Anderson José/Ana Cristina
Amostra AF-1A4
Solicitacao U1108-18
17.11.2022 UFPE

```
Current Data Parameters
NAME            U1108_18.13C.fid
EXPNO                         13
PROCNO                         1

F2 - Processing parameters
SI                         65536
SF                100.5149500 MHz
WDW                           EM
SSB                            0
LB                          0.30 Hz
GB                             0
PC                          1.00
```

Compound 9

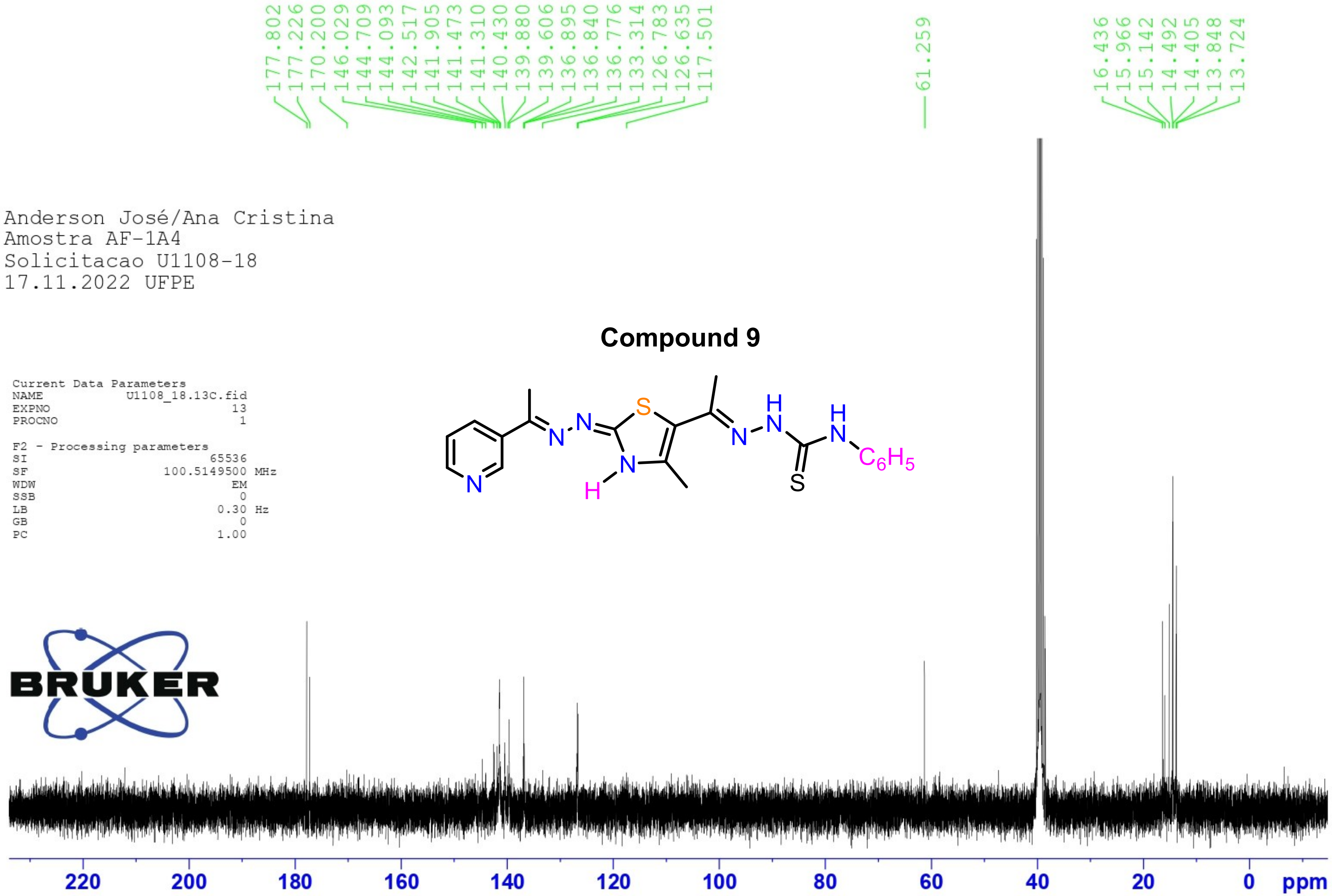

Anderson José/Ana Cristina
Amostra AF-1B1
Solicitacao U1108-20
18.11.2022 UFPE

Compound 10

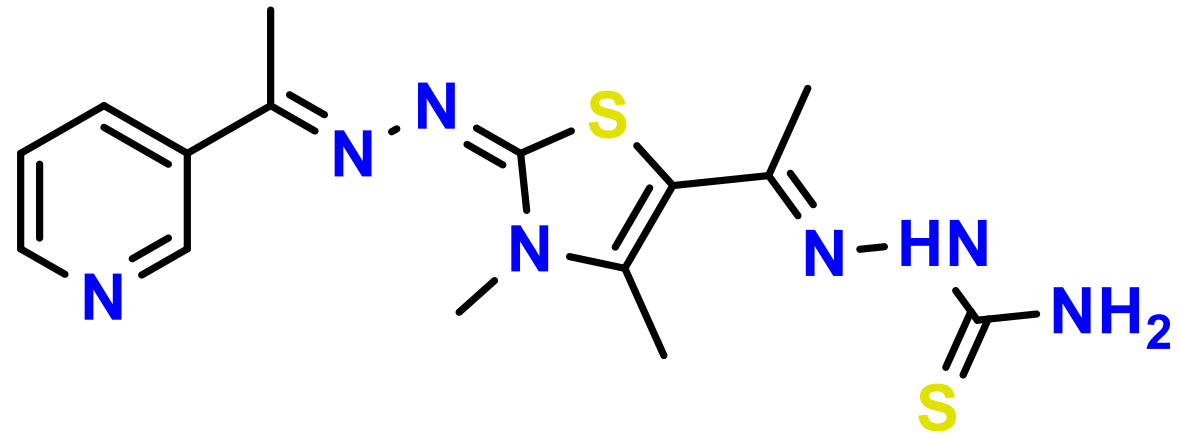

Current Data Parameters
NAME U1108_20.1H.fid
EXPNO 19
PROCNO 1

F2 - Processing parameters
SI 32768
SF 399.7408071 MHz
WDW EM
SSB 0
LB 0.30 Hz
GB 0
PC 1.00

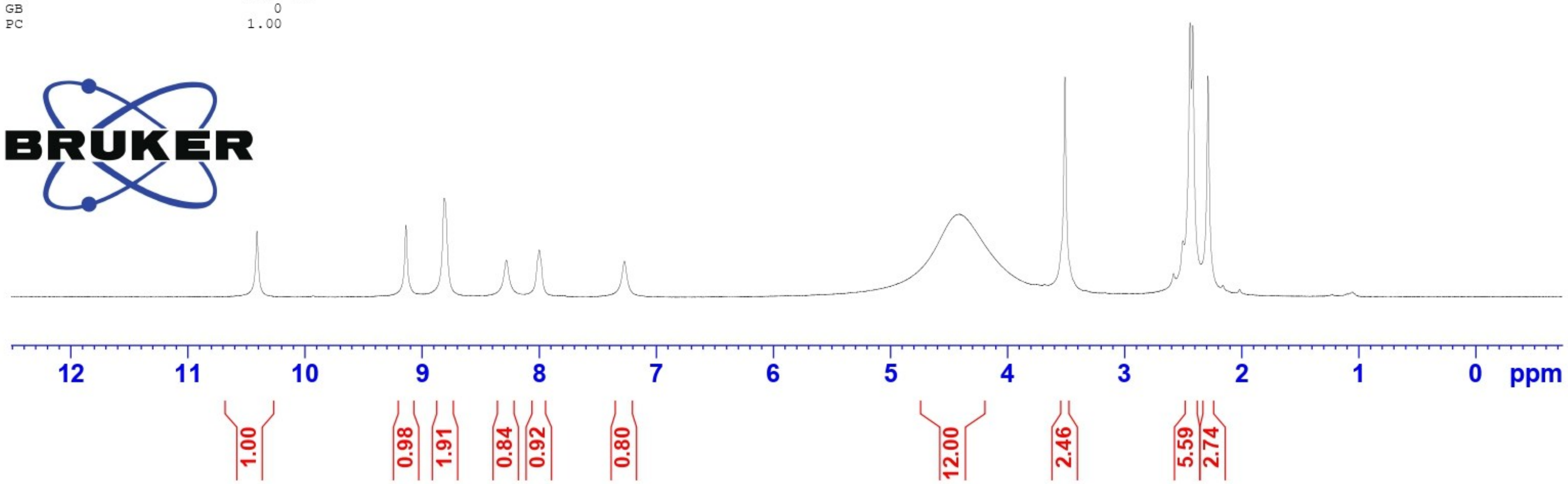

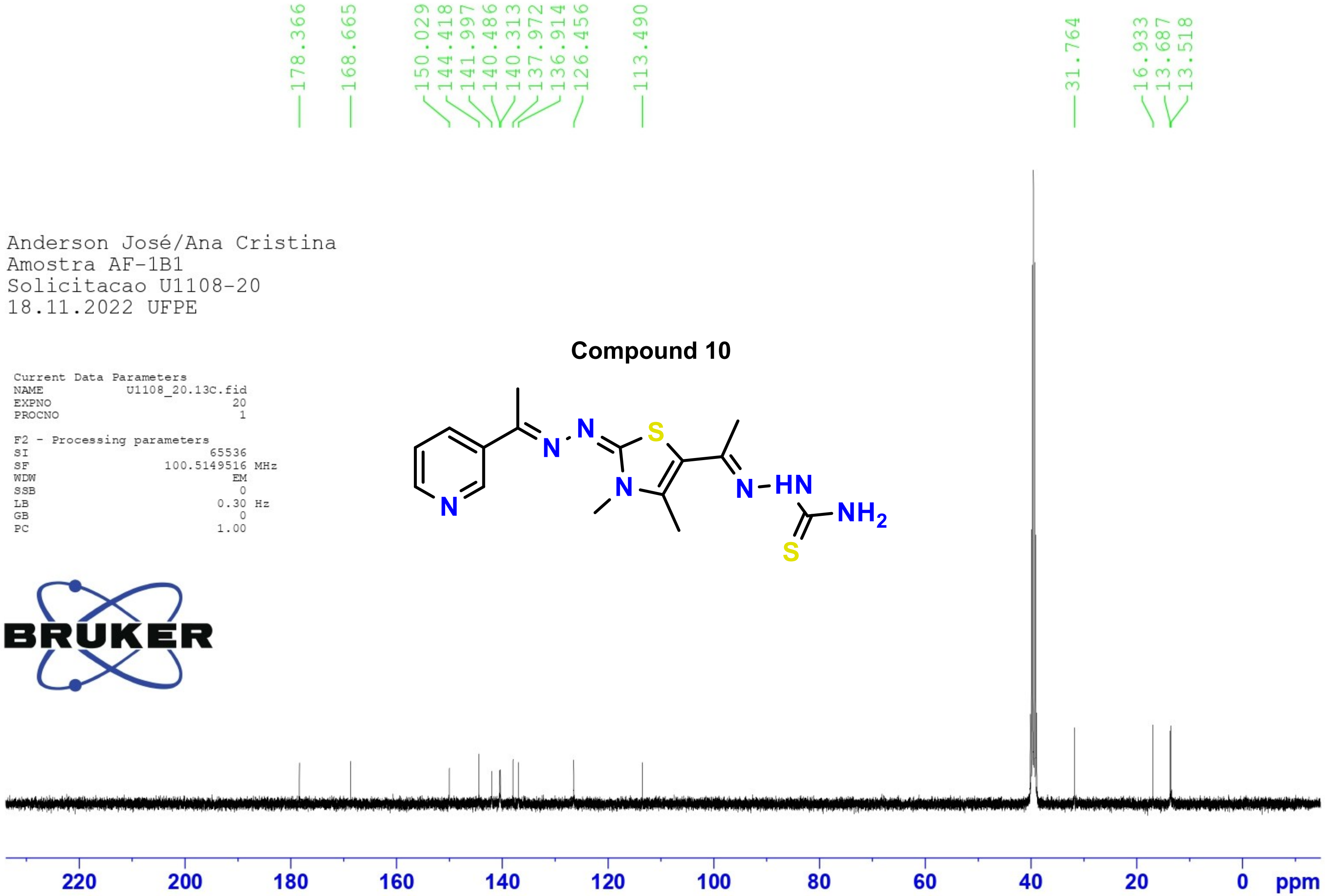

Anderson José/Ana Cristina
Amostra AF-1B1
Solicitacao U1108-20
18.11.2022 UFPE

Current Data Parameters		
NAME	U1108_20.13C.fid	
EXPNO	20	
PROCNO	1	
F2 - Processing parameters		
SI	65536	
SF	100.5149516	MHz
WDW	EM	
SSB	0	
LB	0.30	Hz
GB	0	
PC	1.00	

Compound 10

Anderson José/Ana Cristina
Amostra AF-1B3
Solicitacao U1108-22
17.11.2022 UFPE

```
Current Data Parameters
NAME          U1108_22.1H.fid
EXPNO                      17
PROCNO                      1

F2 - Processing parameters
SI                      32768
SF            399.7408083 MHz
WDW                        EM
SSB                         0
LB                    0.30 Hz
GB                          0
PC                       1.00
```

Compound 11

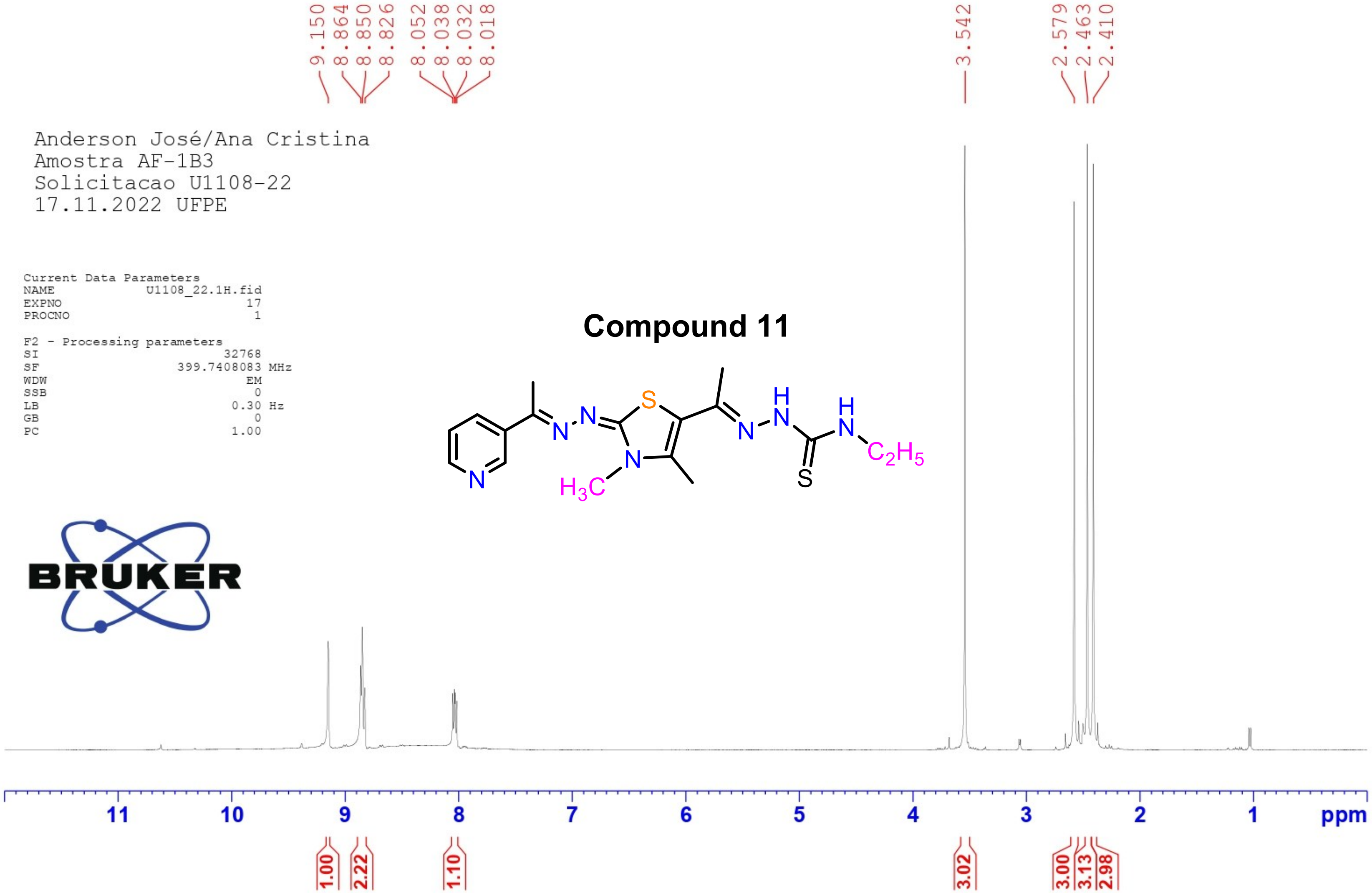

Anderson JosØ/Ana Cristina
Amostra AF-1B3
Solicitacao U1108-22
17.11.2022 UFPE

Compound 11

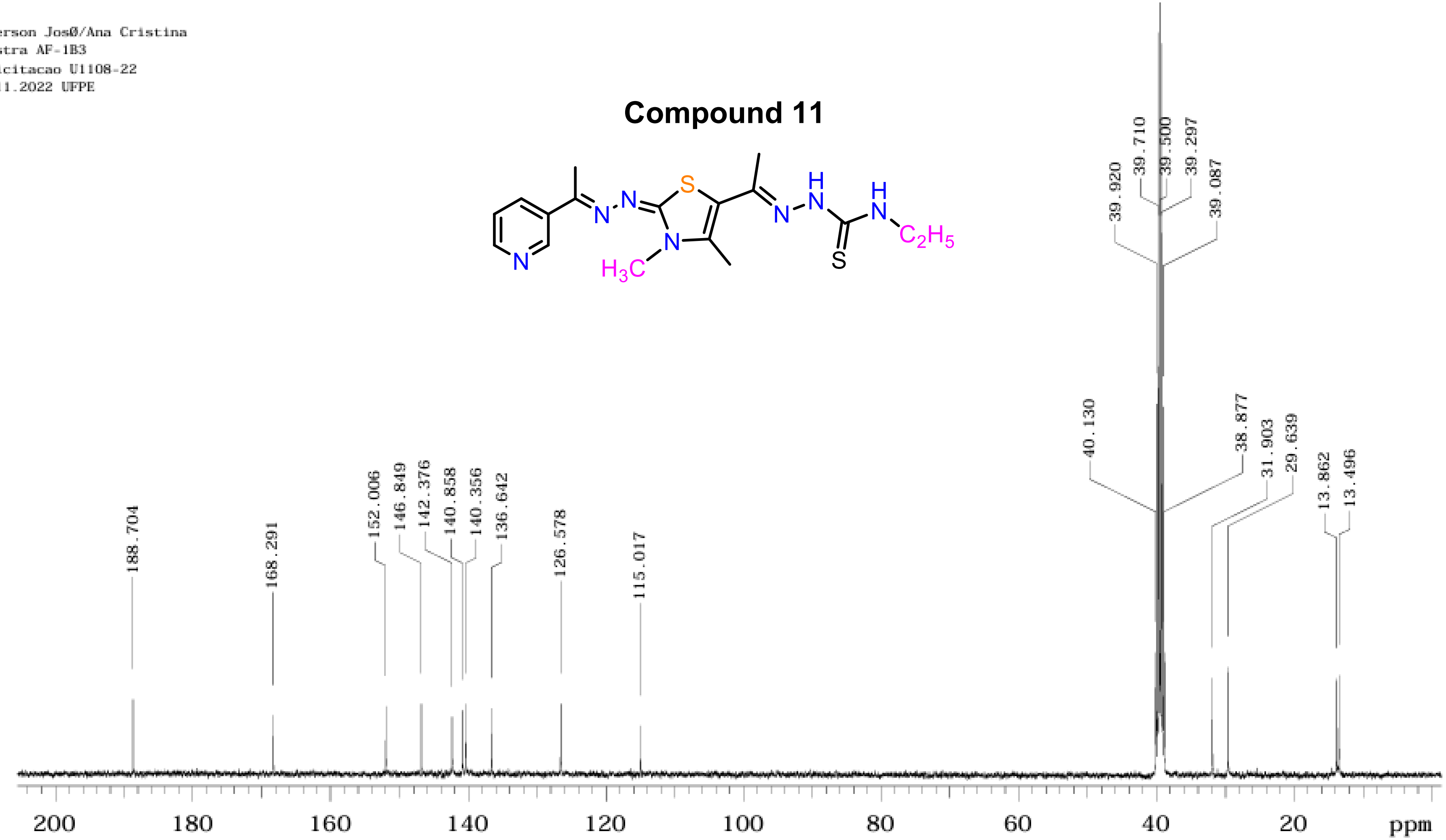

Anderson José/Ana Cristina
Amostra AF-1C1
Solicitacao U1108-25
23.11.2022 UFPE

```
Current Data Parameters
NAME          U1108_25.1H.fid
EXPNO                      17
PROCNO                      1

F2 - Processing parameters
SI                      32768
SF              399.7408117 MHz
WDW                        EM
SSB                         0
LB                       0.30 Hz
GB                          0
PC                       1.00
```

Compound 12

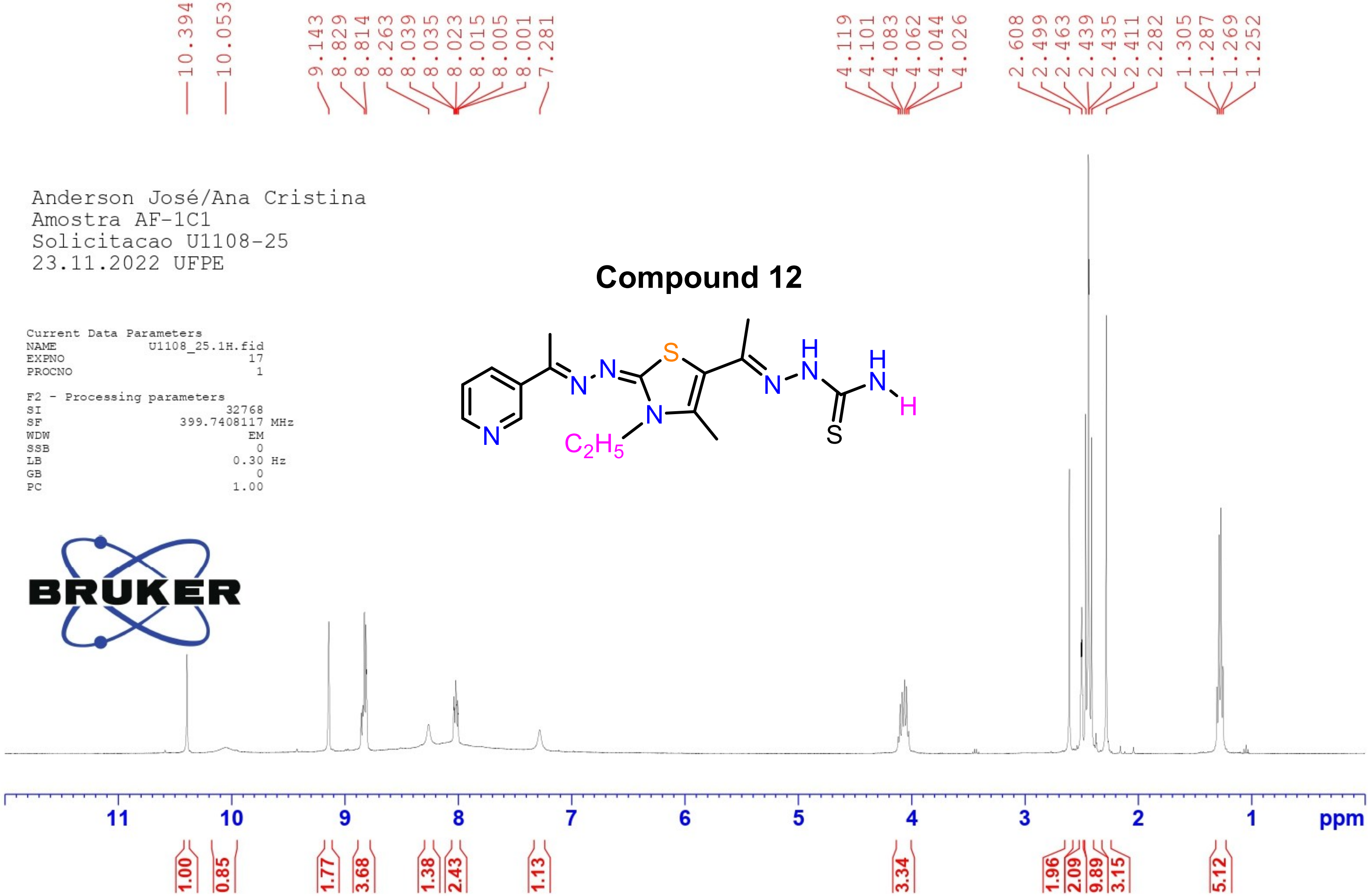

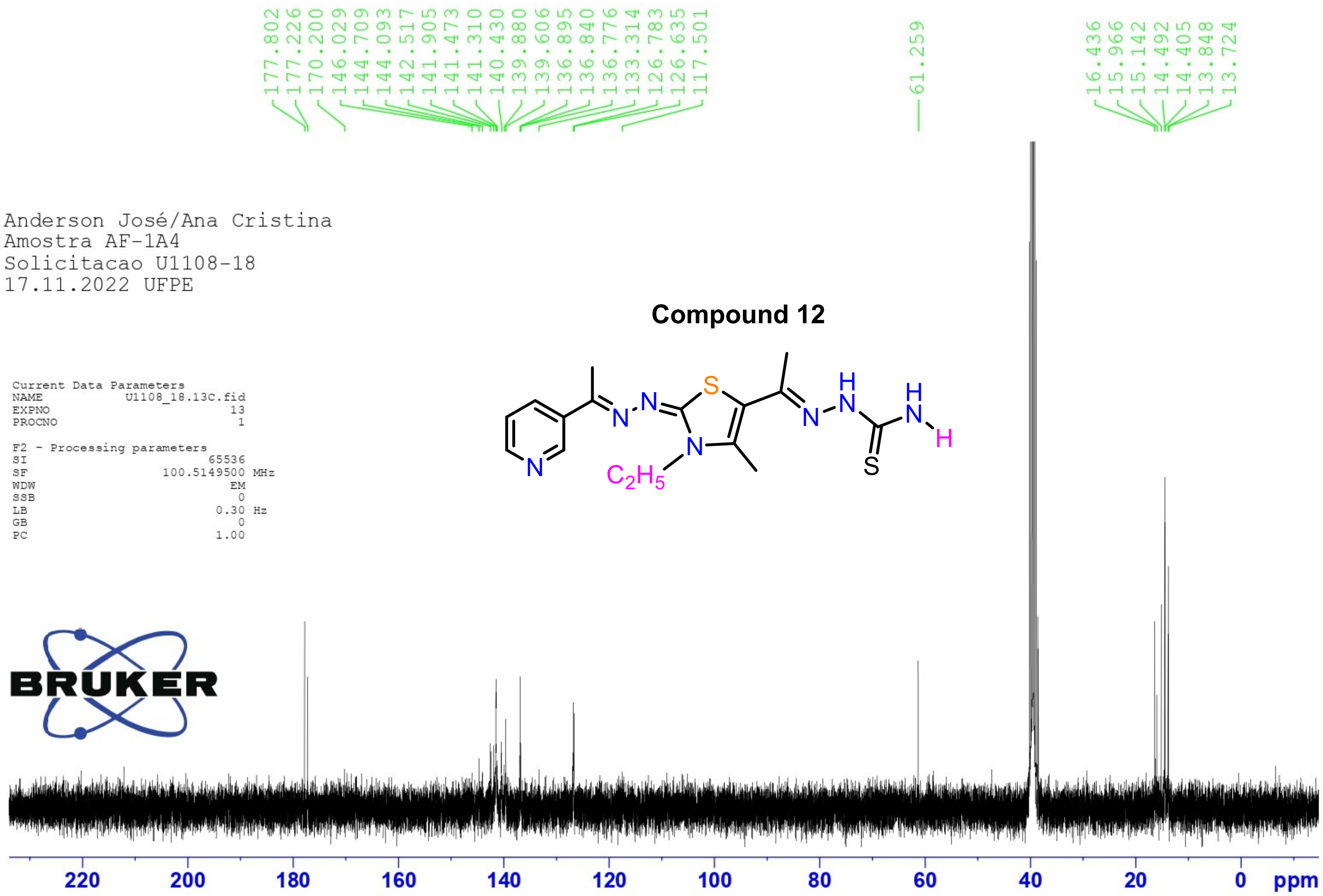
177.802
177.226
170.200
146.029
144.709
144.093
142.517
141.905
141.473
141.310
140.430
139.880
139.606
136.895
136.840
136.776
133.314
126.783
126.635
117.501
61.259
16.436
15.966
15.142
14.492
14.405
13.848
13.724
Anderson José/Ana Cristina
Amostra AF-1A4
Solicitacao U1108-18
17.11.2022 UFPE
Compound 12
N
N
N
S
N
C2H5
N
H
N
H
N
H
S
Current Data Parameters
NAME U1108_18.13C.fid
EXPNO 13
PROCNO 1
F2 - Processing parameters
SI 65536
SF 100.5149500 MHz
WDW EM
SSB 0
LB 0.30 Hz
GB 0
PC 1.00
220
200
180
160
140
120
100
80
60
40
20
0
ppm

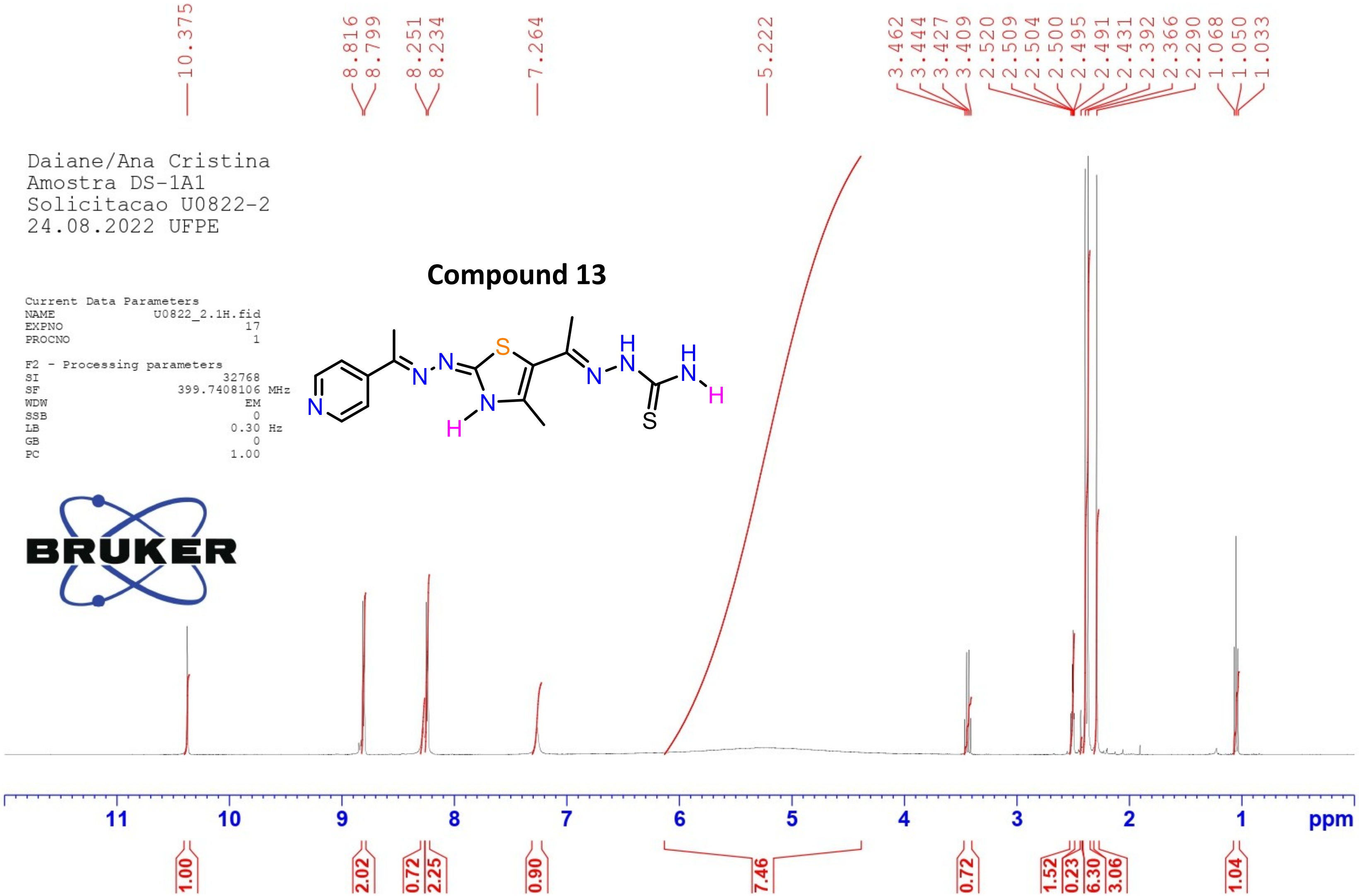
10.375
8.816
8.799
8.251
8.234
7.264
5.222
3.462
3.444
3.427
3.409
2.520
2.509
2.504
2.500
2.495
2.491
2.431
2.392
2.366
2.290
1.068
1.050
1.033
Daiane/Ana Cristina
Amostra DS-1A1
Solicitacao U0822-2
24.08.2022 UFPE
Compound 13
Current Data Parameters
NAME U0822_2.1H.fid
EXPNO 17
PROCNO 1
F2 - Processing parameters
SI 32768
SF 399.7408106 MHz
WDW EM
SSB 0
LB 0.30 Hz
GB 0
PC 1.00
BRUKER
11 10 9 8 7 6 5 4 3 2 1 ppm
1.00
2.02
0.72
2.25
0.90
7.46
0.72
1.52
0.23
6.30
3.06
1.04

Daiane/Ana Cristina
Amostra DS-1A1
Solicitacao U0822-2
24.08.2022 UFPE

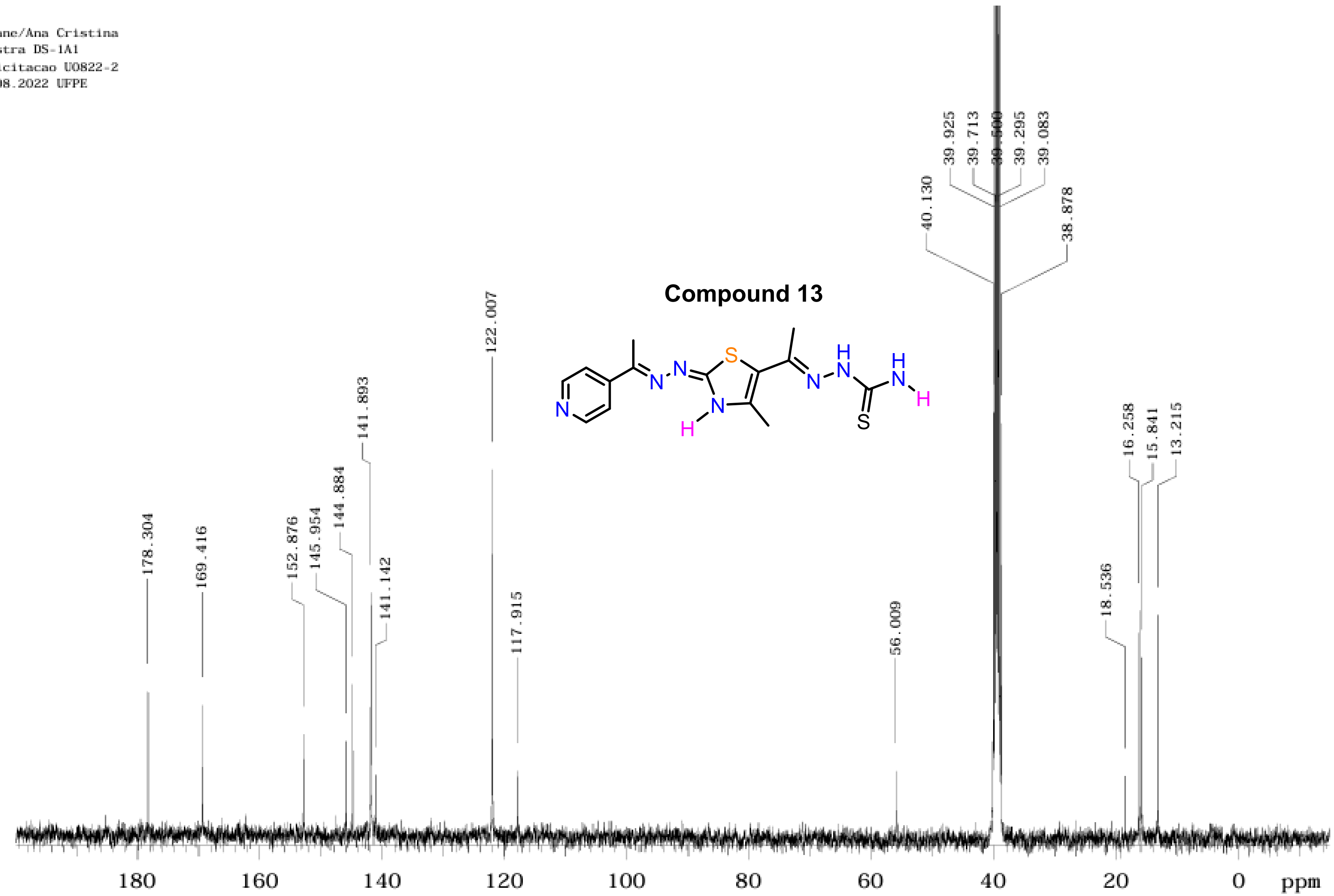

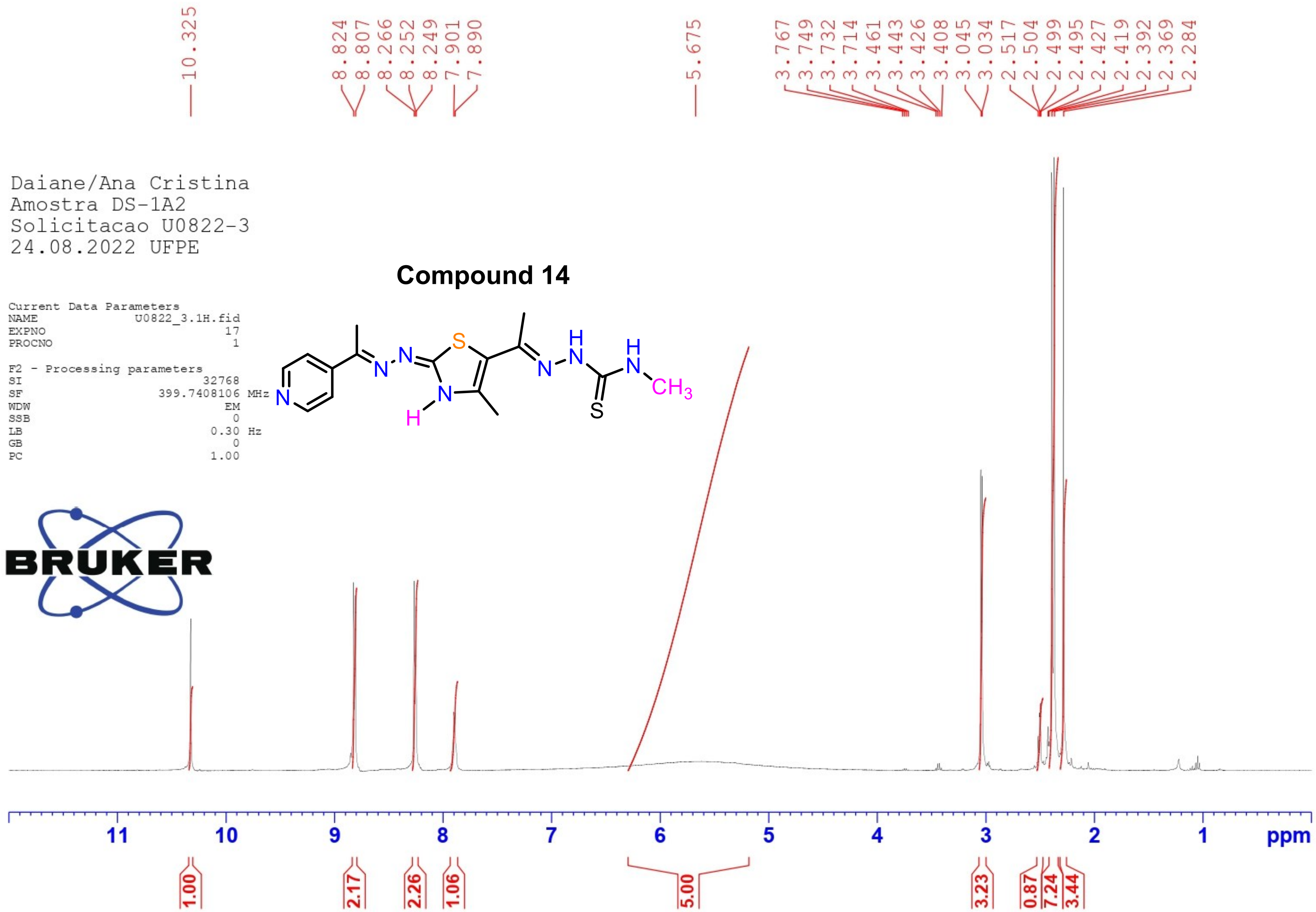

Daiane/Ana Cristina
Amostra DS-1A2
Solicitacao U0822-3
24.08.2022 UFPE
Compound 14
Current Data Parameters
NAME U0822_3.1H.fid
EXPNO 17
PROCNO 1
F2 - Processing parameters
SI 32768
SF 399.7408106 MHz
WDW EM
SSB 0
LB 0.30 Hz
GB 0
PC 1.00
BRUKER
10.325
8.824
8.807
8.266
8.252
8.249
7.901
7.890
5.675
3.767
3.749
3.732
3.714
3.461
3.443
3.426
3.408
3.045
3.034
2.517
2.504
2.499
2.495
2.427
2.419
2.392
2.369
2.284
11 10 9 8 7 6 5 4 3 2 1 ppm
1.00
2.17
2.26
1.06
5.00
3.23
0.87
7.24
3.44

Daiane/Ana Cristina
Amostra DS-1A2
Solicitacao U0822-3
24.08.2022 UFPE

Compound 14

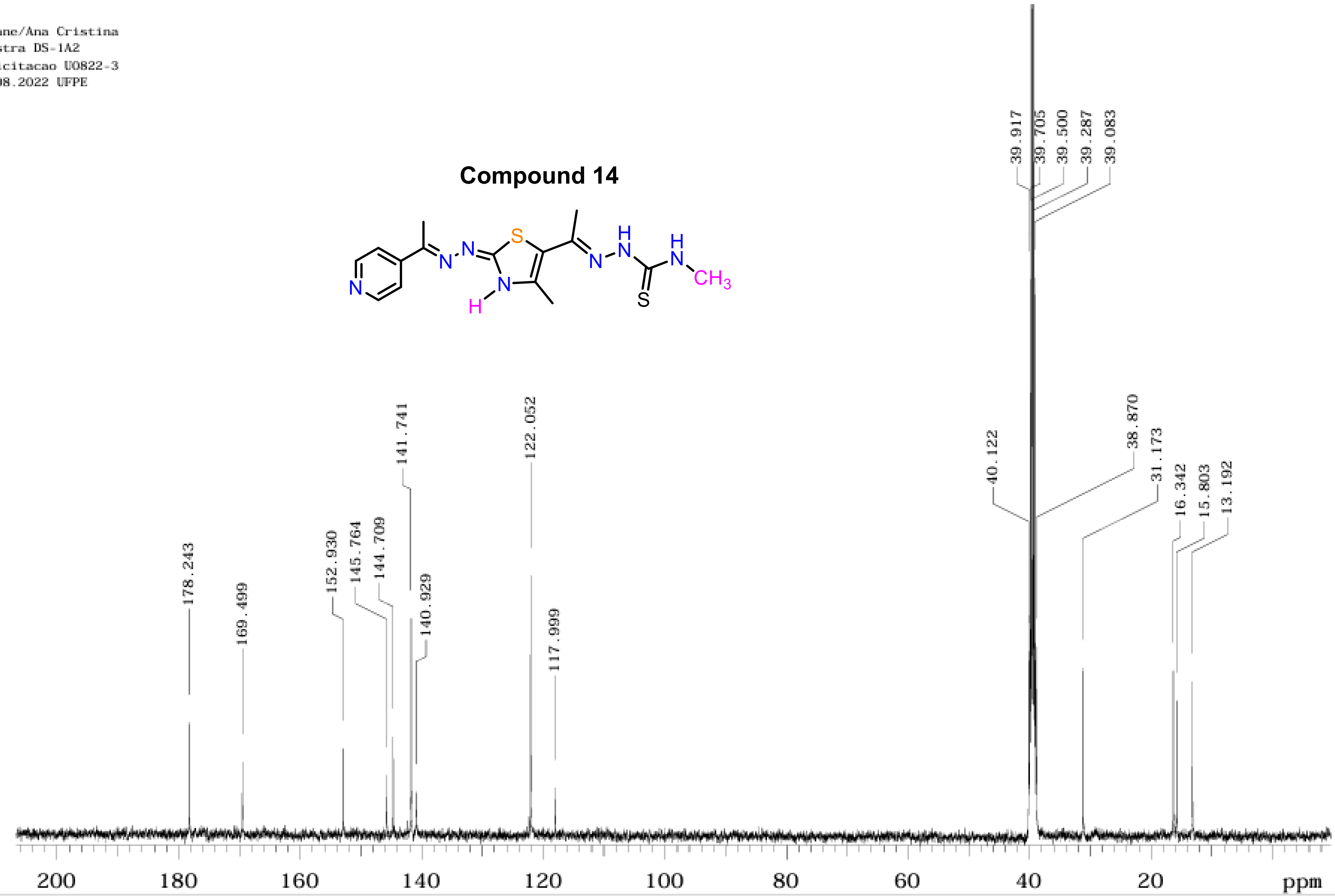

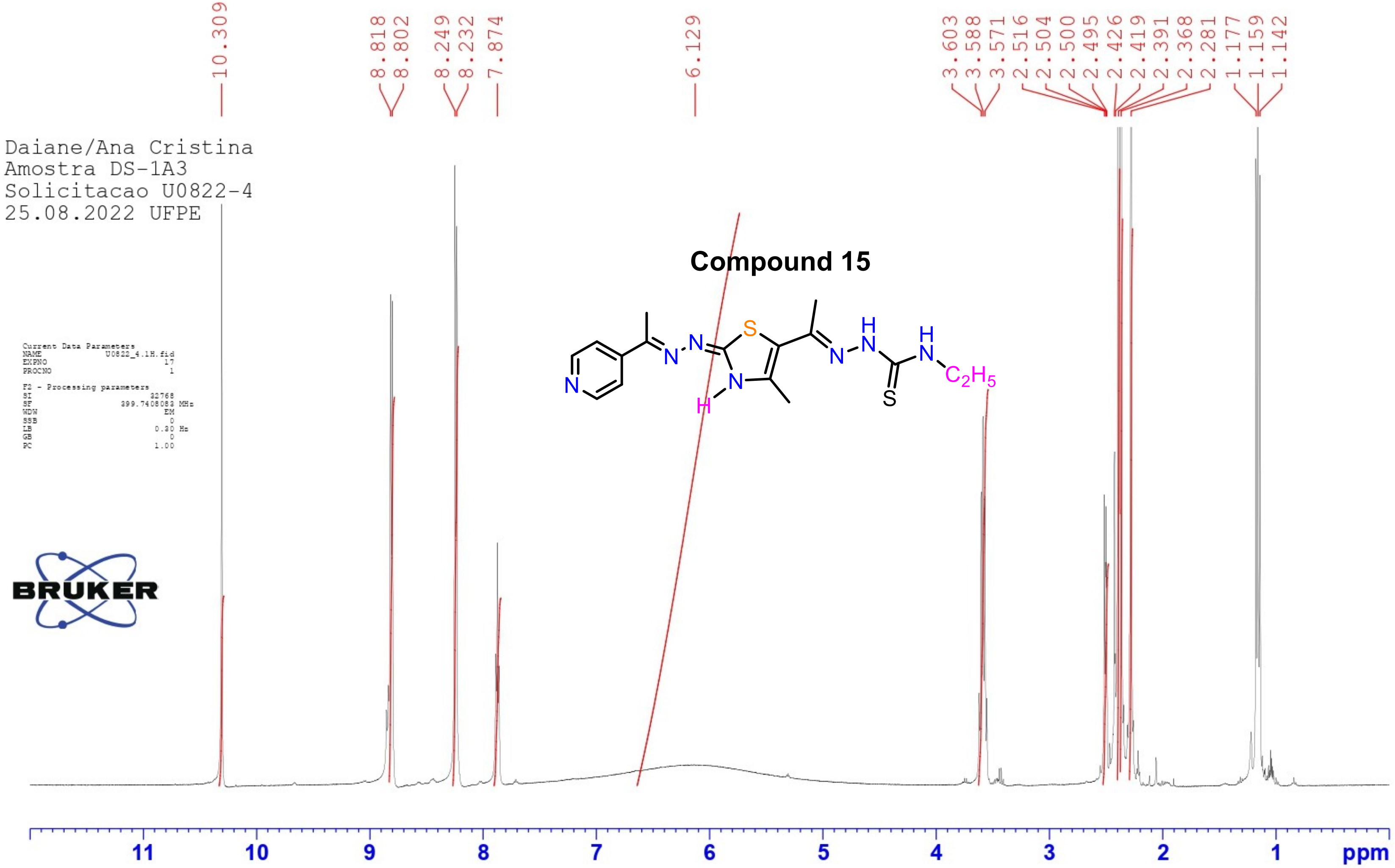
10.309
8.818
8.802
8.249
8.232
7.874
6.129
3.603
3.588
3.571
2.516
2.504
2.500
2.495
2.426
2.419
2.391
2.368
2.281
1.177
1.159
1.142
Daiane/Ana Cristina
Amostra DS-1A3
Solicitacao U0822-4
25.08.2022 UFPE
Compound 15
N
N
N
S
N
H
H
N
N
H
N
C2H5
S
BRUKER
11
10
9
8
7
6
5
4
3
2
1
ppm

Daiane/Ana Cristina
Amostra DS-1A3
Solicitacao U0822-4
24.08.2022 UFPE

Compound 15

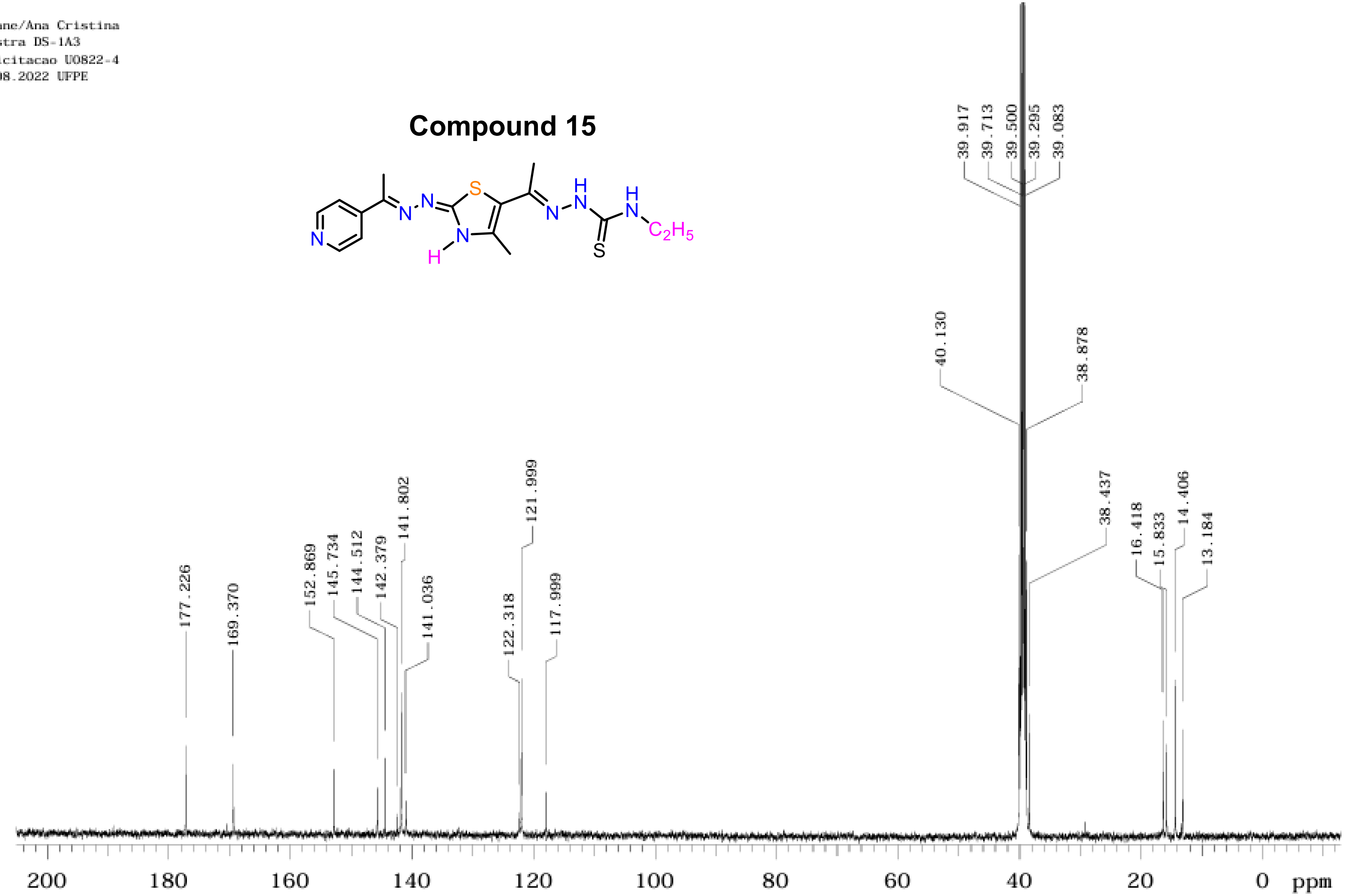

Daiane/Ana Cristina
Amostra DS-1A4
Solicitacao U0822-5
25.08.2022 UFPE

```
Current Data Parameters
NAME           U0822_5.1H.fid
EXPNO                      17
PROCNO                      1

F2 - Processing parameters
SI                      32768
SF            399.7408083 MHz
WDW                        EM
SSB                         0
LB                    0.30 Hz
GB                          0
PC                       1.00
```

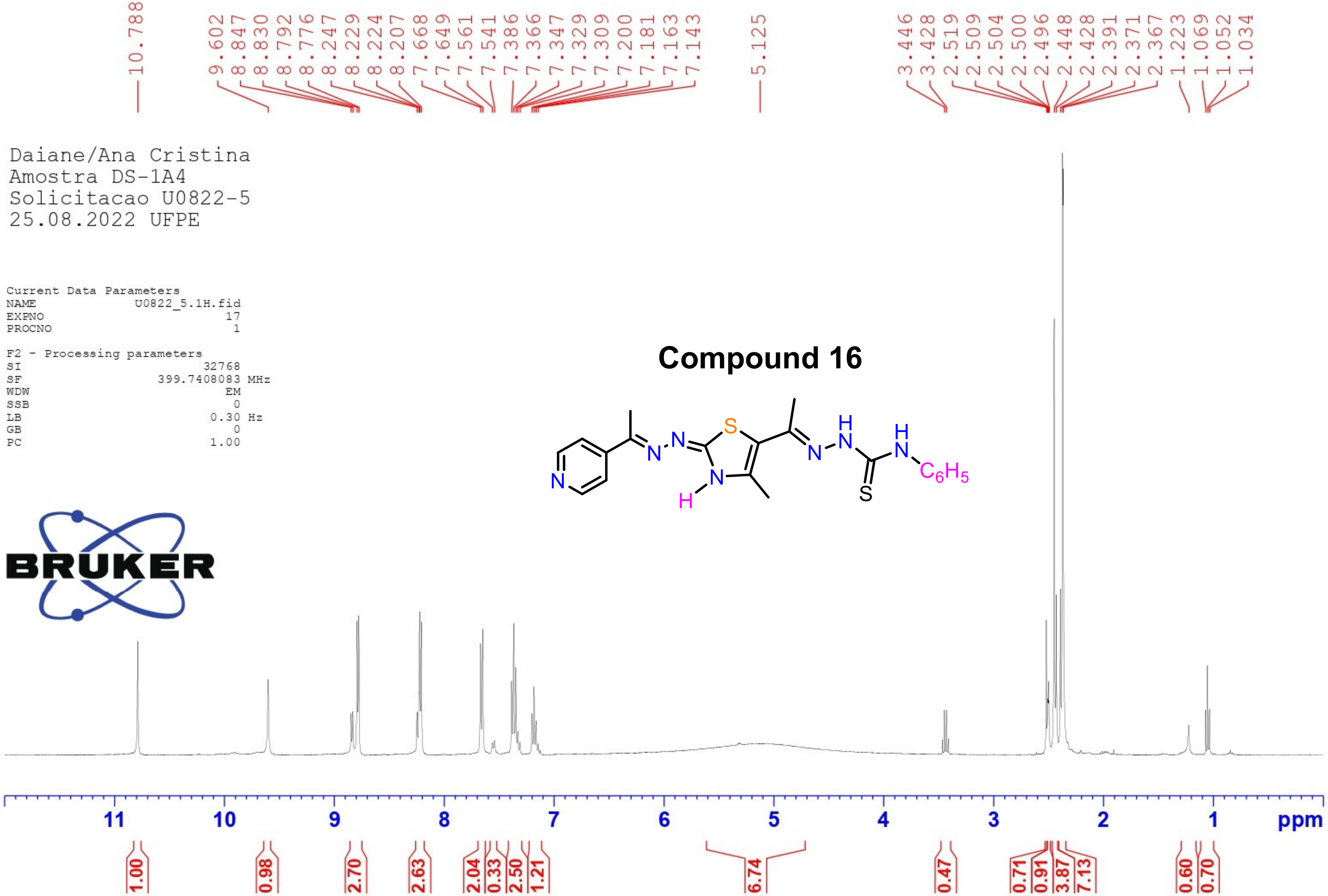

Compound 16

Compound 16

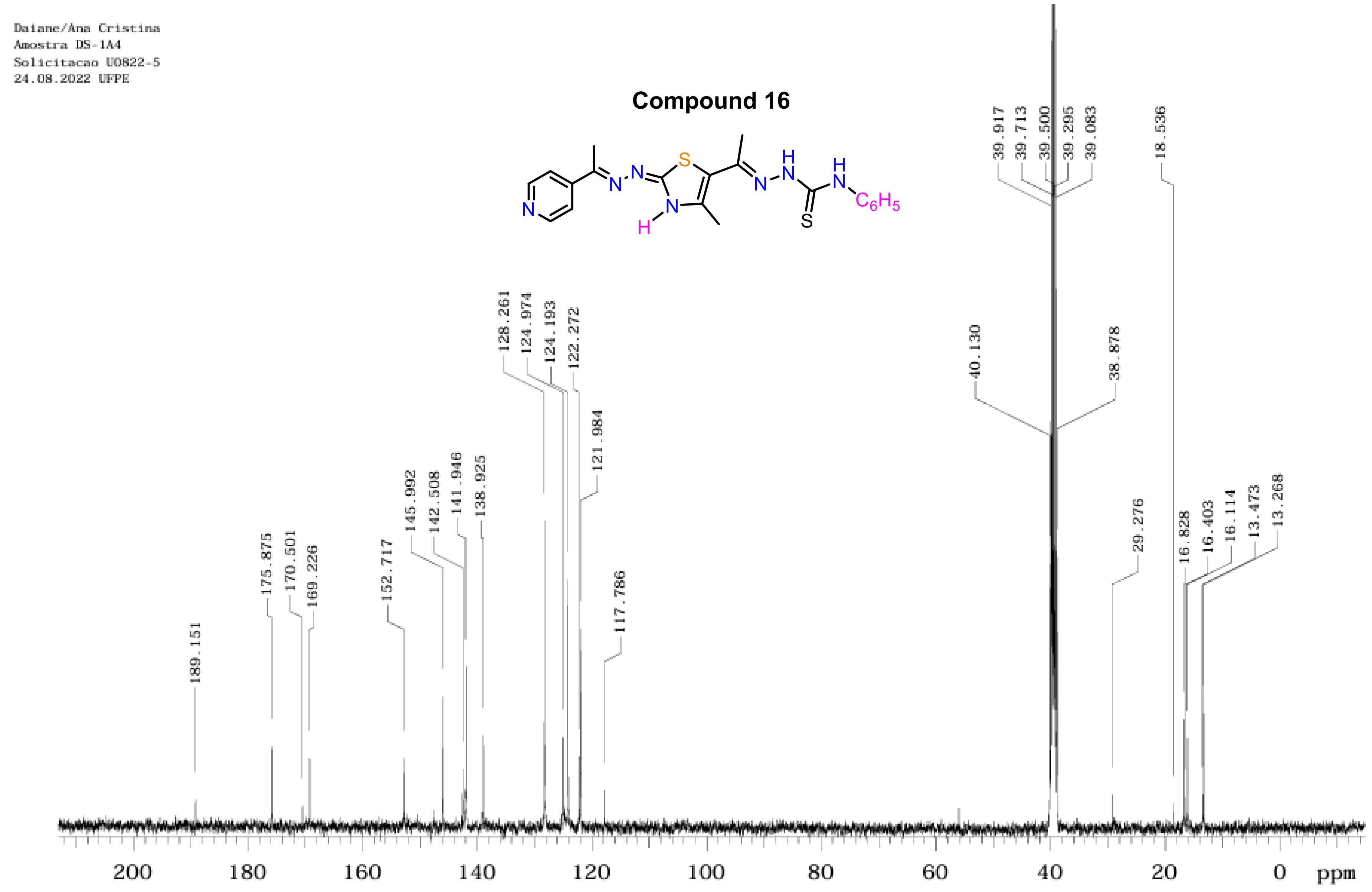

Daiane/Ana Cristina
Amostra DS-1A4
Solicitacao U0822-5
24.08.2022 UFPE
N
N
N
S
N
H
N
H
N
H
N
C6H5
S
189.151
175.875
170.501
169.226
152.717
145.992
142.508
141.946
138.925
128.261
124.974
124.193
122.272
121.984
117.786
40.130
39.917
39.713
39.500
39.295
39.083
38.878
29.276
18.536
16.828
16.403
16.114
13.473
13.268
200
180
160
140
120
100
80
60
40
20
0
ppm

Daiane/Ana Cristina
Amostra DS-1B1
Solicitacao U0822-7
25.08.2022 UFPE

```
Current Data Parameters
NAME              U0822_7.1H.fid
EXPNO                         17
PROCNO                         1

F2 - Processing parameters
SI                         32768
SF                399.7408083 MHz
WDW                           EM
SSB                            0
LB                        0.30 Hz
GB                             0
PC                          1.00
```

Compound 17

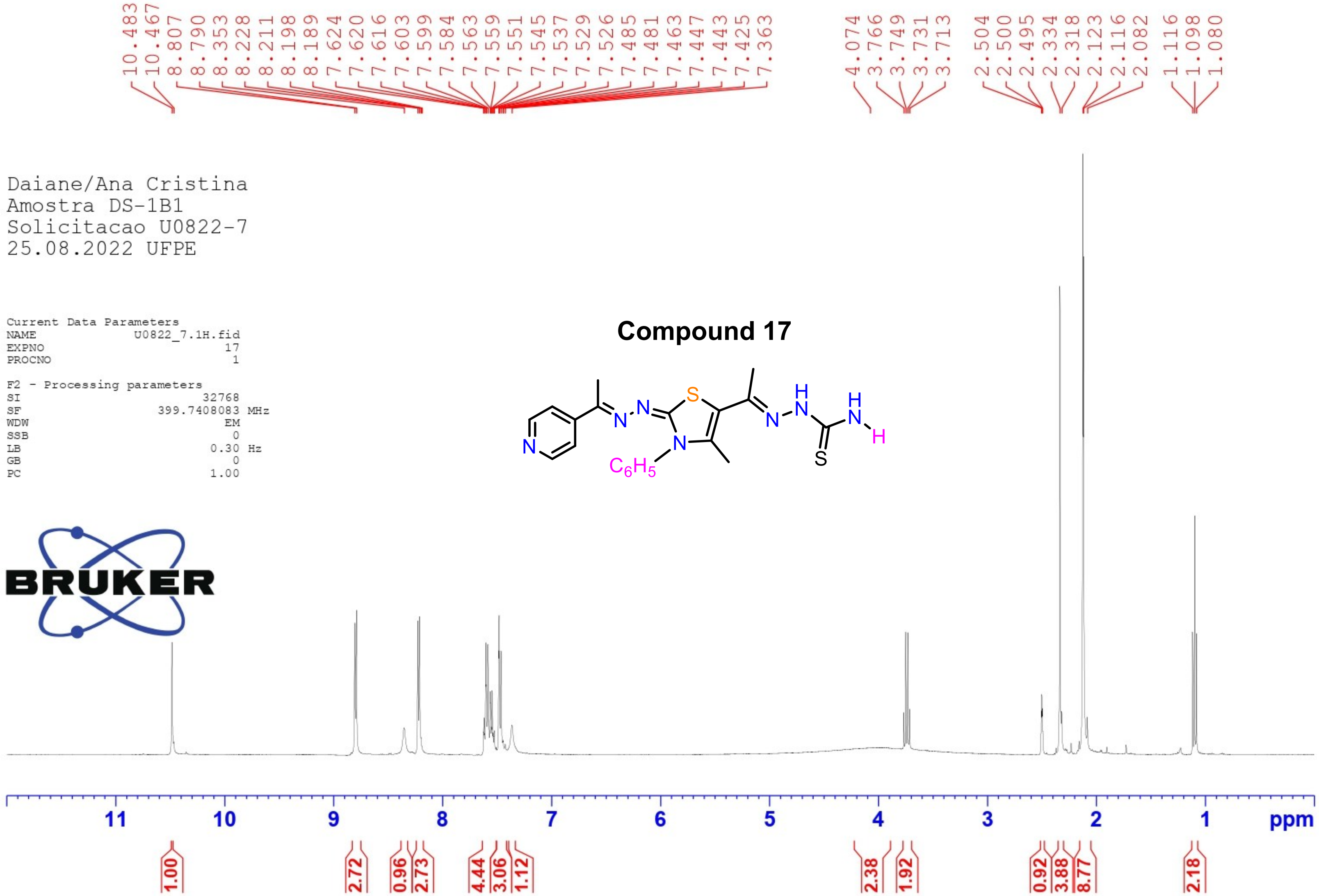

Daiane/Ana Cristina
Amostra DS-1B1
Solicitacao U0822-7
25.08.2022 UFPE

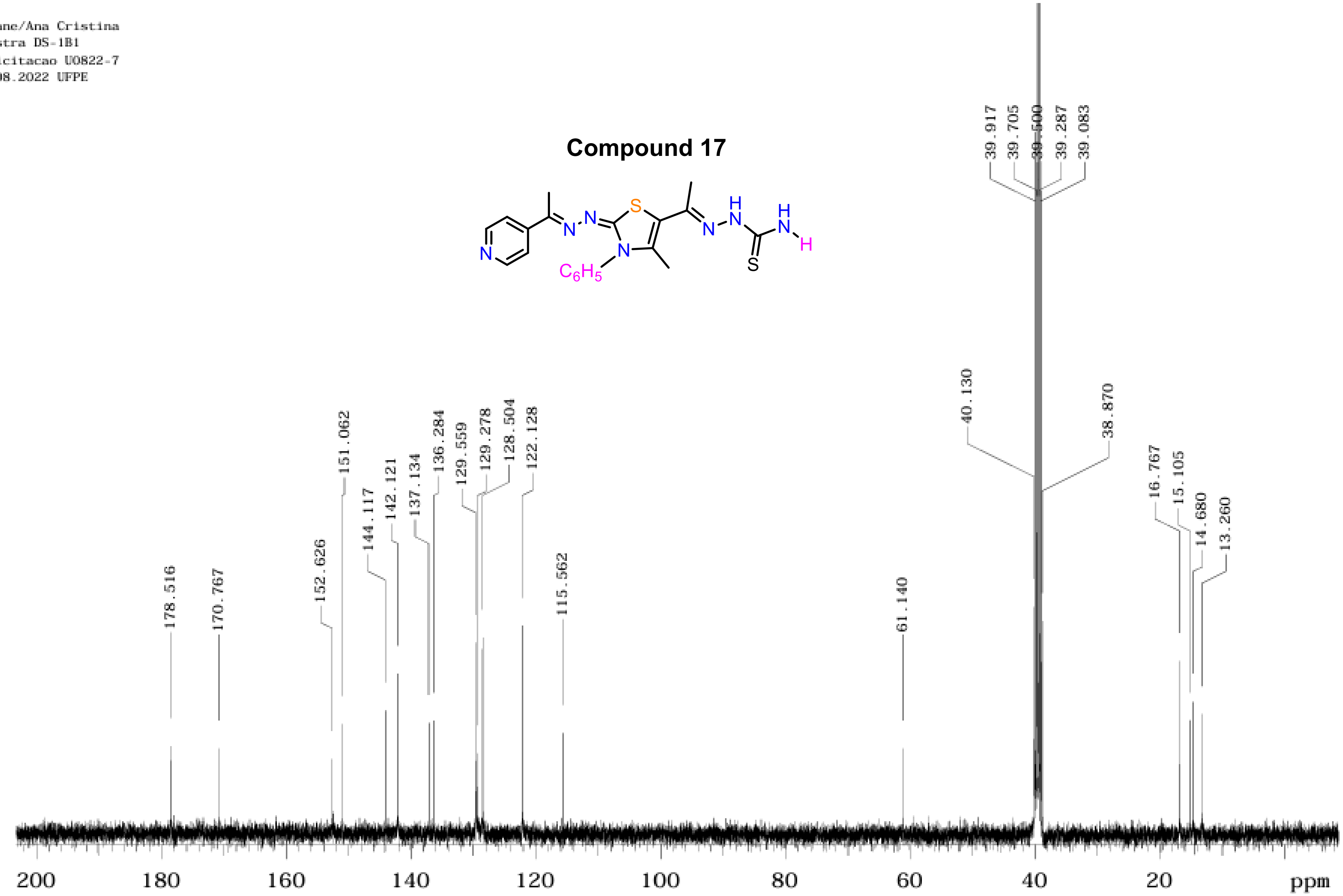

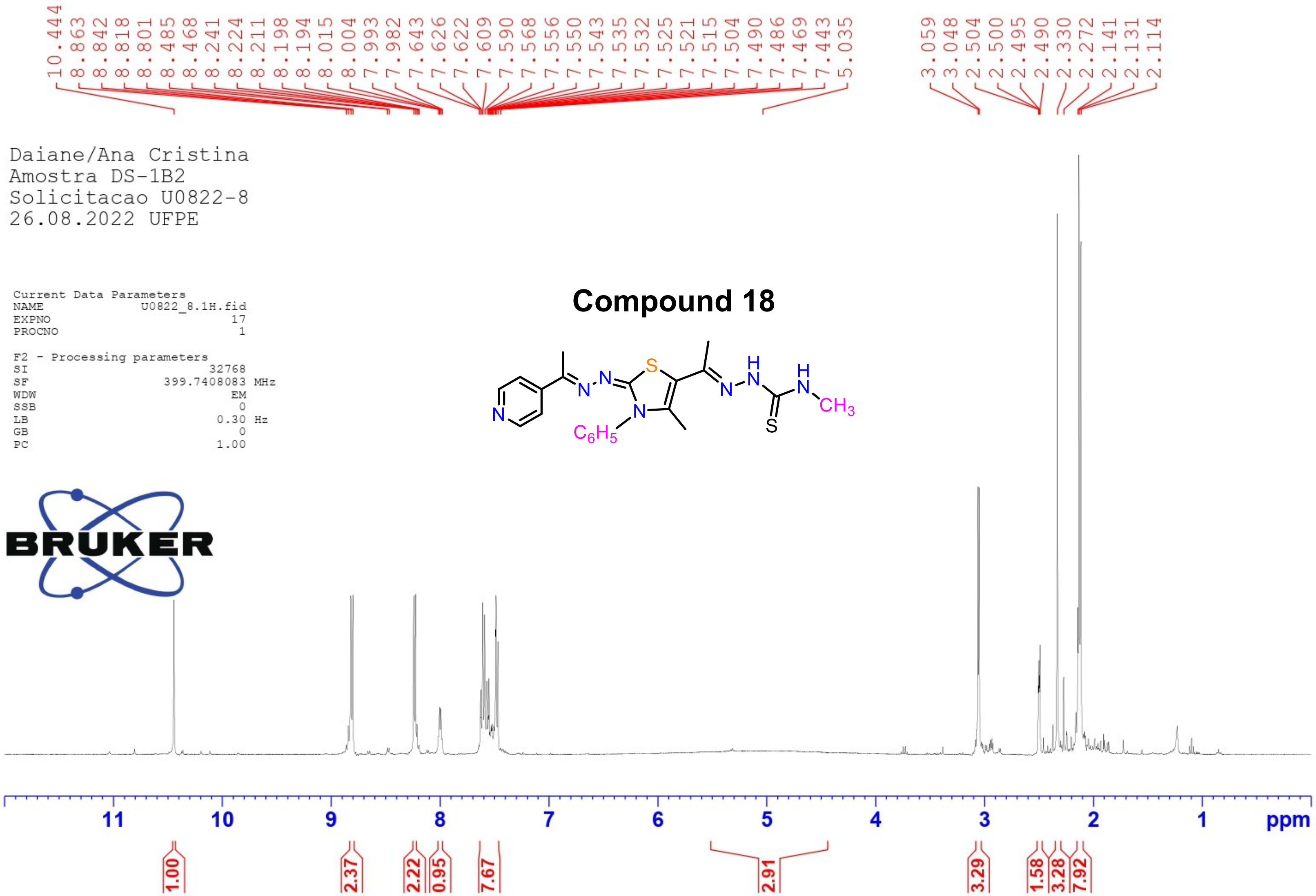
10.444
8.863
8.842
8.818
8.801
8.485
8.468
8.241
8.224
8.211
8.198
8.194
8.015
8.004
7.993
7.982
7.643
7.626
7.622
7.609
7.590
7.568
7.556
7.550
7.543
7.535
7.532
7.525
7.521
7.515
7.504
7.490
7.486
7.469
7.443
5.035
3.059
3.048
2.504
2.500
2.495
2.490
2.330
2.272
2.141
2.131
2.114
Daiane/Ana Cristina
Amostra DS-1B2
Solicitacao U0822-8
26.08.2022 UFPE
Current Data Parameters
NAME U0822_8.1H.fid
EXPNO 17
PROCNO 1
F2 - Processing parameters
SI 32768
SF 399.7408083 MHz
WDW EM
SSB 0
LB 0.30 Hz
GB 0
PC 1.00
Compound 18
S
N
N
N
H
N
H
N
N
N
CH3
C6H5
S
BRUKER
11
10
9
8
7
6
5
4
3
2
1
ppm
1.00
2.37
2.22
0.95
7.67
2.91
3.29
1.58
3.28
7.92

Daiane/Ana Cristina
Amostra DS-1B2
Solicitacao U0822-8
26.08.2022 UFPE

Compound 18

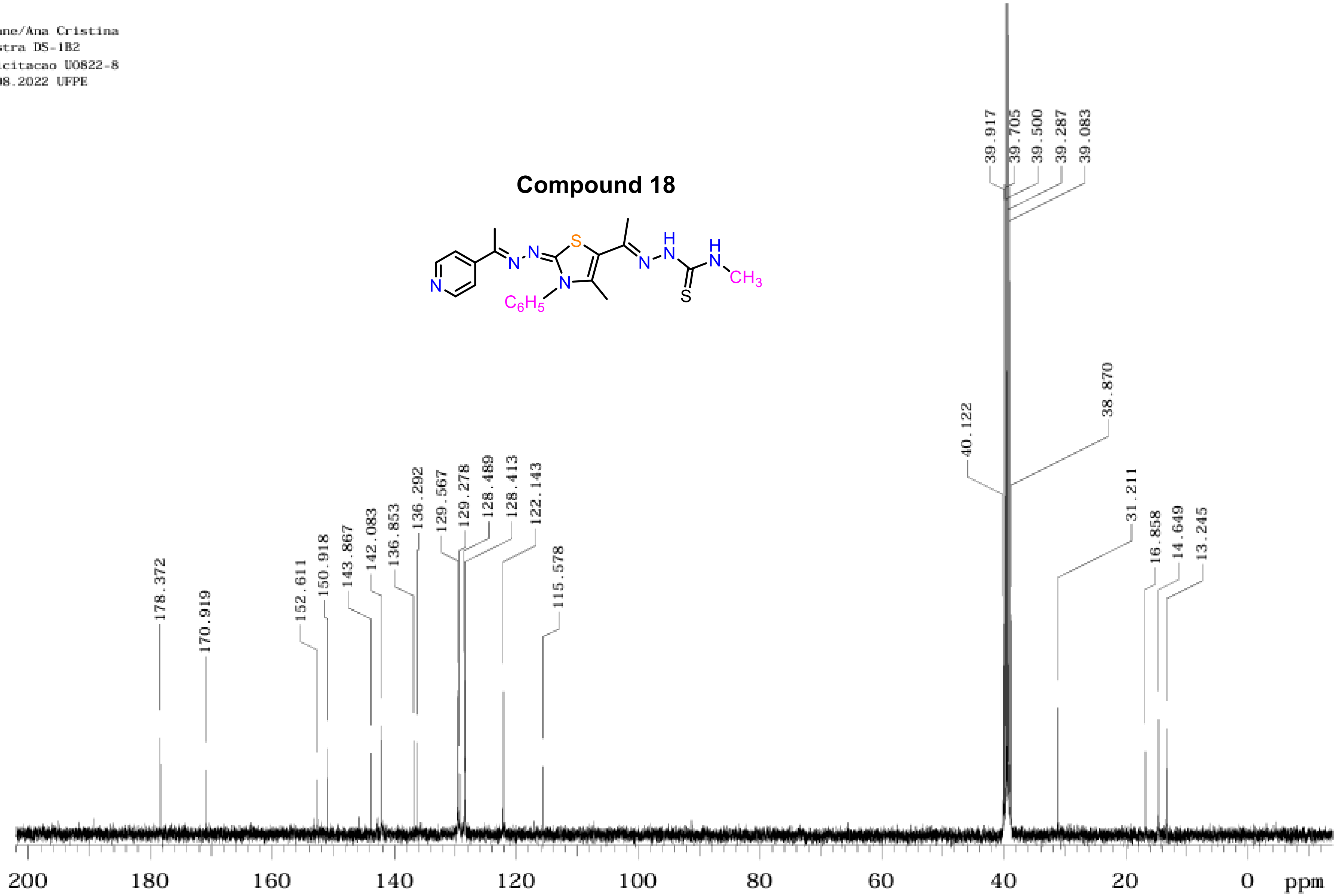

Daiane/Ana Cristina
Amostra DS-1B3
Solicitacao U0822-9
26.08.2022 UFPE

```
Current Data Parameters
NAME             U0822_9.1H.fid
EXPNO                        17
PROCNO                        1

F2 - Processing parameters
SI                        32768
SF              399.7408083 MHz
WDW                          EM
SSB                           0
LB                      0.30 Hz
GB                            0
PC                         1.00
```

Compound 19

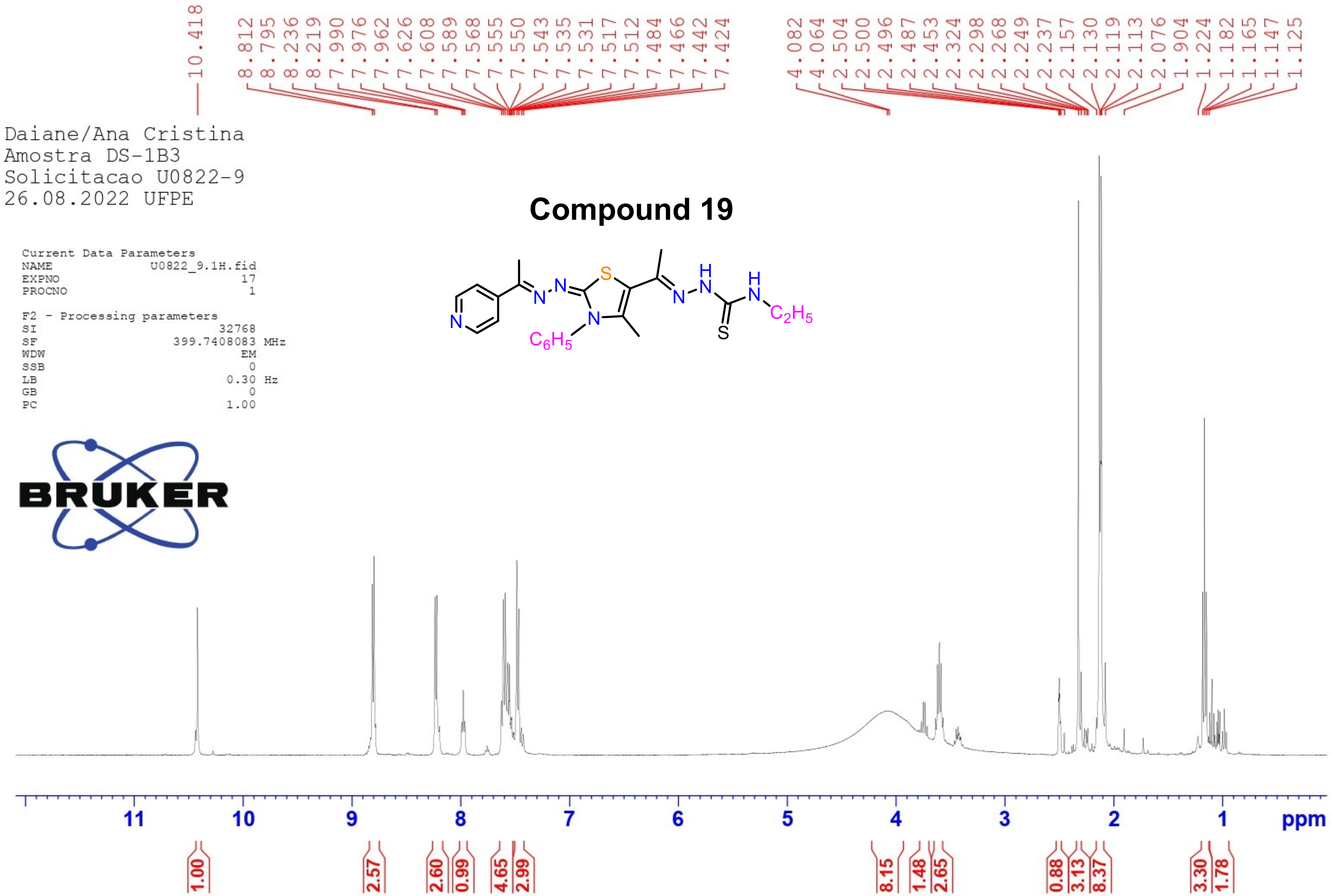

Daiane/Ana Cristina
Amostra DS-1B3
Solicitacao U0822-9
26.08.2022 UFPE

Compound 19

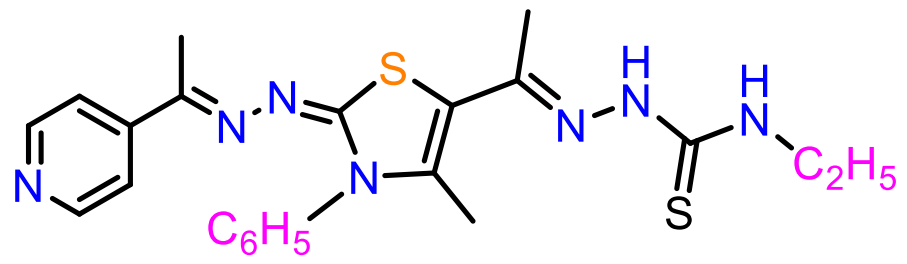

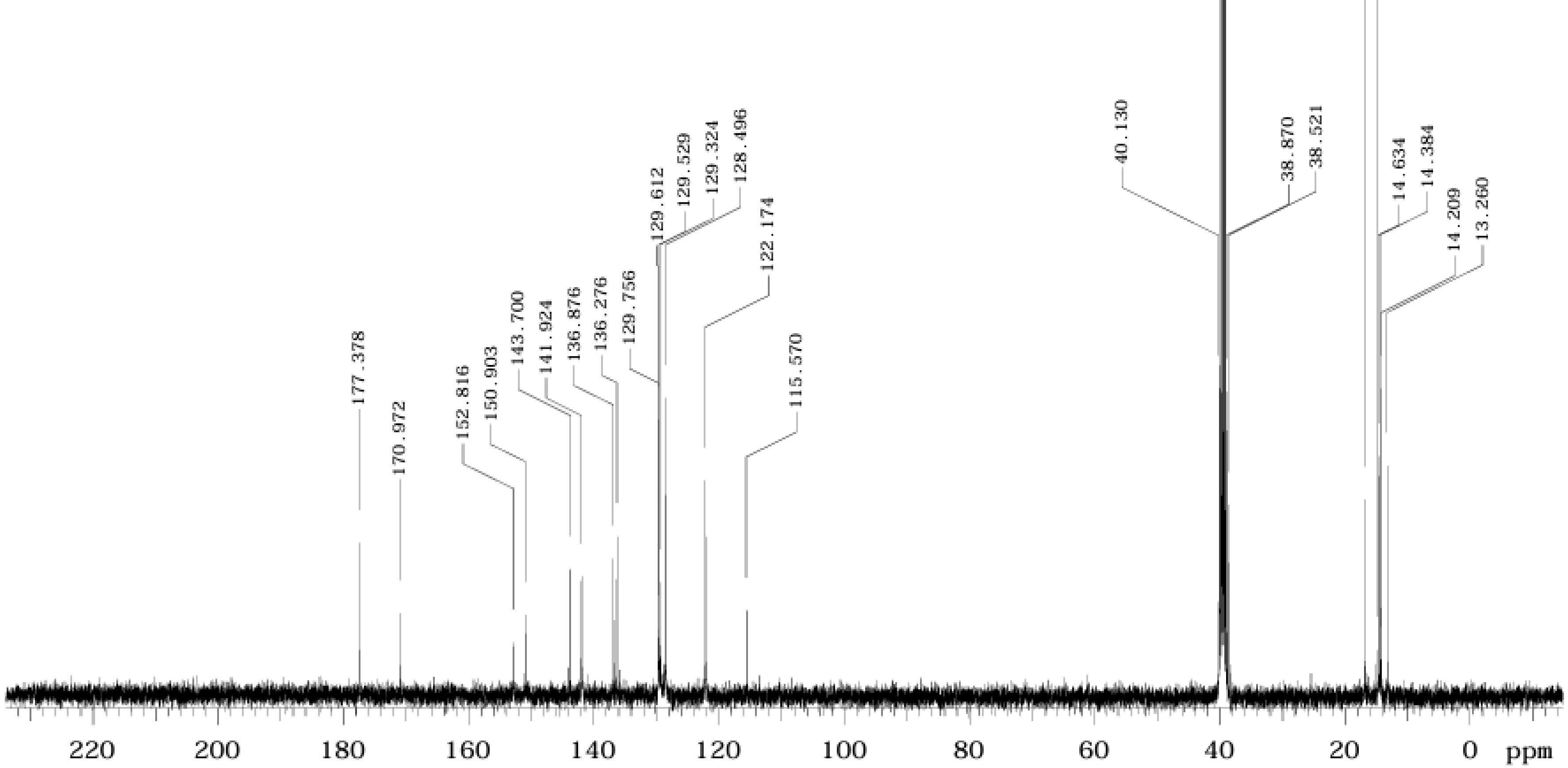

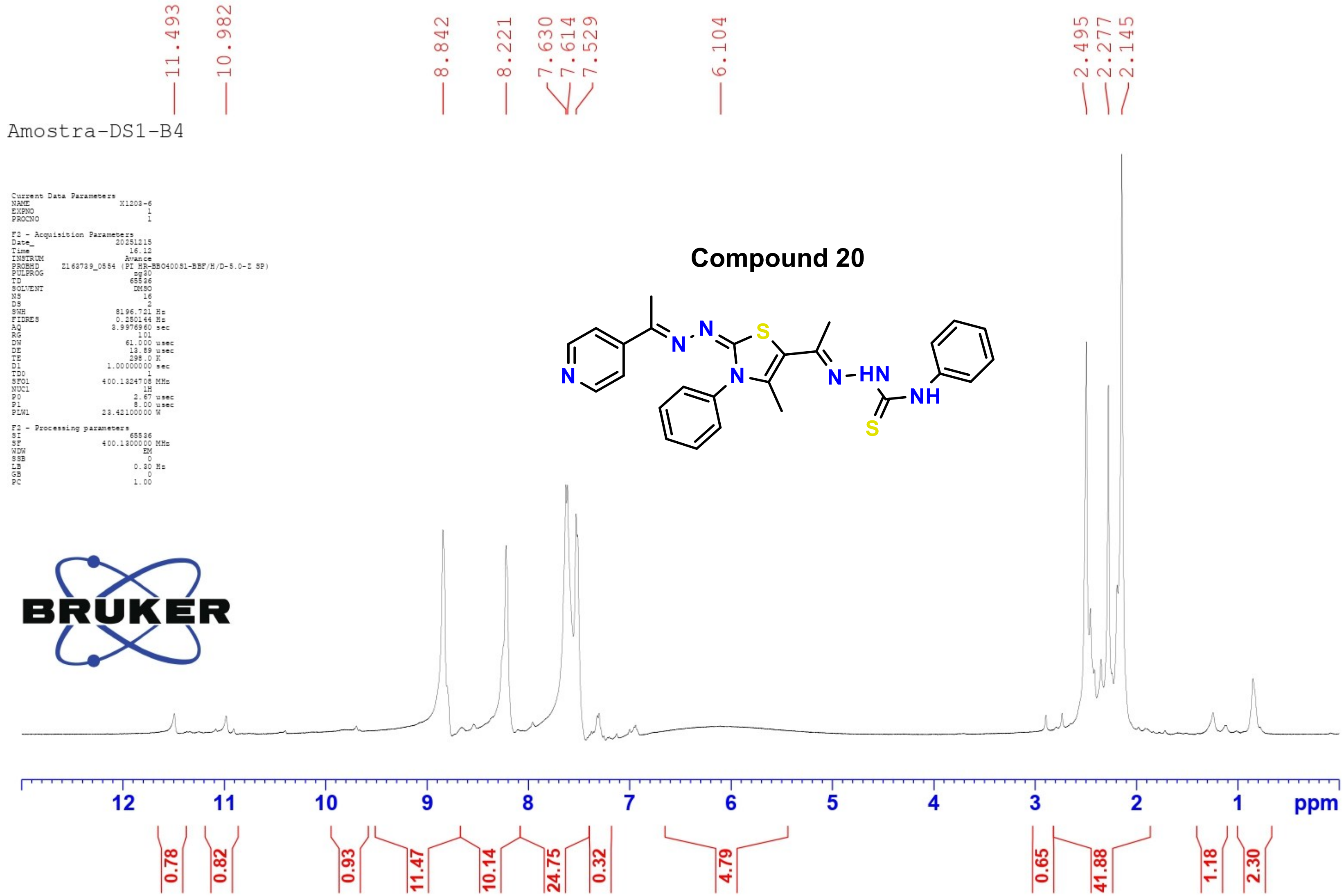
11.493
10.982
8.842
8.221
7.630
7.614
7.529
6.104
2.495
2.277
2.145
Amostra-DS1-B4
Current Data Parameters
NAME X1203-6
EXPNO 1
PROCNO 1
F2 - Acquisition Parameters
Date_ 20251215
Time 16.12
INSTRUM Avance
PROBHD Z163739_0554 (PI HR-BBO400S1-BBF/H/D-5.0-Z SP)
PULPROG zg30
TD 65536
SOLVENT DMSO
NS 16
DS 2
SWH 8196.721 Hz
FIDRES 0.250144 Hz
AQ 3.9976960 sec
RG 101
DW 61.000 usec
DE 13.89 usec
TE 298.0 K
D1 1.00000000 sec
TD0 1
SFO1 400.1324708 MHz
NUC1 1H
P0 2.67 usec
P1 8.00 usec
PLW1 23.42100000 W
F2 - Processing parameters
SI 65536
SF 400.1300000 MHz
WDW EM
SSB 0
LB 0.30 Hz
GB 0
PC 1.00
Compound 20
N
N
N
S
N
N
HN
NH
S
12
11
10
9
8
7
6
5
4
3
2
1
ppm
0.78
0.82
0.93
11.47
10.14
24.75
0.32
4.79
0.65
41.88
1.18
2.30

Compound 20

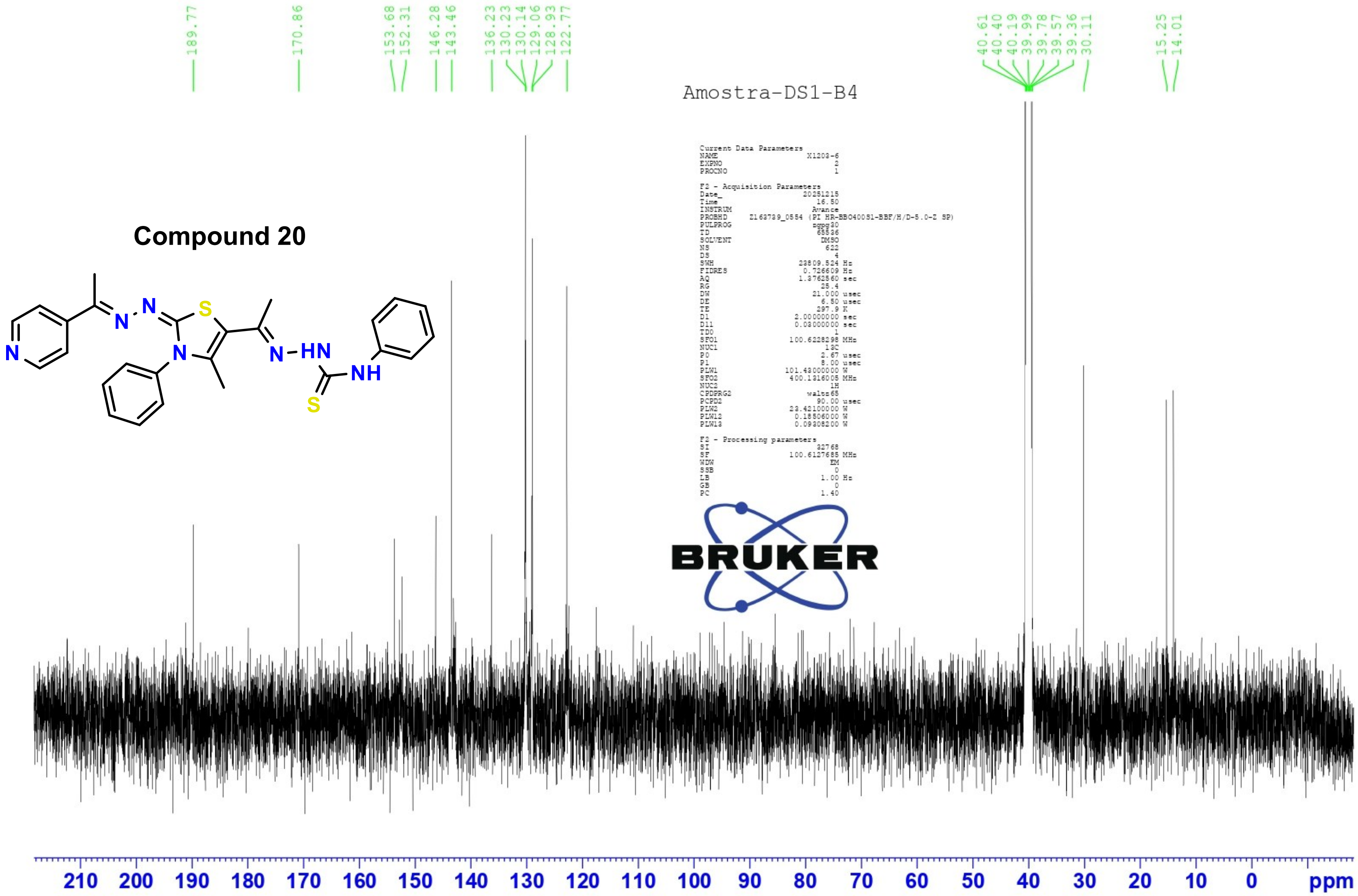

N
N
N
S
N
N
HN
NH
S
189.77
170.86
153.68
152.31
146.28
143.46
136.23
130.23
130.14
129.06
128.93
122.77
40.61
40.40
40.19
39.99
39.78
39.57
39.36
30.11
15.25
14.01
Amostra-DS1-B4
Current Data Parameters
NAME X1203-6
EXPNO 2
PROCNO 1
F2 - Acquisition Parameters
Date_ 20251215
Time 16.50
INSTRUM Avance
PROBHD Z163739_0554 (PI HR-BBO400S1-BBF/H/D-5.0-Z SP)
PULPROG zgpg30
TD 65536
SOLVENT DMSO
NS 622
DS 4
SWH 23809.524 Hz
FIDRES 0.726609 Hz
AQ 1.3762560 sec
RG 25.4
DW 21.000 usec
DE 6.50 usec
TE 297.9 K
D1 2.00000000 sec
D11 0.03000000 sec
TD0 1
SFO1 100.6228298 MHz
NUC1 13C
P0 2.67 usec
P1 8.00 usec
PLW1 101.43000000 W
SFO2 400.1316005 MHz
NUC2 1H
CPDPRG2 waltz65
PCPD2 90.00 usec
PLW2 23.42100000 W
PLW12 0.18506000 W
PLW13 0.09308200 W
F2 - Processing parameters
SI 32768
SF 100.6127685 MHz
WDW EM
SSB 0
LB 1.00 Hz
GB 0
PC 1.40
210 200 190 180 170 160 150 140 130 120 110 100 90 80 70 60 50 40 30 20 10 0 ppm
